# Dementia and Diet, Methodological and Statistical Issues: A Pilot Study

**DOI:** 10.3389/fnagi.2022.606424

**Published:** 2022-07-06

**Authors:** Mark Stecker, Mona Stecker, Allison B. Reiss, Lora Kasselman

**Affiliations:** ^1^Fresno Institute of Neuroscience, Fresno, CA, United States; ^2^Department of Medicine, NYU Long Island School of Medicine, Mineola, NY, United States

**Keywords:** diet, dementia, rice, prevalence, risk factors, multivariate analysis, statistical simulations

## Abstract

There is conflicting information on the relationship between diet and dementia. The purposes of this pilot study were twofold. First, to use publicly available data regarding food consumption (United Kingdom Family Food), dementia, risk and demographic factors to find relationships between the consumption of various foods to dementia prevalence. The second purpose was to identify elements of study design that had important effects on the results. Multiple analyses were performed on different data sets derived from the existing data. Statistical testing began with univariate correlation analyses corrected for multiple testing followed by global tests for significance. Subsequently, a number of multivariate techniques were applied including stepwise linear regression, cluster regression, regularized regression, and principal components analysis. Permutation tests and simulations highlighted the strength and weakness of each technique. The univariate analyses demonstrated that the consumption of certain foods was highly associated with the prevalence of dementia. However, because of the complexity of the data set and the high degree of correlation between variables, different multivariate analyses yielded different results, explainable by the correlations. Some factors identified as having potential associations were the consumption of rice, sugar, fruit, potatoes, meat products and fish. However, within a given dietary category there were often a number of different elements with different relations to dementia. This pilot study demonstrates some critical elements for a future study: (1) dietary factors must be very narrowly defined, (2) large numbers of cases are needed to support multivariable analyses. (3) Multiple statistical methods along with simulations must be used to confirm results.

## Introduction

Understanding the factors that modify the prevalence of dementia is difficult since dementia may result from many different mechanisms that are not only operative over long periods of time, but are also modified by individual genetic differences (Beeri, 2019). Despite this, exploring the effects of environmental exposures may provide some insight into mechanisms of dementia (Killin et al., 2016). One of the strongest environmental exposures over prolonged time periods is diet (Sindi et al., 2018), although dietary intake is a very complex variable that can be difficult to quantify. One approach to quantifying diet is to study specific nutritional regimens (Mediterranean Diet, etc.), and then compare the effects of different degrees of adherence to these nutritional regimens on probability of developing dementia. Another approach to quantifying diet is to collapse diet into a few general descriptors such as total nutrient intakes and analyze them (Shim et al., 2014). Both of these approaches are associated with significant risks of selection bias and statistical heterogeneity. The third approach is to take existing populations and make as detailed an examination of as many elements of the diet as possible for each population and compare the prevalence of dementia in those populations. The first two approaches are simpler, and can be more powerful, especially if there is evidence that specific dietary elements are correlated to the progression of dementia (Abate et al., 2017). The advantage of the third approach is that it does not include any intrinsic bias that certain dietary elements may have an effect on dementia. However, statistical issues arise when there are a large number of highly correlated explanatory variables. This makes such studies difficult to implement and interpret. Understanding these issues by utilizing different data sets and analyses is one important focus of this paper. The next section of the introduction will take a very broad view of the literature describing consecutively, studies that use very broad dietary categories and then later studies that use very narrow dietary categories. The results of this paper will be contrasted with those obtained in these studies.

There is some suggestive evidence that the Mediterranean Diet (Estruch et al., 2018; Rees et al., 2019; Hu et al., 2020) and its various iterations along with the DASH (Dietary Approach to Stop Hypertension) diet (Bathrellou et al., 2019; Chiavaroli et al., 2019) may have protective effects on the cardiovascular system. This theory led to the idea that the DASH and Mediterranean Diets may be neuroprotective (Blumenthal et al., 2019; Shannon et al., 2019; van den Brink et al., 2019). Sofi et al. (2008) conducted a meta-analysis on the beneficial effects of adherence to the Mediterranean diet on mortality and the prevention of major chronic diseases, including Alzheimer’s disease. This meta-analysis looked at 12 prospective cohort studies which included a total of 1,574,299 subjects followed for a time ranging from 3 to 18 years. The conclusions of this meta-analysis showed that a greater adherence to a Mediterranean diet was associated with a significant improvement in health status, as seen by a reduction in overall mortality (9%), mortality from cardiovascular diseases (9%), incidence of, or mortality from, cancer (6%), and incidence of Parkinson’s disease and Alzheimer’s disease (13%). A different meta-analysis by Petersson and Philippou (2016) indicated that a number of studies demonstrated no significant effects of the Mediterranean diet on cognitive function although the evidence was in favor of an effect. Luciano et al. (2017) assumed a different approach and assessed the association between Mediterranean-type diet and change in MRI volumetric measures and mean cortical thickness across a 3-year period in older age (73–76 years) participants. A total of 927 participants completed a dietary questionnaire and then underwent MRI scanning at two time points. The authors concluded that lower adherence to the Mediterranean-type diet in an older cohort was predictive of cerebral atrophy over a 3-year interval. The authors further concluded that some, or all components (possibly in combination) of a Mediterranean-type diet were associated with less brain atrophy as evidenced through MRI.

The Global Burden of Disease Study of 2016 (Gbd 2016 Dementia Collaborators., 2019) provided a systematic analysis exploring the global, regional and national burden of Alzheimer’s disease and other dementias. They found large differences in dementia rates in different countries but also found sufficient evidence to link high BMI, high fasting plasma glucose and high intake of sugar-sweetened beverages to dementia. Similarly, Pistollato et al. (2018) suggested on the basis of a literature review that dementia, diabetes, obesity, insulin resistance, and cardiovascular disease are highly connected; and that healthy dietary patterns, characterized by a high intake of plant-based foods, probiotics, antioxidants, soy beans, nuts, and omega-3 polyunsaturated fatty acids, and a low intake of saturated fats, animal-derived proteins, and refined sugars, have been shown to decrease the risk of neurocognitive impairments and eventually the onset of AD. In addition, Pistollato et al. (2018) reviewed the literature as it pertained to multi-component dietary patterns including the Mediterranean diet (MeDi), DASH diet and Mediterranean diet Intervention for Neurodegenerative Delay (MIND) and their effects on the maintenance of cognitive performance. The authors concluded based on their review of the literature which included several *in vivo* studies (Pistollato et al., 2018), that multi-component, healthy dietary patterns, such as MeDi, DASH and MIND diets, were more effective than single nutrient intervention strategies for the maintenance of neurocognition and the reduction of dementia and dementia risk (Pistollato et al., 2018). In an alternative approach, Akbaraly et al. (2019) administered a diet questionnaire to examine whether a midlife diet was associated with subsequent risk for dementia. A food frequency questionnaire was used to derive the Alternate Healthy Eating Index (AHEI) in 8225 participants. This was a long-term prospective cohort study that began in 1985. Dietary intake was assessed in 1991–1993, 1997–1999, and 2002–2004. The participants were followed-up for incident dementia until March of 2017. This study (Akbaraly et al., 2019) did not find that diet quality was significantly associated with subsequent risk for dementia. de Crom et al. (2022) noted positive effects of the MIND diet. Munoz-Garcia et al. (2020) concurred with this and in addition found no effect of the DASH diet. Recent studies from Saji et al. (2021) and Wu et al. (2021), respectively have suggested that Japanese and Nordic diets may also reduce risk of dementia.

Although studies focused on general aspects of diet show conflicting results about the effect of diet on dementia, investigations focused on a particular nutrient such as polyphenols by Colizzi (2018) and the carotenoid lycopene by Crowe-White, Phillips and Ellis (Crowe-White et al., 2019) have failed to show convincing effects. While intake of vitamins B12 and B6 has not been associated with changes in dementia rates, increased folic acid intake was associated with a lower rate of dementia (Lefevre-Arbogast et al., 2016). Fish intake, but not omega-3 fatty acid intake, has been associated with dementia (Wu et al., 2015). On the other hand, a recent Cochrane review (Rutjes et al., 2018) suggested that there is no relationship of vitamin or mineral intake to cognitive function. Other studies (Dearborn-Tomazos et al., 2019) have also suggested either no effect or a weak effect of diet. Charisis et al. (2021) suggested that a pro-inflammatory diet as measured by the diet inflammatory index (Hayden et al., 2017) was associated with an increased risk of dementia.

The inability of studies constructed as above to find a strong and reproducible relationship between diet and dementia may, in part, be related to their focus on a few very broad diet categories. It is possible that different foods within a category may have very different yet important effects. The use of the comprehensive dietary database contained in the United Kingdom family food study may be able to shed light on this. However, the use of such a large database raises methodological and statistical issues. The specific focus of this paper is a preliminary exploration of these issues.

## Materials and Methods

### Rationale

The data in this paper come from two different but related analyses based on the United Kingdom family food study and information from the United Kingdom National Health Service (NHS). Both are described in much more detail in Table 1 and Supplementary Appendix. The reason for incorporating both studies into a single paper was to obtain a clearer picture of the effects of slightly different methodologies for abstracting data from the very large available data sets on the study results. It also highlights the potential problems with statistical inference on a database containing a large number of variables and a small number of cases. In such a study it is possible to find many different statistically significant results. Confidence in those results is much higher if two slightly different studies reach the same conclusions. Any difference between the studies can be used to highlight methodological issues.

**TABLE 1:** Comparison of the two studies performed in this paper on diet and dementia using the United Kingdom family food data.

Element	Study 1	Study 2	Comment
United Kingdom Family Food: Years	2016–2017	2011, 2012, 2013, 2014, 2015, 2015, 2016, 2017, 2018, 2018, and 2019	Although data was available from 2001–2019 only the listed years had complete data on dementia prevalence
Dementia index	Fraction of the total population with dementia.	“Dementia QOF”-Fraction of patients seen by a general practitioner with a diagnosis of dementia	Dementia QOF available only for England. Dementia prevalence in other locations came from other data sets (Supplementary Table 21). Fraction of population with dementia was available on limited geographies and time periods. It was interpolated for the first study for Wales, Scotland and Northern Ireland from different years.
Geography	(1) 9 Regions of England (2) Wales (3) Scotland (4) Northern Ireland	(1) 9 Regions of England	In the second study data from the regions of England was reconstructed from data at the Clinical Commissioning Group or Count level (Supplementary Appendix 1.1)
Comorbidities	Entered by hand from various sources (Supplementary Table 21)	Downloaded and processed directly from NHS websites and ONS websites	For study 2, mappings between different geographies in England used to find to construct results that are valid for each region of England (Supplementary Appendix 1.1).
Univariate	(1) Pearson Correlation (2) Spearman Correlation	(1) Pearson Correlation (2) Spearman Correlation (3) Cross tabulation tables (4) Blomqvst, Hoeffding Goodman-Kruskal (5) Regression	For a study with limited cases, univariate comparison are a critical primary result. Although correlations are formally bivariate measures they explore only one explanatory variable at a time and the term univariate will be used. A constant is included in each regression model but not displayed.
Partial Correlations for Food Variables	No	Yes	Removes effects of age, race and gender from the food variables.
Permutation tests for univariate measures	No	Yes	The use of permutation tests increases statistical certainty about the test results.
Exhaustive Regression	Limited	Yes	Time intensive but has low false positive rate. Arbitrary choice of what defines optimal models
Stepwise Multiple Regression	No	Yes	Forward very valuable with ranked variables to reduce false positives but reverse often eliminates likely important variables.
Cluster Regression	No	Yes	Limited value as clustering not biologically based.
Principal Components Analysis	Yes	Limited	Limited Value as clustering not biologically based.
Regularized Regression	No	Yes	
Construction of Single weighted food index	No	Yes	Weights chosen according to the partial correlation coefficients or through an iterative variance minimizing search.
Simulations	No	Yes	For a complex analysis, simulations allow a better picture of the true statistical nature of any process.

### Data Sources

The data for this study were taken from various publicly accessible sites from the United Kingdom that are detailed in Supplementary Appendix Sections 1.1, 1.2, 1.3, and 2.1 along with Supplementary Table 21. As stated on the relevant websites, access to the data used in this study was governed by the Crown copyright which allowed free-reuse of the data on these websites as long as the following citation is present (Public Health England. Public Health Profiles. 12/05/2021^1^ © Crown copyright 2021). The websites also suggest that the following statement be made. “The data is public sector data licensed under the Open Government License v3.0^2^.”

#### United Kingdom Family Food Database

The information on food purchases and nutrients came from the United Kingdom Family Food Survey. The dietary data used in this study are available at the site^3^. The methodology of this study described in detail on the United Kingdom Family Food website (About Family Food., 2014; Sampling., 2015). In brief, the surveys are given to all members of selected households and represent food purchased confirmed by purchase receipts not food consumed over a 2 week period. The purchased food was divided into food that enters the household (“Household Purchases”) and food that was purchased outside the home (“Eating Out”) and then was reported in amounts per person per week. In 2014 the survey collected food information on 121,250 people in 5,144 households in the United Kingdom. The intake of various nutrients both as absolute values and as a percentage of weighted reference intakes was then computed based on the Composition of Foods Integrated Dataset (Compositon of Foods Integrated Dataset [CoFID], 2021). Data was available for each of the 9 Regions of England (North East, North West, Yorkshire and the Humber, East Midlands, West Midlands, East of England, South East, London, South West) as well as the United Kingdom countries: Wales, Scotland and Northern Ireland. Data used in the first study came from the year 2016/2017. The Family Food database contained additional data not used in the first study for the years 2001–2019. The second study used data from the 8 years that could be matched to a complete estimate of dementia for the 9 regions of England (detailed in the Supplementary Appendix 1.2–1.5).

#### Dementia Prevalence

Two slightly different measures of dementia prevalence were used. In the first study, estimates of the prevalence of dementia for the regions of England and the other United Kingdom countries (Wales, Scotland and Northern Ireland) came from different sources (Supplementary Table 21). The total fraction of the population with dementia was computed in England from variables containing the prevalence of dementia in patients above and below 65 in 2019. In the other United Kingdom countries, the prevalence of dementia was taken from 2017 and 2018 (Supplementary Appendix Section 2.1). In the second study, the NHS variable “Dementia QOF” which was the fraction of all patients seen by a general practitioner with a diagnosis of dementia (Supplementary Appendix 1.3.1) was available. For some years, the NHS database also contains an estimated dementia diagnosis rate which was the fraction of expected dementia diagnoses that were actually recorded. The percentage recorded for England was 68.7% and was based on prior more detailed studies in different sub-populations.

#### Other Data

In the first study, for each region/country, the total population, population density, median age, birth rate, death rate and % of population aged 65+ for 2018, were taken from the Office of National Statistics (ONS) (Supplementary Table 21). Ethnicity data and information on the gross disposable household income (GDHI) index for 2017 was taken from the sources in Supplementary Table 21. The data from the regions of England for hypertension, diabetes, coronary heart disease, stroke, obesity, depression, smoking and physical inactivity were taken from the sources shown in Supplementary Table 21. In the second study a more limited set of medical comorbidity information and population characteristics was included (Supplementary Appendix 1.3.2 and 1.3.3) so that there could be a complete set of data for the maximum number of time periods.

### Statistical Analysis

Before analysis, all variables with missing or incomplete data were eliminated. A single dependent variable (dementia prevalence) was identified. The other variables (explanatory/independent variables) were classed into 3 groups: “All,” “Demographics” (Includes medical comorbidity variables), and “Food” which includes all the food variables. There were 22 covariates and 657 diet/nutrition related target variables analyzed in the first study and 507 dietary factors and 12 demographic/comorbidity variables in the second study. There were fewer variables in the second study because even one missing data point in any of the years selected caused elimination of that variable. The first study included only 9 cases (one for each region of England) but the second study included 72 cases (the “full” data set) over 8 different years. At times in the second study reference was made the set of data “averaged” over the different times so that there were only 9 cases. This will be referred to as the “averaged” data set.

#### Correlation (Univariate) Analysis

The first step in this process was determining the degree of correlation between each explanatory variable and the dementia prevalence. Although correlation measures always use two variables and hence from that perspective are bivariate from the data perspective only one explanatory variable was considered at a time. For this reason, the term “univariate” will be used rather than the more proper term “bivariate.” The first study relied only on the Pearson and Spearman correlation tests. The second study included many other tests (Table 1) of association including partial correlation testing after removal of the effects of age, race and gender from both the explanatory variables and the dementia prevalence estimate. For the partial correlation analysis, separate linear regression analyses were performed using age, race and gender as independent variables and the explanatory variable or dementia prevalence as the dependent variable. The residuals resulting from these linear regression analyses were then tested for correlation with the Spearman rank correlation tests. A strong correlation indicates that the any relationship between the two variables is unlikely to be related to effects of race, gender or age.

The second step was to perform global tests to determine if there were any relationship between the explanatory variables and dementia prevalence. These global tests did not involve multiple statistical tests and so corrections for multiple testing were not needed. A bootstrap distribution was created by repeating the above calculation with random permutations of the dementia prevalence across the different geographic areas and years. The first global test (Table 1) involved determining if the *p*-values produced in the above analysis were uniformly distributed on [0, 1] or equal to the bootstrap distribution using the Kolmogov-Smirnov test. In the absence of relationships between any variable an dementia, the *p*-values would be uniformly distributed on [0, 1]. In the second study another global test was performed. The sum of the squares of all of the Spearman correlation or partial correlation coefficients between the explanatory variables and the dementia prevalence variable (∑R^2^) was computed. This was done for the actual data and after random permutations of the dementia prevalence variable (∑R^2^ test). The fraction of the time that the value of ∑R^2^ in the randomized data is greater than or equal to that actually observed is a measure of the likelihood that no association between the explanatory variables and dementia prevalence. These two tests are applied prior to any attempts to identify variables that might be linked to dementia. If the probability that the distribution of *p*-values was uniform (or the probability that random permutations would yield a value of ∑R^2^ greater than or equal to the observed value) was less than 0.1 then subsequent more detailed analysis was justified. If this condition was not met, the data would have been re-reviewed but in the absence of a significant result no further analysis was justified.

In the next phase of the analysis, criteria for selecting explanatory variables that might be associated with the dementia prevalence were considered. One of the important considerations was keeping the probability of a false positive detection (FDR) less than 0.05. In both studies the Benjamini-Hochberg method (Benjamini and Hochberg, 1995) (without the Yekutieli correction) was applied to the *p*-values obtained with each of the univariate correlation measure. A threshold *p*-value was found so that the estimated FDR would be less than 0.05. In the first study, this analysis was carried out using the Pearson *p*-values (due to the limited number of cases). In the second study, this was done with Spearman *p*-values. In each case additional criteria for a significant relationship were used as well. In the original study, this was simply requiring that the Spearman *p*-values be less than 0.05. In the second study, it was also required that the Pearson *p* be less than 0.05 and at least one cross tabulation table and the partial correlation *p*-value must be less than 0.05. In addition variables, were selected only if the probability of any permutation of the dementia prevalence values was associated with a *p*-value less than that measured was less than 0.05 for the Spearman and partial correlation *p*. The effects of the *p*-value used to set the FDR threshold was investigated in Supplementary Tables 1, 2 in simulation studies. In order to understand the effects of considering each time period as a separate case versus considering the average of data from all 8 time periods, another set of analyses were performed in which an addition criterion for variable selection was imposed: that the *p*-value for the Spearman rank correlation be less than 0.05 in the “averaged” data set. Supplementary Table 6 indicates the effect of these criteria on variable selection. The selection criterion based on keeping the FDR < 0.05 was the most stringent but variables were also eliminated by the partial correlation restriction and the restrictions on the “averaged” data set when used.

#### Multiple Linear Regression

Two types of multivariate regression were performed. The first termed exhaustive regression considered a large number of low-order regression models, rank-ordering them by AIC (Akaike Information Criterion). The variables that were in the models with the lowest AIC values were considered to have relationships to the outcome. In the first study this involved considering all models with up to 3 demographic/risk factor variables and 1 food variable. In the second study it involved consideration of all models with up to 3 variables that had been selected in the univariate analysis. In the first study, only the 4 models with the lowest AIC were studied but in the second study all models that were at least as 1% as likely as the best model were studied. Slopes for each variable as well as the variances for the slopes, the *p*-value for each variable and the variance inflation factor (vif) for each variable were determined. A variable was considered to be significant in given model if its *p*-value was sufficiently low (0.05/number variables first study 0.05 s study) along with a vif < 10. High values of the vif indicate that a variable was highly correlated with another variable in the model and contributes little new information.

A second regression technique used in the second study was a forward stepwise regression beginning either with a rank ordered set of the variables considered significant in the univariate selection process or the subset of those variables identified by low order exhaustive regression models.

Simulations carried out in the Supplementary Appendix (Supplementary Tables 1, 2, 12, 19), indicate a high sensitivity of the univariate selection process for finding significant variables but the FDR near 0.05 produced a large number of false positive detections. The exhaustive regression models of the type used in the second study had a very low false positive rate but also a low true positive rate. The forward stepwise regression model had a FDR near 0.002–0.01 with a true positive rate between 0.2 and 0.8 in simulated data.

#### Other Multivariate Analyses

The Supplementary Appendix Sections 1.6.4.4, 1.6.6**, and** 1.6.7 discuss other multivariate techniques such as regression using regularizers, principal components analysis and cluster regression. The regularizer based methods were able to produce a rank order of variables with the strongest relation to the outcome variable but this rank order was sensitive to the arbitrary choice of the value of the regularizer. The clustering methods and principal components analysis created grouping of variables that were not biologically based and so are difficult to interpret, even in simple simulations.

Given the problems with selecting a few variables out of a large number of potential variables, one simple multivariate analysis (Supplementary Appendix 1.6.9) used a weighted sum of standardized food variables (They are naturally in different units so standardizing to mean of zero and standard deviation of 1 was essential.) to create a global food index. One simple and intuitive choice of weights was to set them equal to the value of the partial correlation coefficient. Supplementary Appendix 3.5.1 indicates that this would be the exact solution if the explanatory variables were uncorrelated and completely spanned the space. Variables with low correlation contribute minimally to the new variable. Variables with a positive correlation receive a positive weight and variables with a negative correlation receive a negative weight. Supplementary Appendix Section 1.6.9 shows that a regression analysis using this new variable, age and gender produced a model that provides excellent agreement with the data. In addition, with 200000 simulations of different permutations or random weights, no better fit was obtained with any other combination of the variables. In addition, an iterative process starting with the partial correlation weights to find nearby weights that fit the data better was described in 1.6.9. This resulted in very different coefficients but only minor improvements in the ability to predict dementia prevalence.

### Availability of Data and Materials

As detailed above, all of the source data is publicly available at the listed websites. The datasets used and/or analyzed during the current study are attached as Supplementary Data. All data analysis was carried out using programs written in Mathematica (Wolfram Research Inc., Champaign, IL, United States). These programs are attached in Supplementary Data as well.

## Results

### Global Tests of Association

**FIGURE 1:**
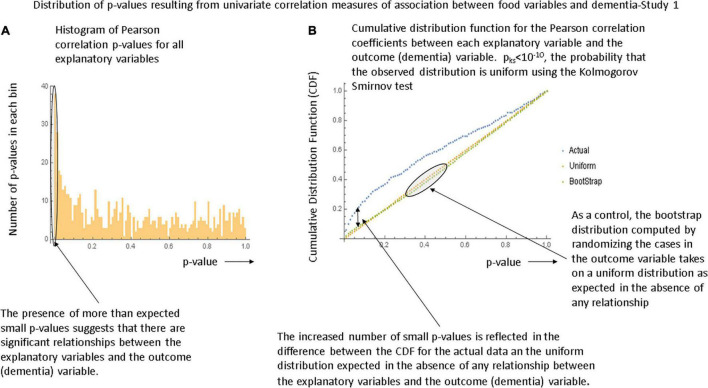
Illustration of the global tests for significance in the first study. **(A)** Histogram of the *p*-values generated by the Pearson correlation tests between each food variable and dementia in the first study. **(B)** The cumulative density function (CDF) describing the *p*-values. The bootstrap distribution is that derived by randomizing the dementia variable values and is linear as expected. The Kolmogorov-Smirnov test demonstrates that the observed distribution is unlikely to be uniform and hence that there is a significant relationship between the explanatory variables and the outcome variable.

**FIGURE 2:**
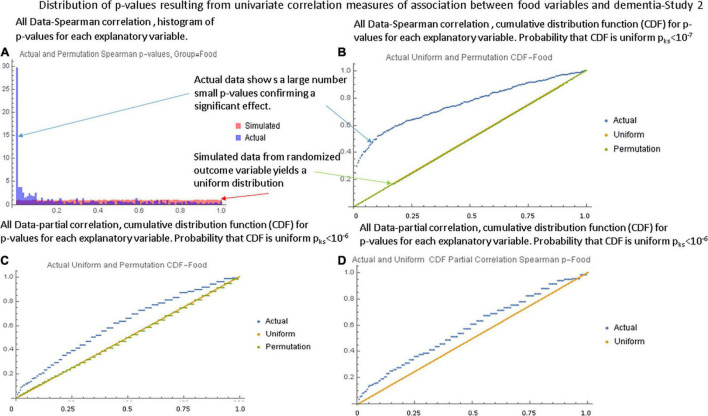
Illustration of the global tests for the second study. **(A)** Histogram of Spearman correlation *p*-values for the food variables with the “full” data set. **(B)** The CDF of the *p*-values showing a strongly non-uniform shape. **(C)** The CDF of Spearman correlation *p*-values in the second study with the “averaged” data set. **(D)** The CDF of the partial correlation *p*-values with the “full” data set. Although the shape is closer to that of a uniform distribution, the Kolmogorov-Smirnov test still shows a large statistical difference.

**FIGURE 3:**
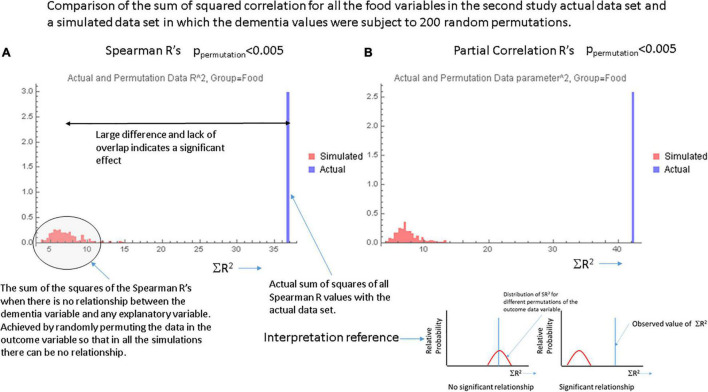
Data from the ∑R^2^ test for global significance with **(A)** the Spearman correlation coefficients and **(B)** the partial correlation coefficients for each food variable. In each case the single bar at the right represents the actual sum of *R*^2^ over all food variables in the actual data set. The smaller bars to the left in each graph show the results of computing the sum of the values of *R*^2^ over 200 random permutations of the data in the dementia variable. These simulations essentially show the values of ∑R^2^ in the absence of any relationship-the null hypothesis. The inset at the lower right shows a cartoon of the findings expected when there is or is not a significant relationship between the explanatory variables and the outcome variable.

Figures 1A,B, respectively show the histogram and cumulative density function (CDF) of *p*-values resulting from applying the Pearson correlation test to measure the relationship between the food variables and dementia prevalence using data from the first study. It is visually clear that they are not uniformly distributed and the Kolmogorov-Smirnov test confirms this. Figures 2A,B show similar plots for the data from the second study this time using the Spearman rank correlation test. Figure 2C shows the CDF of the Spearman rank correlation *p*-values in the second study using only the “averaged” data and finally Figure 2D shows the CDF of the *p*-values associated with the partial correlation of each food variable and dementia in the second study with all data. All of these distributions are significantly different from the uniform distribution that would be expected under the null hypothesis that there was no association. As a control, the permutation/bootstrap distribution *p*-values did have a nearly uniform distribution as expected. Figures 3A,B show that the sum of squares of the correlation coefficients (∑R^2^ method), either partial correlation or raw Spearman R’s, were much larger in the actual data set than in a simulated data set with random permutations of the dementia variable. This confirms, in different data sets, with different metrics, and statistical analyses that there are significant correlations between the data in the food variables and dementia that are not fully explained by the demographic factors. Supplementary Appendix Section 1.6.2 provides additional details.

### Univariate Analyses

After confirmation of a diet effect on dementia prevalence, measures of association between each food variable and dementia prevalence were computed. Supplementary Spreadsheet 3 contains a full list of all univariate statistical tests for each variable colored and rank ordered according to the value of the partial correlation coefficient. The partial correlation coefficient was a better index of the true effect of a dietary factor as it removes the potentially confounding effects of age, gender and race. Table 2 shows 19 variables in each study ranked according to the probability that the association between that variable and dementia could occur by chance. In the first study, the *p*-value from the Pearson correlation was used to rank variables and in the second study the Spearman rank correlation was used. For both studies, the correlation coefficient was shown and for the second study the partial correlations are shown as well. All variables in the table were associated FDR < 0.05 according to the Benjamini-Hochberg algorithm. The only variables that appear on both lists were stroke, race and age. Simulation studies shown in more detail in Supplementary Appendix 1.6.8 indicate that the true false positive rate of the univariate analyses was near 0.05 (even when the FDR threshold was set much lower) so that a large number of false detections might be expected. Thus, in order to get a better insight into the roles of individual factors, multivariate analyses were undertaken.

**TABLE 2:** Univariate relationships between study variables and dementia in the first and second study.

Second Study					First Study			
Variable Name	p_*Sp*_	R_*Sp*_	Partial p_*Sp*_	Partial R_*Sp*_	Variable	R_*P*_	p_*P*_	p_*Sp*_
Fraction 65 + (3)	2.7E-17	0.80	−	−	**% White British Race In Population**	0.99	4.9E-07	0.00024
Other cereal convenience foods (250)	1.6E-13	0.74	8.1E-06	0.50	% Mixed Race in Population	−0.99	1.2E-06	0.0011
Other cereal foods – frozen and not frozen (255)	4.8E-13	0.73	7.2E-09	0.62	% Black Race in Population	−0.98	6.5E-06	0.0037
Other convenience meat products – frozen or not frozen (78)	2.6E-12	0.71	0.00042	0.40	*Dried Rice Consumption*	−0.97	1.2E-05	0.00094
*Cooked rice* (240)	5.6E-12	0.70	6.2E-13	0.72	% Other Race in Population	−0.97	1.7E-05	0.017
Stroke: QOF prevalence (all ages) (14)	4.0E-09	0.63	0.019	0.28	*Total Rice Consumption*	−0.97	1.9E-05	0.0025
Meat based ready meals and convenience meat products (76)	7.4E-09	0.62	0.016	0.28	Death Rate	0.97	2.4E-05	0.0016
Hot dogs and sausage sandwiches (330)	1.9E-08	0.60	1.61E-07	0.57	Birth Rate	−0.97	2.6E-05	0.0016
Fresh and processed potatoes (355)	4.1E-08	0.59	0.0011	0.38	% Asian Race in Population	−0.96	5.5E-05	0.042
Frozen fruit and fruit products (194)	4.5E-08	0.59	5.0E-05	0.46	Mineral or Spring Waters	−0.95	1.2E-04	0.036
Pizzas – frozen and not frozen (248)	7.6E-08	0.58	0.044	0.24	Population density	−0.94	1.6E-04	0.0096
Other soft fruit, fresh (186)	8.4E-08	0.58	0.013	0.29	**Fraction of Population 65+**	0.94	1.9E-04	0.013
Other sponge cakes or desserts (not cream cakes) (477)	2.8E-07	0.56	8.0E-05	0.45	% Other White Race in Population	−0.94	2.0E-04	0.049
Champagne, sparkling wines and wine with mixer (301)	3.9E-07	0.56	2.7E-06	0.52	Prevalence of Hypertension	0.93	2.3E-04	0.04
White British (8)	4.4E-07	0.55	−	−	Bacon and Ham Uncooked	0.93	3.4E-04	0.05
Soft drinks, not concentrated, low calorie (287)	4.5E-07	0.55	0.0031	0.34	Salmon, fresh, chilled or frozen–total	−0.92	5.4E-04	0.0125
Butter and margarine eaten out (429)	4.5E-07	0.55	0.013	0.29	Median Age	0.91	5.6E-04	0.03
Other root vegetables or tubers, e.g., turnip, parsnip, radish, beetroot (373)	1.5E-06	0.53	0.00073	0.39	Other Food And Drink	−0.91	7.1E-04	0.077*
Pizza (247)	5.2E-06	0.51	2.3E-07	0.57	**Stroke Prevalence**	0.91	7.3E-04	0.00391

*19 variables with the lowest p-values are shown. In the first study, ranking was done according to the Pearson correlation probability p_P_. For the second study variables were ranked according to the Spearman rank correlation probability p_Sp_. R_P_ and R_Sp_ are the Pearson and Spearman correlations. In the second study the results from the Spearman correlations after removal of age race and gender information are noted as “Partial.” All results from the second study are in Supplementary Spreadsheets 2, 3. Bolded variable names in the first study column indicated that the variable was also in the first 19 in the second study. The variables for rice consumption are italicized to indicate the presence of cooked rice as significant in the second study and dried rice and total rice in the first. The number in parenthesis next to the first variable name is the number associated with that variable in the main database.*

Plots of the relationship between some of the variables in the above analysis and dementia are shown in Supplementary Figures 11, 12, 18.

### Multivariate Analyses

Multivariate analyses on the data from the first study were limited by the small number of cases and are described further in Supplementary Appendix 2.4 and Supplementary Table 23 where total rice consumption and dried rice consumption were identified as being associated with a reduced dementia prevalence. In the second study, the low order exhaustive regression analysis identified consumption of pure fruit juices and sugar as associated with a lower dementia prevalence while consumption of other cereal foods was associated with an increased risk. The forward stepwise analysis using the full set of data and all variables selected after the univariate analysis identified age as the most important factor in determining dementia prevalence. Also sugar intake, intake of processed fruit and fruit products and other savory or sweet sandwiches were associated with a significantly lower prevalence of dementia. Consumption of champagne, hot dogs and sausage sandwiches, other root vegetables (turnip parsnip radish) were associated with a higher risk. However, the variables selected were different if the criteria for variable selection were changed for example by including only those variables in the best exhaustive regression models. Requiring that a variable be associated with *p* < 0.05 for the Spearman rank correlation analysis in the “averaged” data set also changed the variables selected. These differences are illustrated in Supplementary Tables 10, 11. These tables also demonstrate that although different variables appeared in different multivariate models, many of these variables were strongly correlated. Supplementary Appendix 1.6.5 and Supplementary Figure 14 show that there was a very strong correlation between the variables identified in the univariate analysis. This provides a partial explanation why different variables appear in different multivariate analyses.

**FIGURE 4:**
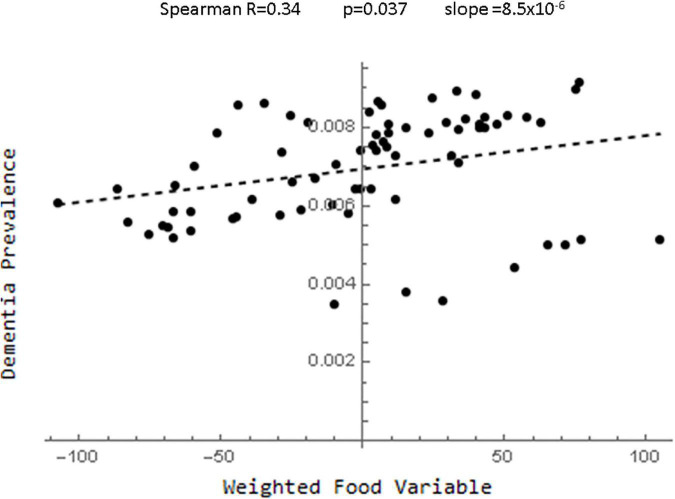
Plot of the relationship between the food index derived by standardizing the food variables and multiplying each by their partial correlation coefficients and dementia.

One method of avoiding the problem of choosing between highly correlated variables, was to instead create one index by weighting all of the dietary data (Supplementary Appendix 1.6.9). To see how well this worked, each food variable was standardized and a new variable was created by multiplying each standardized variable by the value of the partial correlation. This global food index variable along with age race and gender entered into a forward stepwise regression analysis with *p* < 0.001 and low variance (Supplementary Table 20). Figure 4 (and Supplementary Figure 16) showed that there was a strong linear correlation between this food index and dementia prevalence. However, this correlation was not greater that of all of the individual food variables and as documented in the Supplementary Appendix many other weightings can be created with even better fits to the data.

### Relations to Other Studies

It is useful to understand the relationship between the findings in this paper and those of other studies. Table 3 shows the broad dietary categories used in creating the DASH, Mediterranean (MeDi) and MIND diet indices (Morris et al., 2015) along with the corresponding dietary elements from the second study using the partial correlation as the index of relationship to dementia prevalence. Two things are clear. First, the predicted effect of consuming different amounts of food in some of the broad categories used in previous studies on dementia prevalence was very different from that predicted from the current study. Second, many different food variables in the current study fell under the broad categories used in the previous studies. In those cases, Table 3 demonstrates, that different food variables within a given dietary category had very different relationships to dementia prevalence.

**TABLE 3:** Comparison of index elements in the DASH, Mediterranean and MIND diets (Morris et al., 2015) that are also in the current study.

	Prior study indices			Current target group		Current study group extremes			
			
Index element	DASH	MeDi	MIND	R Mean	R Std	R Min	Minimum element	R Max	Maximum element
Grains	−	−	−	–0.12	0.28	–0.65	Bread	0.72	Cooked rice
Vegetables	−	−		0.031	0.22	–0.51	Fresh Green Vegetables	0.39	Other root vegetables
Green Leafy Vegetables			−	–0.44	0.076	–0.49	Lettuce and Leafy Salads	–0.39	Leafy Salad Fresh
Potatoes		−		–0.074	0.28	–0.53	Potatoes	0.43	Potatoes-Mashed
Fruits	−	−		–0.10	0.34	–0.73	Pure fruit Juice	0.46	Frozen Fruit and fruit products
Dairy	−	+		–0.11	0.29	–0.60	Hard Cheese Cheddar type	0.43	Soft drinks including Milk
Red meats		+	+	–0.08	0.19	–0.30	Beef Steak-More Expensive	0.22	Corned Meat
Fish		−	−	0.078	0.16	–0.18	White Fish Frozen	0.38	Other tinned or bottled fish
Poultry		−	−	0.10	0.23	–0.27	Other poultry	0.52	Chicken burger
Nuts, seeds, and legumes	−	−		0.065	0.075	–0.046	Nuts and Crisps	0.11	Nuts edible seeds and peanut butter
Beans			−	–0.18	0.32	–0.50	Beans, fresh	0.19	Other canned beans and pulses
Nuts			−	0.065	0.075	–0.046	Nuts and crisps	0.11	Nuts edible seeds and peanut butter
Total Fat	+			–0.19	0.26	–0.60	All other fats	0.09	Fats, preserves, sugar and custard
Olive oil		−	−	–0.4					
Butter margarine			+	0.11	0.26	–0.08	Butter	0.291	Butter and margarine
Cheese			+	–0.026	0.30	–0.60	Hard cheese cheddar type	0.37	Cheese and egg dishes or pizza
Sweets	+			–0.085	0.28	–0.40	Chocolate coated bars and sweets	0.33	Boiled sweets
Pastries sweets			+	–0.30	0.27	–0.52	Cakes and pastries not frozen	0.17	Take away pastries
Sodium	+			–0.49					
Alcohol		+	+	0.038	0.31	–0.40	Fortified wines	0.52	Champagne, sparkling wines and wine with mixer

*In columns 2, 3, 4, a+ sign indicates that consuming more of the given food changes the index in such a way as to increase the risk of dementia. A – sign indicates the opposite. There are often many entries in the family food database that would be associated with the index element indicated in each diet. The values listed under current target group are the mean partial correlation coefficient and its standard deviation over all food elements that fit the Index element in the current study. To the right of this is the food variable in the group with the smallest R and the food variable with the largest R. If the results from Morris et al. (2015) and the current study were in agreement the signs of the R-value would be the same as that of the index for a given diet.*

## Discussion

The most important conclusion from the analyses presented above was the significant effect of dietary factors on the prevalence of dementia which was not explained by demographic factors. This was seen in both studies and with different statistical procedures and so is a reliable conclusion.

### Statistical Considerations

Looking more deeply into the data, the univariate analyses were able to identify some dietary factors that were more likely than others to be associated with dementia prevalence and could be the starting point for future studies. However, the problem of identifying a single dietary factor or cluster of factors with certainty quickly ran into the limitations imposed by the small number of cases and the large number of highly correlated variables. Even in the simplest univariate analyses, the list of dietary factors that are significant are slightly different in the two studies and the list of most significant factors varies with the specific univariate analysis chosen. Simulations showed that the univariate testing was associated with a high false positive rate (even with lower FDR thresholds). The multivariable analyses, especially the exhaustive regression and the forward stepwise regression, lower the false positive detection rate significantly, but because of the high degree of correlation between the various food variables different multivariate models select different variables. The simplest multivariable model, creating a single index by weighting the food variables was associated with problems. The *a priori* choice of the partial correlation R values as weights created a new variable that did correlate with dementia but not better than some of the individual variables. In addition, simulation studies demonstrated that very different choices of weights produced a global food variable with a similar degree of concordance with dementia. Thus, the food variables could be used to predict dementia prevalence but determining the effects of individual variables was problematic. It is important to note that blindly following statistical algorithms would have produced a variable with a significant correlation. Only by the use of simulations and multiple methods of analysis was it possible to know that the construction of such a variable could not predict the effects of individual variables. Similar effects were noted when univariate methods were used to select smaller groups of variables for multivariable analysis or when automated classification schemes based on clustering or principal components were used.

All of these problems are expected when the number of variables was much lower than the number of cases so that there must be multiple solutions to the prediction problem. In this context, it was important to recognize for this and similar studies that the ability to predict an outcome variable may be much easier than determining the variables that contribute most to the outcome. These issues can be resolved only with more data and/or better methods for constructing *a priori* groupings of the food variables. Even with this limitation, this study showed the relative value of different analysis methods and quantified them with simulation studies. It also demonstrated how different data collection and analysis methods all lead to the conclusion that there is an effect of diet although they differ in predicting the most important variables. Understanding these issues is an important step in toward designing and optimizing future studies.

Despite these limitations, it is important to note that a number of variables such as those in Table 2, particularly the consumption of rice, cereals, alcohol, fruit, processed potato products and certain meat products were highlighted as potential candidates for future studies.

### Relationships Between Diet and Dementia

Despite the statistical limitations, the results of this pilot study can be useful in generating preliminary hypotheses for a more detailed search for relationships between food intake and dementia. First among these was illustrated in Table 3. It was clear that many of the dietary categories used to assess the DASH, MeDi and MIND diets are too broad. They include specific dietary elements that might have very different relationships with dementia prevalence. Consider the effect of red meats. Both the MeDi and MIND diet indices suggest that increasing consumption of red meat is associated with an increased risk of dementia but in the current study the consumption of “beef steak-more expensive” was associated with a lower risk while consumption of “corned meats” or “hot dogs and sausage sandwiches” was associated with a higher risk. The consumption of “beef steak-less expensive” had a low association with dementia prevalence. This illustrates the problem with studying very broad dietary categories. If there were heterogeneity within a category and different populations consume different relative proportions of different foods in the category, there could be substantial unexplained variations in the parameter reducing the chance of finding a strong relationship to dementia. For example, if a large fraction of one population consumed more expensive beef steak and very few hot dogs and another consumed little steak and a large amount of hot dogs the index “red meat” might be the same yet the relation to dementia would be very different. This would reduce the possibility of finding statistically significant results. The recent study from Zhang et al. (2021) supports the findings of this study by noting that increasing intake of unprocessed meat was associated with a decreased risk of dementia while the opposite was true for processed meat.

Because the consumption of rice had conflicting effects in some of the models, it was useful to discuss the relationship between rice and dementia in more detail. Although in one meta-analysis, Hu did find (Hu et al., 2012) that higher consumption of white rice was associated with a significant increase in the risk of type 2 diabetes, a newer study (Shi et al., 2017) and a more recent meta-analysis (Saneei et al., 2017) found only weak evidence that white rice consumption modified mortality and did not find convincing evidence of white rice intake on specific illnesses such as obesity, hypertension, diabetes or cancer. Despite this, there was some evidence that substituting brown rice for white rice (Sun et al., 2010; Malik et al., 2019) might be beneficial at lowering hemoglobin A1c and lowering the risk of diabetes. It has also been proposed that brown rice consumption is associated with lower risk of insulin resistance (Choi et al., 2014). There is little work on any possible relationship between dementia and rice intake. Some experimental data suggests that brown rice might improve cognitive function and reduce b-amyloid in the brains of an Alzheimer’s mouse model (Okuda et al., 2018) and that rice bran extract improves mitochondrial function in mice (Hagl et al., 2016). On the other hand, some clinical data (Ozawa et al., 2013) suggests that increased rice intake might be associated with a higher risk of dementia while others (Grant, 2016) suggest that a diet high in grains might be associated with a lower prevalence of dementia. Although the United Kingdom family food database did not provide information regarding the differences between white and brown rice, it did provide information on total rice consumption, cooked rice and dried rice. Although dried rice and total rice consumption were associated with lower values of dementia prevalence with Spearman R′s of −0.26 and −0.39 respectively, the partial correlation coefficients were 0.22 and 0.008 respectively. This large difference suggests that a great deal of the effects of dry and total rice consumption were due to a correlation with demographic factors. Consumption of cooked rice was associated with an increased risk of dementia with a raw Spearman R of 0.70 as well as a partial correlation coefficient of 0.72 so that its effect could not be explained by the demographic factors in the model. It was not highly correlated with either total rice or dried rice consumption but was highly negatively correlated with sugar intake and positively correlated with cereal intake and convenience meat intake. This could indicate that the effect of cooked rice consumption was related to another dietary intake or behavior that covaries with cooked rice consumption.

Further illustrating the complexity of the issues surrounding dietary effects on dementia was a study by Takeuchi and Kawashima (2021) that showed non-linear effects of certain dietary intakes on dementia risk. For example, moderate consumptions of meat were associated with lower risks of dementia while the highest intakes of cheese and bread were associated with the lowest risks of dementia. These findings are consistent with the findings in this study but the effects of meat and cheese are different than what would be predicted from the effect of the DASH, Mediterranean or MIND diets. Takeuchi and Kawashima (2021) also found a linear increase in risk for dementia with increased vegetable and fruit intake which is different than what the studies of the MIND, MeDi and DASH diets have found (Morris et al., 2015). In light of the above discussion an alternative explanation was that, as intake of vegetables increases, the mixture of different vegetables changes from fresh green vegetables that are associated with a lower risk of dementia to other root vegetables that are associated with a higher risk of dementia (Table 3).

### Conclusion

The above discussion illustrates a number of major problems that can arise in studies of the relationship between dietary factors and dementia. First, quantifying a diet is very complex. Simply specifying a diet in terms of high level nutritional categories such as grains, fats, anti-oxidants, or meats does not capture all of the useful information and in fact can obscure important information when different nutrients within a broad category have different effects as shown above. Second, the necessity of capturing a large amount of information about diet implies that large studies would be needed to identify specific foods associated with dementia prevalence. One way to approach this is to begin with a set of preliminary studies to classify variables into groups and then once studies have agreed on a data driven classification scheme, perform larger scale studies using those groups. Third, in studies such as these with a large number of highly correlated explanatory variables, any statistical testing must be used with caution. The advantages and disadvantages associated with each of the statistical and methodological approaches should be carefully studied with simulation studies and results accepted as accurate only if multiple methods show the same result.

## Data Availability Statement

The original contributions presented in the study are included in the article/Supplementary Material, further inquiries can be directed to the corresponding author.

## Ethics Statement

Ethical review and approval was not required for the study on human participants in accordance with the local legislation and institutional requirements. Written informed consent for participation was not required for this study in accordance with the national legislation and the institutional requirements.

## Author Contributions

MaS, MoS, AR, and LK contributed to the design of the study and to editing/reviewing the manuscript as well as the literature review. MaS performed the statistical analysis and produced the original manuscript drafts. All authors contributed to the article and approved the submitted version.

## Conflict of Interest

The authors declare that the research was conducted in the absence of any commercial or financial relationships that could be construed as a potential conflict of interest.

## Publisher’s Note

All claims expressed in this article are solely those of the authors and do not necessarily represent those of their affiliated organizations, or those of the publisher, the editors and the reviewers. Any product that may be evaluated in this article, or claim that may be made by its manufacturer, is not guaranteed or endorsed by the publisher.

## Footnotes

1
https://fingertips.phe.org.uk

2
https://www.nationalarchives.gov.uk/doc/open-government-licence/version/3/

3
https://www.gov.uk/government/statistical-data-sets/family-food-datasets

## Supplementary Material

All of the files can be found in the zip file Data Sheet 1.zip.
