# Supplementary material for: Dementia and Diet, Methodological and Statistical Issues: A Pilot Study
Source: Front Aging Neurosci. 2022 Jul 6;14:606424. doi: 10.3389/fnagi.2022.606424 (PMC9298542; doi:10.3389/fnagi.2022.606424)
Supplement: Supplementary file 1 [file Data_Sheet_1.zip › SupplementaryAppendix.docx]

**Appendix-Methodology and Statistical Considerations**

**0.0-Introduction**

**1.0-Revised (Second Study) Methodology**

**1.1-Geographic Regions**

**1.2-Time Periods**

**1.3-Clinical and Demographic Data**

*1.3.1-Dementia Prevalence*

*1.3.2-Medical Comorbidities*

*1.3.3-Population Characteristics*

*1.3.4-Family Food*

**1.4-Variable Selection**

**1.5-Initial Data Processing**

**1.6-Data Analysis**

*1.6.1-Initial Simulations*

*1.6.2-Global tests for significance*

*1.6.3-Univariate Analysis*

*1.6.3.1-* Univariate Tests *1.6.3.2-*Selection Criteria

*1.6.3.3-*Univariate Results

*1.6.4-Multivariate Analyses*

*1.6.4.1-*Low Order Exhaustive Regression

*1.6.4.2*-Forward Stepwise Regression

*1.6.4.3*-Forward Stepwise Regression with Noise

*1.6.4.4*-Regularized Regression

*1.6.5-Correlations*

*1.6.6-Cluster Analysis*

*1.6.7-Principle Components Analysis*

*1.6.8-Post Analysis Simulations*

*1.6.9 Construction of a Single Index From the Food Variable*

*1.6.10-Connections between the first and second studies*

*1.6.11-Comparisons to Other Studies*

**1.7-Summary/The Future**

**2.0-Original (First Study) Methodology**

**2.1 Data Sources**

**2.2 Statistical Analysis**

*2.2.1 Correlation (Univariate) Analysis*

*2.2.2 Multiple Linear Regression*

*2.2.3 Principal Components Analysis*

*2.2.4 Variable reduction techniques*

**2.3 Availability of Data and Materials**

**2.4 Results**

*2.4.1 UK data*

**3.0-Statistical Analyses**

**3.1-Introduction**

**3.2-Effects on Outcome and Predictive Modelling**

3.2.1-A Simple Model

3.2.2-A More Complex Situation with Simulation

**3.3-General Model Formulation**

**3.4-Reformulations of the Linear Model**

3.4.1-Introduction

3.4.2-The Least Squares Formulation

**3.5-Residuals and Measures of Influence**

3.5.1-Transformation and normalization of the problem

3.5.2-A Special Case

**3.6—Solutions**

3.6.1-Clustering

3.6.2-Regularizers

**4.0-A Short Illustrated Tutorial on the Distribution of p-values**

**5.0-Tables**

**6.0-Figure Legends**

**7.0-Figures**

**8.0-Supplemental Spreadsheet Legends**

**9.0-Computer Programs**

**10.0-References**

**0.0 Introduction**

The goal of this study is to find, among a large number of possible predictors derived from the UK Family Food Study, variables that best predict the prevalence of dementia in the nine regions of England. Studies of this type can be helpful in creating hypotheses and designing studies to test them. However, the problems associated with the methodology can be very significant and so it is important to discuss them in detail. This appendix is divided into three main sections. The first describes in detail the approach to the UK Family Food data that was used in the second study. It highlights the strengths and weaknesses of the methodology using simulation studies that were not available at the initial submission. In addition, the use of many different multivariable analyses will provide insight into the developing optimal approaches to data analysis. Also, since the initial study, more data has become available and it was useful to analyze that data and compare results with that of the initial study.

The second part of this appendix contains additional information on how the original study was carried out as it is very important to be able to compare the results obtained in two related studies that use slightly different methodology. This allows some idea about the reliability of the results.

The third part of the appendix discusses the statistical methodology in more detail including some ideas that were incorporated into the revised methodology and some that were not yet included. Understanding the limitations of this study from multiple standpoints is important in designing future studies that follow up on the hypotheses generated in this pilot study.

**1.0 Revised Methodology**

In comparison with the methodology used in the analysis in the first study, this approach concentrates on the 9 regions of England rather than the entire UK and hence allowed more intensive analysis of the existing data. It also allowed an analysis of data from different points in time. Two critical issues in the data collection are the geographic regions over which the data is tabulated and the time periods represented by each data element.

**1.1 Geographic Regions**

Health and demographic data in the UK at different geographic levels. The top level of these geographic classifications are the 9 regions of England officially designated as the NUTS1 (Nomenclature of Territorial Units for Statistics) statistical regions (https://www.lgbce.org.uk/). They are: East of England, East Midlands, London, North East, North West, South East, South West, West Midlands, and Yorkshire and the Humber. These are critical to this study as the data from UK Family Food is only publicly available at this level.

At the next level are the counties (https://ec.europa.eu/eurostat/documents/345175/7451602/nuts-map-UK.pdf). Although some clinical data is available at this level, more clinical data from the National Health Service (NHS) is published for each CCG or Clinical Commissioning Group (https://www.england.nhs.uk/ccgs/) which is a geographic unit used by the NHS to organize regional healthcare services. Mappings between the these geographic subunits and the Regions of England were done using files downloadable from the Office for National Statistics (ONS-https://geoportal.statistics.gov.uk/search?collection=Dataset&sort=name&tags=all(PRD_IPN)). When data was available at multiple levels results were checked to make sure that similar results were obtained using each geographic subunit.

**1.2 Time Periods**

Data was available for a number of different time periods. The value of some data elements is tabulated over a calendar year from January to January and other data elements tabulated from the middle of one year to the middle of the next year. For example, the family food data from 2003-2006 was on a mid-year basis then on 2006-2015 on a January to January basis and then after 2015 on a mid-year basis. All variables obtained at different times were mapped to the time intervals used by the family food data using a simple average. If a data element had a given value in for example 2013 and 2014 its value for the mid-year 2013-2014 was taken as the average of these two values.

**1.3 Clinical and Demographic Data**

As stated on the relevant websites access to the data used in this study was governed by the Crown copyright which allowed free-reuse of the data on these websites as long as the following citation is present (Public Health England. Public Health Profiles. 12/05/2021 https://fingertips.phe.org.uk © Crown copyright 2021). In addition is recommended that the following statement be included as well: the data is public sector data licensed under the Open Government License v3.0 (https://www.nationalarchives.gov.uk/doc/open-government-licence/version/3/).

*1.3.1 Dementia Prevalence*

Data was downloaded from the Dementia Profile page (https://fingertips.phe.org.uk/profile-group/mental-health/profile/dementia/data#page/0) using the CCG geography. An additional download of data using the Counties&UAs geography was also performed and the analysis confirmed that the same results for each region were obtained.

There are a number of different indicators but the primary one used in this study is titled “Dementia prevalence QOF” (QOF stands for quality outcome framework). This is defined as: “The recorded dementia prevalence is the number of people with dementia recorded on GP practice registers as a proportion of the people (all ages) registered at each GP practice. “ (https://fingertips.phe.org.uk/profile-group/mental-health/profile/dementia/data#page/6/gid/1938132811/pat/15/par/E92000001/ati/166/are/E38000004/iid/247/age/1/sex/4/cat/-1/ctp/-1/yrr/1/cid/4/tbm/1). A secondary measurement is “Dementia: Recorded prevalence (aged 65 years and over)” which is defined as “The percentage of patients (aged 65+) with dementia as recorded on all open and active GP practice disease registers.”. This index is not used in the current study as it is available for fewer time periods.

*1.3.2 Medical Comorbidities*

The public health profiles page (https://fingertips.phe.org.uk/) provides access to a number of different profiles that contain risk factors for dementia. These included: “cardiovascular disease, diabetes and kidney disease”, “musculoskeletal conditions”, “mental health dementia and neurology”, and ”local authority health profiles”. This group contained a large number of indicators that were selected for inclusion as below.

*1.3.3 Population Characteristics*

The racial composition of each region is taken from the 2011 UK census data (https://www.ethnicity-facts-figures.service.gov.uk/uk-population-by-ethnicity/national-and-regional-populations/regional-ethnic-diversity/latest#areas-of-england-and-wales-by-ethnicity). As this is not updated regularly, the same values were used for each year.

Information regarding the age and sex of population of each region was available from the Office of National Statistics (ONS) (https://www.ons.gov.uk/peoplepopulationandcommunity/populationandmigration/populationestimates/datasets/populationestimatesforukenglandandwalesscotlandandnorthernireland)which is also available under the Open Government License v3.0.

*1.3.4 Family Food*

The family food data is downloaded from https://www.gov.uk/government/statistical-data-sets/family-food-datasets specifically the files CR-household purchases, CR-eating out purchases, CR-household nutrient intakes and CR-eating out nutrient intakes. These files had the data broken out by counties and regions (CR) for the years 2001-2019. All of this data is also available under the Open Government License v3.0. The sum of the nutrient intakes from both eating out and household consumption was computed.

It is important to note that the family food database was based on food purchased rather than food consumed. This allowed verification of the data against purchase receipts. Although this is not equivalent to food consumed, a direct verified measurement of food consumed would be more demanding.

**1.4 Variable Selection**

The determination of which variables were included in the final analysis was made before the analysis was performed. The only years selected for inclusion in the database were those in which a valid value of Dementia-QOF was available for each region (years: 2011, 2012, 2013,2014,2015,2015-2016,2017-2018,2018-2019). The data file used of analysis included only those data elements that had complete data for each of these years and each region. In this file the demographic and risk factor variables were: fraction of the population that was female, the fraction of the population 65 years and over, the population density, the fraction of population that were “Asian”, “Black”, “Mixed”, “White British” or “Other” (These were terms used by the census and were not created for this study.). In addition, Dementia: QOF prevalence, CHD: QOF prevalence (CHD=Chronic Heart Disease), CKD: QOF prevalence (CKD=Chronic Kidney Disease), and Stroke: QOF prevalence were included. There were 507 dietary variables.

**1.5 Initial Data Processing**

After one of the files containing medical data is downloaded from the public health profiles page (https://fingertips.phe.org.uk/) the Mathematica (Wolfram Research, Champaign IL) program trecoder*.nb is run on that file. This program reads a list of associations between CCG’s and the nine regions of England as well as a list of associations between counties and the nine regions of England. Only those indicators and dates are used where there is information on the number of patients described by the indicator and the total population in each geographic area. This allowed computation of the fraction of people meeting each indicator condition in each region. An .xlsx file is created from the raw data for the next phase of the analysis. The family food file is processed by a program called redoer.nb to create a single label for each data element and to put the data in the same format as the other files. Finally another program melder*.nb is used to combine each of the data files into a single file in which the data from each individual file is combined so that a final comprehensive data file was generated for analysis. For reference this spreadsheet is included as supplemental spreadsheet S1. It is important to note that the full variable names in the family food database have multiple headers and the variable names are approximate because of this. The first column of Supplementary spreadsheet S3 has a better list of the variable names that comes directly from some of the source files while the second column has the longer headers in the raw data files. Note that because of some labelling differences between different source files the nutrients folic acid and protein are not in the final spreadsheet.

Variables were deleted if they contained non-numerical data in any cell. They were also deleted if there was a 0 entry in any cell (indicating that there may not be good enough sampling to make that variable reliable) or if all entries were the same.

In summary the family food data comes from 4 files: 1)one that refers to intakes in the house, 2)another that refers to dietary intakes when eating out , 3)another that refers to overall nutrients consumed when eating at home and 4)a file that refers to nutrients consumed eating out. The first file had 336 variables (293 had non-zero data in all cells) , the second had 315 variables (182 had non-zero data in all cells), the third had 63 variables and the last had 61 variables. For the last two files, the total nutrients were added to come up with total nutrients (eating in and eating out combined). Only variable names that were perfect matches were included and any variable with a zero in any cell or had identical data in all cells were deleted. This left a total of 32 variables from the nutrient files. Thus were a total of 507 food variables included. There were 12 risk/demographic factors for a total of 519 variables in the main data file.

Tables S30 shows summary statistics for the risk/demographic factors and Table S31 shows summary statistics for some of the food variables that appear in the analyses.

**1.6 Data Analysis**

The first step in the analysis was to understand that there are multiple ways to use the data from different time periods. It would be ideal to have data over a long period of time so that exposure to different foods over a long period of time could be studied in detail since it is expected that the effect of any exposure may take an extended period to have any effect on dementia. There was not enough data to undertake this type of analysis and so for this pilot study, there were two major options: including the data from each time point and region as a separate case or averaging the data over the time periods so that only each region was a separate case.

Including data from each time point and region as a separate case allows maximal use of the data but, if the data from the different time periods is highly correlated, then this procedure falsely increases the significance of any statistical relationships. On the other hand only using the average of the data averaged over all the time periods may exclude useful information from the different time periods and reduce the significance of the results. Our final approach was to utilize all of the data but perform some tests on the averaged data only and to allow for statistical testing based on the averaged data as well as the full set of data

Figures S1-S6 outline the data analysis approach. Mathematica program mainanalysis.nb can perform the simulations and the analysis. The mathematica program explorer2.nb is used to find the correlations between independent variables.

*1.6.1 Initial Simulations*

In order to determine optimal parameters and get a basic understanding of the analysis before looking at the data, simulations were run. The same data was used as in the actual analysis except that the dependent variable Dementia QOF was replaced by a linear combination of other variables. To this is added normally distributed random noise with a mean of zero and a standard deviation which is a fraction (frax) of the standard deviation of the linear combination of independent variables. Thus, when frax=1 the “noise” and the signal have similar variances while when frax=0.1, the noise is 10 times smaller than the signal. The time to run the analysis program was long so the number of simulations was small. 20 simulations were performed in each condition. Only 3 permutations were considered in the univariate permutation tests for significance and the exhaustive regression was only up to order 2. In the simulations leading to Table S1, the noise level (frax) and the criterion for selecting variables for the univariate analyses were varied. There are three outcome levels for the analysis: selection as significant in the univariate analysis, selected as significant in a backward stepwise multiple regression analysis and selected as significant in a forward stepwise regression analysis. Both of the multiple regression analyses use the variables determined as significant in the univariate analysis. Results are shown in table S1.

There are a number of observations that are important at this point. First, the backward stepwise regression is not helpful at finding the best variable, this is mainly because the target variable is often eliminated during one of the steps while maintaining a good fit due to the correlations between multiple variables. Second, the false positive rate is not strongly affected by the cutoff selected for the p-value in the Spearman rank correlation test. This relates to the fact that there are strong correlations between independent variables and so there are many variables highly associated with the outcome variable. This means that the associations between the independent variables and the outcome variable are not random. Third, the forward stepwise procedure has a good chance of selecting the correct variable with a much lower false positive rate than the univariate selection process. This is especially true because the first variable to be considered in the stepwise process is the one with the highest significance level in the univariate testing.

In order to see how accurate the variable selection process is when multiple independent variables contribute to the outcome an additional set of simulations was undertaken. The outcome variable was taken as the sum of the values of variables 103, 203,303,403 and 503 each normalized by their variance so that they made an equal contribution to the dependent variable. As above, random noise is added to the outcome variable before analysis and 20 simulations are undertaken. Results are shown in table S2.

These simulations suggest that as the number of variables associated with the outcome increased from 1 to 5 the ability to detect relationships is reduced as would be expected. In addition, variable 203 is never selected in the univariate analysis while the others are detected to varying degrees. This is because variable 203 has a strong negative correlation with the value in variable 403 so that the Spearman rank correlation R value for 203 with the outcome variable is only 0.03 although it would have been much larger close to 1 if 203 was the only variable determining the outcome variable. As before, the selection of significant variables using the forward stepwise multiple regression analysis after variables are selected by the univariate analysis and then ordered by the exhaustive regression analysis seems to have a relatively good true positive and false positive rate. Overall the balance between true positives and false positives is much better with the forward stepwise analysis than with the univariate analysis alone and so this is a critical step.

We performed these simulation studies early in the data analysis so we knew which techniques to study and settings to choose prior to the analysis of the actual data. In a later section, the cluster method will be discussed as will be the simulations involved in its development.

*1.6.2 Global tests for significance*

At the top level of the data analysis, it was important to know whether there was any relationship between food intake and dementia before proceeding with any further analysis. To this point, three groups of variables were created. One included all variables, one included only demographic and medical risk factor data and the third included only the diet variables. Univariate measures of association between each variable and the dementia (outcome) variable were computed. Ideally if there were no relationship between the independent variables and the dependent variable then the p-values associated with these tests would be uniformly distributed on the interval [0,1]. This hypothesis was tested using both the Kolmogorov-Smirnov and Anderson-Darling tests in order to be sure that any findings were not test dependent. The same univariate testing was performed after 200 random permutations of the dependent variable values and the distribution of p-values from these univariate association tests re-computed. The Kolmogorov-Smirnov and Anderson-Darling tests were applied to determine whether the actual distribution of p-values was the same as that in the permutation tests. Testing was performed for each group to determine whether there were significant differences which would suggest that the independent variables in that group did bear a relationship to the dementia outcome variable. Table S3 shows the results of this testing. They demonstrate that the actual distribution of p-values from the Spearman rank correlation was neither uniform nor the same as that with the permuted values of the dependent variable for every group. This is also true whether the full set of data is used or the data averaged over the different time periods. This top-level test indicates that there are statistically significant effects of the food variables on the dementia prevalence measure and justifies further analysis.

Figure S7 shows the distribution of p-values (a,b,c) as well as the cumulative density function (CDF)(d,e,f) for the p-values from the Spearman rank correlation for each group. It is clear that in each group the measured p-values from the Spearman rank test are clustered around low values but the values obtained after permutation are much more uniformly distributed.

One of the key emphases of the current analysis was to make sure that any significant findings were as independent as possible of the actual test employed. Thus, another approach was also used. A parameter (called R2) was created which was the sum of the square of all the test statistics from the Spearman rank correlations (R2) of all the variables in a group with dementia. Larger values of the parameter indicate stronger associations between the dependent variable and the group of independent variables. This parameter was re-calculated for all of the permutations of the dependent variable and the probability that with one of the permutations a parameter value greater than that for the actual data is computed. Table S4a contains the results of this analysis. In addition, this analysis was applied to the R values obtained in the partial correlation analysis (Table S4b-after the effects of population age, race and gender are removed) for the full data set and none of the R2 values exceeded that measured in any group providing additional evidence for a significant relationship between the food variables and dementia that was not mediated by the demographic factors. However, using the averaged data there was no significant difference. This is as expected as in the averaged set there only 9 cases while there were 3 covariates.

Figure S8 demonstrates histograms of the values of R2 computed with the permutations of the dependent variable and with that seen with the actual data. As suggested by the analysis of the p-values, the observed degree of correlation between the variables in each group and dementia is much greater than would be expected by chance. Figure S9 shows the cumulative density function for the univariate p values when only the averaged data is used. This method shows that there remains a significant difference (Table S3) from what would be expected if there was no relationship.

The R2 statistic was also computed from the partial correlation Spearman R values after the linear effects of the covariates (age, gender, and race) are removed from both the dependent and independent variables Figure S8 (d). Figure S10 shows a smaller degree of difference between the measured value and the results of the various permutations in the dependent variable than with the raw R values when using the averaged data but there is still a significant difference for all variables and for the demographic variables and a trend for the food variables even in this case. When the full data set was used none of the sum of R2 exceeded that in the actual data set even in the food group only. This provides strong evidence of an effect of food variables after the effects of the covariates have been removed.

*1.6.3 Univariate Analysis*

*1.6.3.1* Univariate tests

The above tests provide evidence for a relationship which justified further more detailed analysis of the data. The first step in this regard was computing multiple tests of univariate association between the independent variables including:

1-Spearman rank correlation test

2-Pearson correlation test

3-Goodman-Kruskal test

4-Hoeffding test

5-Blomqvst test

6-Single Variable Linear Regression. A constant is included in each analysis but is not displayed.

7-Partial correlation coefficient (after the effects of population age, race and gender are removed). These covariate data are removed from both the dependent and the independent variable and Spearman rank correlation between the residuals is computed yielding a p-value and a correlation coefficient R.

8-2x2 cross tabulation table p-value. These cross tabulation tables were generated by dividing both the independent variable and the dependent variable data into quantiles. Each data point was assigned to a table entry based on the quantile for each dependent variable and that of its corresponding independent variable. R2 analysis was used to determine the probability that the resultant table would be obtained if there was no relationship. In addition an exact binomial test was used to assess the probability that the total number of cases lying on the main diagonal (or anti-diagonal if there is a negative correlation) would be seen by chance. This second measure was mainly confirmatory.

9-3x3 cross tabulation table p-value

10-4x4 cross tabulation table p-value

11-Permutation tests for each univariate measure using 200 random permutations of the dependent variable (Dementia QOF).

Again, multiple methods were used to ensure that any results were not method specific. Table S5 gives an idea on the degree of correlation between the p-values generated by these tests.

Figure S11 shows univariate plots of dementia prevalence versus some of the independent variables that are significant in the analyses using the full set of data. Figure S12 shows some of these graphs using only the averaged data set.

*1.6.3.2* Selection Criteria

The main problem with performing a large number of univariate analyses is accounting for the effect of multiple testing especially in highly correlated independent variables. This was done by applying some selection criteria:

1-False discovery rate (FDR) for the p-values resulting from the Spearman rank correlation test quantitating the relationship between each variable and the outcome variable less than 0.05. The Spearman p-value associated with an FDR<0.05 was computed by ordering all of the p-values obtained and applying the Benjamini-Hochberg algorithm. The program offers the option of selecting other choices but this was used in all analyses. This criterion eliminates the largest number of variables (Table S6).

2-Probability that the p-value obtained in the permutations from the Spearman rank test is less than that obtained from the actual data is less than 0.05 (p<0.05).

3- The p value associated with the Pearson test must be less than 0.05 (p<0.05).

4-The probability that at least one of the cross tabulation tables would occur at random is less than 0.05 (pmin<0.05).

5-The p-value associated with the partial correlation is <0.05 (for the non-covariates). The covariates are automatically defined as meeting this criteria (p<0.05).

6- Probability that the p-value obtained in the permutations of the dependent variable produce a p-value less than that of the actual value is less than 0.05 (p<0.05).

There are three options on how to use the “averaged data”. Option one is to just analyze the full data set. The second option is to just use the “averaged data set” (with no restrictions on the significance of the partial correlation as there are very few cases. The third option is to add on restriction to the variable selection process noted above based on the data in the averaged data set:

7-p<0.05 for the Spearman rank correlation in the averaged data

Table S6 shows the number of variables in the model before and after each selection criterion. As expected the first criterion is most stringent in selecting variables. Criteria 2,3,4 have very little effect. The requirement of a significant partial correlation eliminates some variables as does the criterion that the Spearman p be less than 0.05 in the averaged data set. Overall only 6.7% of variables survive the first 6 criteria and 2.5% survive all 7 criteria.

*1.6.3.3* Univariate Results

Formally a correlation is a “bivariate” test but in this context dementia is always one of those variables and so only one variable changes. In order to keep the presentation as clear as possible, the term “univariate” will be used for the correlation testing to embody the idea that only one of the explanatory variable is being tested at a time.

Supplementary spreadsheet 2 shows the results of the univariate analyses for the full data set. Supplementary spreadsheet represents this data organized according to the degree of partial correlation between the dependent and independent variables. A total of 33 variables met the criteria 1-6. Table S7 shows the 19 of the variables with the lowest Spearman p value <0.016 (threshold for FDR=0.05) that were included in the next step of the analysis. Note that the three covariates identified above (age, race and gender) are automatically entered into the multivariate models.

Supplementary spreadsheet 3 contains the same data as in supplementary spreadsheet 2 except that it is sorted according the value the partial correlation Spearman R. Rows are colored from red to green according to this value. Variables with a larger partial correlation Spearman R are associated with higher dementia prevalence and are colored red. Variables with a large negative partial correlation Spearman R are associated with lower dementia prevalence and are colored green. Rows with more intense colors have stronger associations.

It should be noted that the univariate analyses are important in their own right and a variable does not necessarily need to be in a multivariate model in order to be important especially if it is correlated with one the those variables. This will be discussed again in section 1.6.8

*1.6.4 Multivariate Analyses*

One of the critical components to this analysis was using multiple different multivariate analyses to make certain that effects are not dependent on the vagaries of the algorithm and that consistent results are found.

*1.6.4.1* Low Order Exhaustive Regression

The first analysis was an exhaustive evaluation of all regression models (using the independent variables found to be significant in the univariate analysis) with up to 3 of the independent variables (and a constant) and the dependent variable. These variables are ranked by the value of the AIC (Akaike Information Criterion). Since the AIC is related to the relative likelihood of selecting models, only those models that are more than 100 times less likely that the model with the lowest AIC were chosen. These two models are shown in table S8. The strongest effect, as might be expected, is of the fraction of the population aged 65+. This variable is in both of the models and has a remarkably consistent slope of 0.033 (standard deviation=0.006). Table S9 shows each variable that entered into one of the exhaustive regression models.

1.6.4.2 Forward Stepwise Regression

Two forward stepwise models were considered. In the first only the variables identified in the exhaustive regression analysis as shown in table S9. In the second all of the variables identified in the univariate analysis were entered. The forward stepwise multiple regression model was set up with a requirement of p <0.05 to enter or p>0.05 to remove and a variance inflation factor <10 to enter or >10 to remove. If a variable is added but causes a previously added variable to have p>0.05 then that old variable is removed. New variables were entered only if the resulting model resulted in a lower AIC value. Table S10 shows the resulting models. It is important to note that the variables appearing in both models had similar slopes and all had the same sign as in the univariate analysis. This provides some level of confidence that the results are not purely artifacts produced by the uncertainties and singularities present in the covariance matrix. The AIC of both forward models is less than that of the exhaustive regression models and the value of R2 is higher indicating that these models fit the data better. Figure 13(b) is a histogram of the residuals for the larger forward regression model. The variables that appear in one model but not the other are highly correlated as indicated in Table S10.

In order to verify that the models obtained would not have occurred with random data, the forward stepwise regression was carried out after 50 permutations of the components dependent variable (dementia). The forward stepwise analysis was performed with all of the variables identified in the univariate analysis. In these 50 simulations only 10 times did even one variable enter into the final model. While the model referred to above had 8 variables, the average number of variables in the permutation models was 0.2. Thus, finding 8 significant variables would be highly unlikely.

If the selection criteria included the Spearman correlation in the averaged data to be less than 0.05 (criterion #7 above) , there were fewer variables identified as significant in the univariate analysis and slightly different models were created as in Table S11 but the variables in those models that are not in the models of Table S11 show significant correlations with those variables. As expected a number of different multivariable models might create reasonable results when the variables are highly correlated.

Figure S13 (a) and (b) show the residuals for each case as well as a histogram of residual values after the forward stepwise regression derived from all the variables significant in the univariate analysis.

1.6.4.3 Forward Stepwise Regression with noise

In order to see how sensitive the multivariate models are to noise, 20 simulations (each with only 5 not 200 permutations used in the computation of the significance of the univariate models) were performed. To the measured dependent variable, normally distributed noise with a variance that is 1% of the variance in the measured values of the dependent variable is added to the dependent variable. The frequency with which each variable enters the different models is computed and summarized in Table S12. The target variables in this analysis were the variables in the forward stepwise regression analysis beginning with the full set of variables that were found to be significant in the univariate analysis. The table shows the number of simulation in which the given variable appears in the univariate model, the forward stepwise model or one of the exhaustive regression models. The non-target rate is the rate at which non-target variables are found in the specified model divided by the total number of variables.

1.6.4.4 Regularized Regression

As discussed later in this appendix, if it is known that there is uncertainty in the explanatory variables, finding the best fit parameters over all the variations leads to a similar problem as the standard regression with the change that covariance matrix is replaced by the covariance matrix plus a diagonal matrix that has on its diagonal the variance expected for each variable. This is essentially a regularizer that ensures that the regression model has a unique solution since the covariance matrix has non-negative eigenvalues and the diagonal matrix has all positive elements. The price for this is an unknown estimate of the uncertainties in each variable. In order to understand and compare the regression coefficients both the dependent and all independent variables were standardized to mean zero and standard deviation of 1. The regression was then carried out with the noise variance at 0.001, 0.01 and 0.1 of the actual parameter variance and the coefficients found. Figure 13(c) shows the coefficients. The coefficient with the largest value is that for variable 3 which fits with the results of the above analyses. Table S19 shows the averaged ranks of target variables in the regularized regression analysis for simulations with two noise levels. and also shows the true positive and false positive detection rates for those noise levels.

*1.6.5 Correlations*

As noted previously, the independent variables are strongly correlated and so it is important to realize that the variables in the multivariate model may not be the biologically important ones but could be closely correlated with other variables of biologic importance. To this point, in the different multivariate analyses, different variables can enter the regression especially if they are highly correlated. Figure S14 demonstrates the degree of correlation between the variables selected in the univariate analysis is strong and the distribution of those p-values is not uniformly distributed on [0,1] as would be expected if there were no correlations. The probability that the actual distributions are uniform by the Kolmogorov-Smirnov test is <10-12. Supplementary spreadsheet 4 shows the correlations between each of the variables that were significant in the univariate analysis.

*1.6.6 Cluster Analysis*

The process of selecting the optimal explanatory variables from a large list of highly correlated variables is complex and subject to interpretation. The procedure described above involved a large number of steps. First, univariate testing was used to find variables with a high degree of correlation with the dependent variable. This was followed by exhaustive linear regression using variables identified in the univariate model to find those that were commonly entered into the best regression models. Finally, this group of variables was subjected to forward stepwise regression to find the best model. The forward linear stepwise regression was also applied to the full set of variables selected in the univariate analysis.

An alternative approach was to use an algorithm to cluster the independent variables into groups with similar characteristics and then enter the mean value of the variables in each group into a forward stepwise regression analysis that can be used to identify significant clusters. The first step in this process was standardizing the data so that each variable had a mean of zero and a standard deviation of unity. This allows comparisons between and addition of data from variables with different units and ranges. The next task was identifying clusters within the variable list. Two approaches were explored. Both used the Mathematica function ClusteringComponents which allowed the specification of the maximum number of clusters to be found, In the first method, each independent variable was treated separately and the clustering algorithm was applied without any reference to the dependent variable (termed raw clustering). This clustering process was carried out as a function of the maximum number of clusters. The mean value of the variables in each cluster was computed and then entered into a forward stepwise regression analysis with p to enter or remove of 0.05 and VIF to enter or remove of 10. If the addition of a new variable caused the p value for another variable to be greater than 0.05 or VIF>10, that other variable was removed. This procedure was performed with maximum number of clusters varied between 1 and 50. The regression model with the lowest AIC was chosen as the optimal model. The other method for clustering used the list of the Spearman R coefficients between each variable and the outcome variable to cluster the variables (called correlation clustering). Three possible outcomes for significance of a variable were studied: whether the variable was in a cluster that was entered into the forward stepwise regression, whether the variable was in the cluster that entered into the regression with the largest slope or the variable was in a cluster that entered into the regression with the lowest p-value.

In order to understand the performance of this procedure prior to analyzing the actual data of interest, simulated data sets like those mentioned above were chosen. Five significant variables (103, 203, 303, 403, and 505) were chosen each contributing equally to the dependent variable. A low noise level (frax=0.1) was chosen and both clustering methods and all three outcome measures were studied. 20 simulations were carried out in each case.

As can be seen in Tables S13,14,15 there are a number of advantages to using the correlation method to cluster the variables as this leads to a higher true positive rate, a lower false positive rate and a larger number of target variables in the cluster with the smallest p (or largest slope) value in the final regression. It also appears that there is a reasonable ability to detect the target variables with true positive rates between 0.38 and 0.62 depending on the outcome measure which is similar to the previous method. However, the false positive rate is on the order of 0.008-0.03 and is much higher if the outcome measure is that a variable is present in any significant cluster while the true positive rate is not much higher than if a variable was considered significant only if it was in the cluster associated with the smallest p-value. There is also a large difference in the detectability of different variables as well as wide variability from simulation to simulation. Choosing a lower p value threshold or vif threshold for the forward stepwise regression does not make a large difference in the true positive and false negative outcomes. Thus we shall look for potential explanatory variables in the cluster with the lowest p-value using the correlation clustering. The analysis of the actual data will proceed with the p-value to enter or remove at 0.05 and the vif threshold at 10.

With the simulation results, the actual data can be analyzed as documented in tables S14 and S15. It is important to remember that this analysis is applied to standardized data and so the AIC values will not be comparable to those of the other method but the R2 values will be and they are as good as those using the previous method. The one variable clearly identified is the fraction of patients 65+ in age as a risk factor for dementia. Although other variables that were in the forward stepwise regression are present in the model, the simulation results indicated that it would be hard to identify single variables unless they were in the cluster with the smallest p-value.

Although the method does bring together variables with mathematical similarities, it is hard to interpret their meaning unless the clusters themselves have clear biologic identities. This may be a future area of research to create a biologically based clustering method.

*1.6.7 Principle Components Analysis*

Another means of categorizing variables is principle components analysis. The covariance matrix for variables was computed and its eigenvalues and eigenvectors were reviewed. Of note, out of the 519 independent variables, there are only a very few significant eigenvalues. In fact 99% of the power is contained in the space spanned by the first 15 eigenvectors (Figure S15). The variables with the greatest weight in the first eigenvector was “soft drinks, unconcentrated” (variable 285) and in the second eigenvector 190 (Processed fruit and fruit products) and in the third 76 (Meat based ready meals and convenience meat products). Although the principal components analysis provides information on the structure of the independent variables, it provides no information about connections to the outcome variable. Neither does it offer any biological connection between the variables. Thus it is subject to the same limitations as the cluster method.

*1.6.8 Post Analysis Simulations*

Simulations were run after the final models were determined to get better estimates of the overall true and false positive rate of the analyses. In the first, normally distributed random noise was added to the values of the dependent variable predicted from the 8 variables in the forward stepwise model of Table S10. This is done for two noise levels. The noise standard deviation is a fraction (frax) of the variance in the predicted variable. It should be noted that for an R2 at 0.957 as in that model (Table S10), the relative standard deviation of the noise is roughly as in table S10. Thus for “realistic simulations” the relevant value of frax would be 0.2. Simulations were performed with this value of frax as well as frax=0.01 which is a very low noise condition for comparison. 20 simulations were run for the full analysis including the univariate analysis, the exhaustive regression analysis and the forward stepwise analysis. In addition, the results of the regularized regression were interpreted as follows. The coefficients in the regularized regression were rank ordered since the largest coefficients were associated with the largest influence on the dependent variable. Only variance level 0.001is used in computing the regularizer. Since the model to be tested had 8 variables, the regularized regression model was interpreted as detecting that variable if it had one of the 8 largest variables. A variable outside the target variables from the top of the Table S10 was considered a false positive. Table S13 shows that in the low noise condition (frax=0.01) the true positive rate for the univariate model, the forward stepwise model and the regularized regression model is 100%. The true detection rate for the exhaustive regression is 33%. The false positive rate for the univariate analysis only is on the order of 7% and for the forward stepwise analysis 0.003% and 0 for the regularized regression and the exhaustive regression. However, in the more realistic (frax=0.2) noise level, the true positive rates for the univariate analysis was 99%, for the forward stepwise analysis at 90%. The true positive rate for the exhaustive regression was 40% and for the regularized regression at 74%. The false positive rate for the univariate analysis was 5.5% compared with 0.17% for the forward stepwise analysis. The false positive rates for the exhaustive regression was 0% and for the regularized regression at 0.8%. This data is show in table S19.

It should be noted that another important reason for simulations is that the Benjamini-Hochberg method without the Yekutieli correction (not used in these studies) is not accurate in the face of highly correlated data and so the estimates of FDR from that procedure alone may not be accurate.

*1.6.9 Construction of a Single Index From the Food Variables*

Although the above provides some confidence in the results of the forward stepwise analysis, different ways of framing the model and still lead to different results. Most of the methods explored involved the selection of specific variables either through univariate testing, or multiple regression analysis or clustering. Another approach that is blind to the selection process is to create a single variable from all of the food elements weighting them on the basis of the univariate analyses. To see how well this works, each food variable was standardized and a new variable was created by multiplying each standardized variable by the value of R from the partial correlation Spearman rank correlation (Residuals after removal of age, race and gender from bot the independent and dependent variables were computed and then the Spearman rank correlation test was applied to the residuals). Section 3.5.1 indicates that this would be the exact solution in the absence of correlation and a complete set of explanatory variables. Thus, variables that had a strong positive correlation with the dependent variable were weighted by a factor close to one. Variables that had little correlation with the dependent variable had weights near zero and variables with a strong negative correlation had a weight near negative 1. This global food index variable along with age race and gender were entered into a forward stepwise regression analysis with the results shown in table S20. There is a very strong correlation between the dietary measure (Figure S16) although race did not enter the final model. Over 200000 simulations found that using permutations of the weights discussed above or randomly assigned weights between -1 and 1 never produced and R2 as high as that obtained in the specified model, confirming that this is not a chance association. An additional set of 50000 simulations were performed starting from the R weightings adding a uniformly distributed random variable ranging between -0.5 and 0.5 to each weight, forcing all weights to have the same sign and be limited to [-1,1] (if the proposed weight was less than -1 it was set to -1. If the proposed weight was greater than 1 it was set to 1.). 24% of these simulated weights gave better regression R2 values than the partial correlation weights. The AIC drops only from -913 to -924. Figures S16 (a) and (b) shows the relationship between the food variable produced with partial correlation weightings (a) and the random weightings (b) associated with the lowest AIC in the multiple regression analysis. The graphs are similar and S16(c) show the weights for each simulation where the top trace represents that with the lowest AIC. There is no consistent pattern of change in the as a function of AIC. In order to do a broader search for better weights, in the next set of 50000 simulations random noise that was uniformly distributed on [-0.5,0.5] was added and the best fit model computed. However, this set of simulations was iterative and the noise was added to the set of weights with the lowest AIC to that point not the R weighted weights although that was chosen as the starting point. Figure S16(d) shows that there are substantial changes in the weights for the first few thousand iterations but then the weights are stable and the procedure converges. Figure S16(b) shows the relationship between the food variable created with the optimal weightings and Dementia QOC. The graph is very similar to that obtained using the R weighted coefficients but does show a more linear relationship. The AIC in this best model drops to -936 and the value of R2 for the multivariable regression increases from 0.918 with the R weighted coefficients to 0.941 with the optimal weights. In order to see how dependent the final result was on the exact details of the iteration another set of 50000 simulations was carried out this time not changing a weight if a proposed value was >1 or <-1. The results are shown in Figure S16 (e) and (f). The AIC for this model drops to -942 and is associated with R2 for the final regression at 0.946. The food variable has a stronger linear relationship to dementia. The problem is that the final solution is very much dependent on the details of the iterative procedure and as show in in S16(g) gives very different weightings with only moderate improvements in the overall degree of fit.

Although the idea of a single index was inspired by the need not to select variables, for the sake of comparison another set of weights was determined as above except that all weights associated with variables with a partial correlation less that a certain magnitude were set to and remained at zero during the iterative process. In order to see the implications of this procedure, the above analysis was repeated with the threshold of 0.6 so that all variables with a partial correlation between -0.6 and 0.6 were excluded. In this model there were only 13 variables with non-zero weights. Table S20 shows that before the iterative process the multiple regression model with this food variable had a lower R2 and larger AIC than the other models but after the iterative process was associated with a better fit than the partial correlation weights applied to all of the variables. Figure S16 (h) and (i) show the relationship between these food variables with dementia before and after the iterative process. The linear correlation between both of these food variables with dementia prevalence is good. This is especially true with the that with the iteratively chosen weights. Figure S16(j) and (k) show the chosen weights before and after the iterative process. Table S20(b) shows the weights for each of the variables in this particular computation of food index. The fact that there is good predictability of dementia based on only 8 variables is encouraging for future studies but only additional studies could verify the predictions and the variable selection. It is encouraging that some of the most highly weighted variables did appear such as fruit intake and cooked rice did appear in some of the multivariable models. However in no case did the weighted food variable have a better correlation with dementia than some of the individual variables. Again, only additional data can help sort this out.

*1.6.10-Connections between the first and second studies*

In the first study, dried rice consumption, total rice consumption were the two variables singled out as having the most significant relationship to dementia. Mineral water, salmon and bacon consumption were also in the set of variables with the highest correlation. These variables were not considered significant in the second study. The purpose of this section is to determine why. Table S24 shows the p-values associated with each Spearman rank correlation between the variables in the two studies. This shows a strong relationship between the rice consumption and age 65+. Figure S22 (a) shows the Spearman correlations as well as the p-values (b) with the variables in the second study on the y axis and the variables in the first study on the x-axis. Figure S22(c) shows the distribution of p values and their CDF (d) which is clearly different from the linear function expected if there were no correlations. The Kolmogorov-Smirnov test shows that the probability that the distribution of p-values is uniform on [0,1] is 6x10-11. Figure S23 graphs the relationships between these variables. This shows clearly that rice consumption decreases significantly with age. Table S24 also shows the value of R2 from a multiple regression analysis in which each variable in the first study is the dependent variable and the independent variables are those in the second study. These values are large and suggest that the first study variables are a strong function of the second study variables. In particular, it is important to look at the graph of each first study variable against the fraction of the population aged 65+. Most of the slope relates to the fact that there are two clusters of points at lower and higher ages rather than a continuous distribution. Slight changes could affect the slope significantly and would be more than enough in a study like this to account for any differences especially since the fraction of the population aged 65+ is the strongest predictor variable.

*1.6.11—Connections to other studies*

There are a number of other studies of diet and dementia. It is common for studies to look at the DASH diet {{2305 Blumenthal,J.A. 2019;}}, the Mediterranean diet{{2442 Hu 2020;}} and the MIND diet{{2440 Morris 2015;}} {{2435 de Crom,T.O.E. 2022;}} {{2423 Munoz-Garcia,M.I. 2020;}} {{2296 van den Brink,A.C. 2019;}}. The association between an inflammatory diet and cognitive function has also been explored {{2445 Hayden,K.M. 2017;}} {{2436 Charisis,S. 2021;}}. In general some studies show strong effects and others show no or weak effects of these diets on cognition. This is consistent with the findings of this study in Tables S25 and S26 showing significant differences between the predicted effects of certain food consumptions from those large diets and this particular study. The problem with the large dietary studies is that the consumption of a different food types and subtypes may be correlated so that only a group effect is significant. This is illustrated in the current study where the strongest point in Table S25 is that within each larger food categories there are dietary elements that have widely varying associations with dementia prevalence. This leads to conflicting results where the current study suggests a higher risk of dementia with poultry and fish on average but the large diets suggest the opposite effect.

Further illustrating the complexity of the issues surrounding dietary effects on dementia is a study by Takeuchi {{2434 Takeuchi 2021;}} that showed non-linear effects of certain dietary intakes on dementia risk. For example, moderate consumptions of meat were associated with lower risks of dementia while the highest intakes of cheese and bread were associated with the lowest risks of dementia. These findings are consistent with the findings in this study but the effects of meat and cheese are different than what would be predicted from the effect of the DASH, Mediterranean or MIND diets. Takeuchi {{2434 Takeuchi 2021;}} also found a linear increase in risk for dementia with increased vegetable and fruit intake which differs other studies noted. However, the current study sheds some light on why this may be true as it demonstrated that certain fruit and vegetable products were associated with a higher risk of dementia and others with a lower risk. It may be that there are differences in the type of vegetables consumed in the different populations.

**1.7-Summary/The Future**

It is clear that there are significant effects of diet on dementia prevalence even after correcting for age, race and gender. This is best shown in the analysis of the R2 variable for the Spearman R values after the effects of all three covariates have been removed from the dependent and independent variables. These values are much larger for the actual data than for any of the permutations of data. This test only demonstrates that there is an effect and does not specify any details. The univariate and multivariate models reveal strong and consistent relationships between age and dementia as expected. They also reveal statistically significant effects of the food variables.

Consistently in the univariate models increased consumption of: cooked rice, root vegetables, convenience meat products, alcohol intake and many others are associated with a higher prevalence of dementia while sugar and pure fruit juices are associated with a lower prevalence. However, the list in supplementary spreadsheet2 is reviewed there appear to be some inconsistencies. For example, beef veal and pork sausage consumption seems to be associated with a lower prevalence of dementia but the consumption of hot dogs and convenience meat products is associated with a higher prevalence. Could this be the identification of false positives or could this effect be explained by preservatives in the convenience meat products. Only additional studies will answer this.

The various multivariate models reveal a few important issues. First, linear models that predict the prevalence of dementia include food items in a very significant way. Second, the different methods identify different variables of greatest importance in predicting the prevalence of dementia. This is likely due to the presence of strong correlations between the variables. Additional data over time may provide further insights into the results. In addition better clustering algorithms and more comprehensive multivariate analyses will be helpful.

Perhaps the best model that can be extracted from this data is the simplest that does not require any variable identification with its intrinsic problems. This is choosing a simple weighted measure of the dietary elements. This seems to perform well, is simple and intuitive and has none of the problems associated with the hit or miss issue of variable selection. The goal of this pilot study was to generate hypotheses. The simplest hypothesis is contained in supplementary spreadsheet3 which contains a rank ordered list of foods that may contribute to dementia. A prospective study using this as a basis could provide powerful and important information and with more information maybe extract food elements with the biggest contribution to the prevalence of dementia. However, different methods of generating the weights yield very different values with similar predictive values. Thus, only additional large scale studies with more cases, large detail on dietary intake would be needed to be certain of trends and effects.

**2.0-Original Methodology/Results**

**2.1 Data Sources**

The data for this study was taken from various publicly accessible sites from the UK that are detailed in Table S21. All of the links shown were active at the time of the original submission. The information on food purchases and nutrients came from the UK Family Food Survey. The methodology of this study is detailed at https://assets.publishing.service.gov.uk/government/uploads/system/uploads/attachment_data/file/384770/familyfood-method-about-11dec14.pdf. In brief, the surveys are given to all members of selected households and represent food purchased confirmed by purchase receipts not food consumed over a two week period. The purchased food is divided into food that enters the household (“Household Purchases”) and food that is purchased outside the home (“Eating Out”) and then is reported in amounts per person per week. In 2014 the survey collected food information on 121,250 people in 5,144 households in the UK. The intake of various nutrients both as absolute values and as a percentage of weighted reference intakes was then computed based on the Composition of Foods Integrated Dataset https://www.gov.uk/government/publications/composition-of-foods-integrated-dataset-cofid. Data was available for each of the 9 Regions of England (North East, North West, Yorkshire and the Humber, East Midlands, West Midlands, East of England, South East, London, South West) as well as the UK countries: Wales, Scotland and Northern Ireland. Data used in this study came from the year 2016/2017.

All data was publicly available but not from a single database that encompassed all geographic areas under study. It was common that one database had information about the regions of England, while another had information at an aggregate level for all countries making up the United Kingdom. Given this, the primary analysis concentrated on the variations from region to region within England for which all data can be taken from a single database. However, a secondary analysis was performed using data normalized and merged from different databases. In this analysis, the information about a variable that came from a country level database; was multiplied by the ratio of the variable average from the regional data divided by the variable value for England in the country data before entry into the analysis.

Estimates of the prevalence of dementia for the regions of England and the other UK countries, (Wales, Scotland and Northern Ireland) came from different sources (Table 1). The primary data elements were the total number of patients aged 65+ with a diagnosis of dementia in a registered medical practice in the given region divided by the total number of patients with ages 65+ registered with medical practices in the region. A similar number defined the prevalence of dementia in the group aged less than 65. This database also contains an estimated dementia diagnosis rate which is the fraction of expected dementia diagnoses that were actually recorded. The percentage recorded for England was 68.7%. This data was from 2019 and only the ratio of the total number of patients with dementia to the total population in the region was entered in the analysis. For Wales, Scotland and Northern Ireland, only an estimate of the total number of patients with dementia was available from 2017 to 2018 (Table S21).

For each region/country, the total population, population density, median age, birth rate, death rate and % of population aged 65+ for 2018, were taken from the Office of National Statistics (ONS) (Table S21). Ethnicity data and information on the gross disposable household income (GDHI) index for 2017 was taken from the sources in Table1. The data from the regions of England for hypertension, diabetes, coronary heart disease, stroke, obesity, depression, smoking and physical inactivity were taken from the sources shown in Table S21.

Epilepsy is another neurologic disorder with a very different pathophysiologic basis, and it was included as a control because it would not be expected to be related to diet in the same way as dementia. Estimates of the prevalence of epilepsy were not available by region in England, but were estimated from prevalence estimates in smaller regions (CCG-Clinical Commissioning Group) from the 2010 and 2015 data shown in Table S21.

**2.2 Statistical Analysis**

Once all data was collected in a single spreadsheet, variables from the UK family food with missing or incomplete data, were eliminated before analysis. The data was grouped into three types of variables. The first was a dependent variable (dementia or epilepsy prevalence). The second was a set of covariates including all demographic and risk factor data, and the third were the target variables providing information on the food and nutrient consumption. There were 22 covariates and 657 diet/nutrition related target variables analyzed.

*2.2.1 Correlation (Univariate) Analysis*

Because of the large number of variables to be tested, careful attention to the effects of multiple testing needed to be taken into account. The first step in this process was determining the degree of correlation between each variable and the dependent (dementia or epilepsy) variable using the Pearson correlation test and recording the resultant correlation coefficients and p-values. The parametric Pearson correlation was used in preference to the non-parametric Spearman correlation in this initial search for significant associations because of the limited number of possible ranks in the data. However, parametric correlation tests are susceptible to outliers and so no correlation was considered significant unless both the Spearman rank correlation and the Pearson correlation tests were associated with a p-value<0.05. A p-value resulting from a Pearson correlation will be identified as pP and a p-values resulting from a Spearman rank correlation will be identified as pS.

Before additional analysis was performed, a simple global test for the presence of any effects was performed. A bootstrap distribution was created by repeating the above calculation with random permutations of the dementia prevalence across the different geographic areas. The Kolmogorov-Smirnov test was applied to determine whether the distribution of p-values produced in the above analysis was uniform or equal to the bootstrap distribution. If the probability of the null hypothesis that these distributions were the same was less than 0.1 then the analysis proceeded. If not, the data would have been reviewed to make sure that many variables without biologic significance were not included. This step is done prior to identifying variables that might be linked to dementia.

In the next phase of the analysis, factors with significant correlation were identified using the Benjamini-Hochberg method {{1976 benjamini 1995;}} applied to the pp values with a false discovery rate (FDR) set at 0.05. As above, no factor was considered to have a significant relationship with the dementia prevalence unless both pP and pS were less than 0.05.

*2.2.2 Multiple Linear Regression*

The second analysis involved taking the variables in the univariate analysis that were correlated with the dependent variable with a p<0.01 up to a maximum of 25 and entering them into a multiple linear regression analysis. Because of the limited number of cases and the possibility of significant correlations between variables, only those regression models involving 0,1 or 2 covariates and 1 target dietary variable, were considered. The “best” 4 models were determined as those with the lowest Akaike information criterion (AIC). Only the top 4 models were studied because of the limited power of multiple regression. The p-value for significance in the regression analyses was taken as 0.05/number of tests performed in those 4 models. The slope and standard deviation of the slope for each variable in the model was also determined. In addition, because of the possibility that strong relationships between the explanatory variables, the variance inflation factors (vif) for each variable were computed as well. It was taken as likely that there was significant collinearity if the variance inflation factor was greater than 10. In this case, the given regression variable was not counted as having a significant relationship to the prevalence of dementia. These analyses were performed primarily on the data from the different regions of England and secondarily on all UK areas.

*2.2.3 Principal Components Analysis*

Because of the large number of variables and the small number of measurements, dimensional reduction techniques could be helpful in understanding the implications of the data. Two general categories were considered, those based on finding numerical patterns in the data and those based on *a priori* characteristics of the data. The first dimensional reduction technique was based on a principal components approach. Before computing the covariance matrix, each variable was standardized by subtracting its mean and dividing by its standard deviation. Scree plots and plots of the factors in each eigenvector are created.

In order to see how much of the information about the dementia prevalence is contained in the specific patterns specified by one of the eigenvectors, the variable data from each geographic area was projected the eigenvectors. The degree of correlation between the result and the dementia prevalence was measure of how much each pattern related to the dementia prevalence.

*2.2.4 Variable Reduction Procedures*

When dealing with a large number of variables and performing multiple testing, it is possible that the initial choice of variables plays a significant role in the results. In order to control for this, the family food data contains two *a priori* methods for reducing the number of variables. The first of these is based on a reliability estimate made for each variable. A marker (termed a tick) is placed on each variable of in the family food database to indicating the Relative Standard Error (RSE) for each variable. At least one tick was placed if the RSE was less than 10%. If the RSE for a given variable was greater than this or not computed either a blank or an x was placed in each row. In order to see the effect of the variables with larger RSE a secondary analysis was performed including only the reduced number of variables with RSE<10%.

Another natural approach to data reduction involves noting that the family food data does include major categories, and then sub-categories, and not just individual variables. One natural choice is to analyze only the demographic/risk factor data plus the 22 headings in the purchased food database.

**2.3 Availability of Data and Materials**

As detailed above all of the source data was publicly available at the listed websites and governed by the Crown copyright which allowed free-reuse of the data on these websites as long as the following citation is present (Public Health England. Public Health Profiles. 12/05/2021 https://fingertips.phe.org.uk © Crown copyright 2021). In addition is recommended that the following statement be included as well: the data is public sector data licensed under the Open Government License v3.0 (https://www.nationalarchives.gov.uk/doc/open-government-licence/version/3/). The datasets used and/or analyzed during the current study are available from the corresponding author on reasonable request. All data analysis was carried out using programs written in Mathematica (Wolfram Research Inc Champaign, IL). Programs are submitted with this manuscript.

# Results

Figure S17 (a) and (b) show the distribution of pP-values describing the correlation between the variables in the study and the prevalence of dementia. The Kolmogorov-Smirnov test indicate that the probability that this distribution is uniform is on the order of 1.4x10-23. Figure S17 (c) shows the observed cumulative density function is significantly the uniform distribution or the bootstrap distribution with more small pP-values observed than expected in the absence of a relationship between dementia prevalence and any of the factors. Figures S17 (d) and (e) show that difference between the observed distribution of p values and the uniform/bootstrap distributions is greater for the demographic/risk factor covariates than for the target dietary variables although a large and statistically significant difference is still seen.

In the univariate analysis (Table S22), there were 20 variables with FDR<.05 and a Spearman rank correlation <.05. These included 10 demographic risk factors mainly related to the racial composition of each region, population and birth and death rates. Overall, increasing numbers of ethnically white British in the population was associated with increased dementia prevalence. Increasing numbers of other races was associated with a decreased dementia prevalence. Increasing median age and fraction of the population 65+ were also associated with an increased prevalence of dementia. Increasing death rate and decreasing birth rate were associated with an increase in the prevalence of dementia. Three medical risk factors were in the list of significant variables. Increasing prevalence of coronary heart disease, hypertension and stroke were associated with an increasing prevalence of dementia. Seven dietary factors are significant in the univariate analyses: Increasing consumption of rice, salmon and water are associated with a lower prevalence of dementia while increased consumption of bacon and ham was associated with an increased prevalence. Figure S18 is a graphical representation of the relationship between dementia prevalence and a number of the above factors. None of the global nutrient variables had a significant relationship with the dementia prevalence and all of the most significant dietary factors were related to household purchases and not food eaten out.

Figure S19 is a heat map further clarifying the relationship between the demographics and risk factors, dementia and the target food variables. The first row of this map shows the Pearson correlation coefficients for the dietary factors with all of the dietary factors re-organized so that the first has the highest correlation with dementia and the last the lowest. The rest of the rows represent the correlation coefficients describing the relationship between the dietary factors and the covariates. It is clear that on an overall scale many of the covariates relate to diet in a way that is either similar to that of dementia or opposite. However on a finer grain of individual variables, there are differences. This underscores the importance of a detailed analysis of many dietary factors. A supplementary spreadsheet is available that highlights every element in the UK family food database according to the Pearson R value showing the relationship with dementia prevalence. It is clear that there are clusters of similar effects but even within the same food category there are large differences in the relationship with dementia. Figure S19 shows the degree of correlation between all of the factors considered significant with FDR<.05.

Although the multiple regression analysis was limited by the small number of cases in the data, Table S23 shows that the best linear models show significant and independent effects of both Asian ethnicity and rice consumption in predicting dementia prevalence. However, there is significant correlation between rice consumption and Asian race (RP=0.87), hypertension (RP=0.94), and coronary heart disease (RP=0.91). When hypertension and Asian race are included in the model the variance inflation factor for rice consumption suggests that there is collinearity. However, the slope of the relationship between rice consumption and dementia prevalence is similar in all the models and so this relationship is likely real and independent of race. The other multiple regression models did not show a significant improvement of fit when dietary factors other than rice consumption were added.

Only a small number of the factors that had significant relationship to dementia prevalence (Table S22) were related to epilepsy prevalence (Table S27).

The principal components analysis showed that >99% of the variance was accounted for by the first 9 eigenvalues (Figure S20(a)). Projections of the (covariates plus dietary) data onto the eigenvectors revealed only a single eigenvector, the first one, for which the there was a strong correlation with the dementia prevalence (Spearman RS=-0.96, pS=5.8x10-5). The first eigenvector shows the expected heavy weighting (Figure S20(b) toward the demographics risk variables, but many of the dietary variables have significant weight. Table S28 shows the 20 variables with the largest factor weights in the first eigenvector. These include ethnicity and population density. The factors also include rice and fish intake, which was significant in the univariate FDR analysis but included many other factors such as the intake of cereals, meat and fish soups, other fresh vegetables, offal and dried seeds. The variance of the residuals drops significantly when more than the 125 variables with the largest weights are included in the classification vector suggesting that these are the most significant.

Regarding the effect of the two *a priori* data reduction techniques, a univariate analysis of only the food variables marked with low RSE (putatively more reliable) with appropriate reductions in the number of tests continued to show significant effects of the demographic/risk factor variables but none of the food variables were significant (FDR<.05, Spearman R<.05). Analysis of only the demographic/risk factor variables and the 22 top level food groups from the household purchases did not reveal any dietary variable that met criteria for significance.

**2.4.1 UK Data**

For the reasons described above, the data from the entire UK was felt to be less reliable than the data from only the regions of England. Although the global test indicated that the probability that the distribution of p values was uniform or the same as the bootstrap distribution was low, no data point was significant with FDR<.05. This indicated that there were significant effects but does not specify what they were.

**3.0-Statistical Analyses**

**3.1-Introduction**

The purpose of this part of the appendix is to lay out some general information about how the analyses used in this paper especially those related to variable selection. Secondarily, results of simulations are shown that clarify the statistical problems posed in more detail.

**3.2-Effects on Outcome and Predictive Modelling**

Finding a relationship between a few outcome variables and many possible explanatory variables is extremely complex and can result in false negative and as well as a large number of false positive results. In all cases the first step is to choose the set of explanatory variables. Some studies approach this problem by a priori choosing a very small list of explanatory variables. Studies of this type ask for example whether adhering to the MIND diet could change the probability that a person would develop dementia. This makes it possible to test simple hypotheses using standard statistical techniques. It also has the advantage of easily lending itself to controlled and randomized trials. Another type of study, such as that pursued in this paper, begins with a large number of explanatory variables. The major problem with studies like this is that when there are correlations between the explanatory variables (which must always exist if the number of variables is greater than the number of cases) it is impossible to find a unique relationship between the variables and the outcome. However, the advantage is that it lessens the problems introduced by a potentially biased a priori variable selection.

**3.2.1 A Simple Model**

It is useful to consider a simple model to help understand the effects of variable selection. Suppose that there is a single outcome variable measured in N different populations and that there is a universe of M possible explanatory variables. Consider the case in which only one of the explanatory variables (a) actually has a causal relationship with the outcome variable although we don’t know which one. Take p0 as the probability that a univariate test for association between this variable and the outcome variable would occur under the null hypothesis. The other variables take on random values. Two types of statistical experiment will be considered to find this variable: a selective and an inclusive experiment. In the selective experiment, the investigator chooses only one of the M variables for analysis based on some degree of a priori knowledge. This is similar to asking the question as to whether a specific diet has an effect on dementia. In the inclusive experiment, the investigator analyzes each variable, and chooses only the single variable with the lowest probability that its relationship with the outcome variable would occur by chance. In other words the variable with the lowest p-value in the univariate association test is chosen. This is analogous to the current study where many food variables are studied.

In the selective experiment, it is important to quantify the investigator’s a priori probability of choosing the correct variable from the M possible variables. One way to do this is to assign an intrinsic weight to each wi to each variable which is larger in variables that a priori are thought to be more likely related to the outcome variable. For example, w1 is 10 times w2 then the investigator had felt that variable 1 was 10 times more likely to be in the model than variable 2. It is important to realize that these are just guesses and so it is unlikely that an honest investigator could be able to assign weights that were more than 10 or 100 times greater for one variable than another without having done prior analysis or calculations. The probability that the investigator will select the correct variable is:

For the sake of simplicity we take the simple situation in which the weights for each variable except a is 1 and the weight for variable a is wa.

A univariate statistical test is applied to the selected variable and a threshold t [0,1] is chosen for significance. Thus, in this case the probability of a true positive selection is:

The probability of selecting another variable is:

Since the p-values for the random variables are uniformly distributed .

In the inclusive experiment, a true positive is found only if the threshold for significance is greater than p0 and there are no smaller p-values among the other variables. Thus:

The false positive rate if the threshold is below p0 is the probability that one of the random variables will have a smaller value. When the threshold is greater than p0, probability of choosing the wrong variable is just the chance that another variable has a p-value less than p0:

Note that if p0<<1 then:

If the value of the threshold is selected so that the FDR (false discovery rate) is less than pfdr then in the selective experiment t much be chosen t<tfdr:

There will be no positive detections until:

And the true positive rate will be:

For the inclusive model the lowest value of the FDR which gives true positives is:

Which is associated with a true positive rate of:

So, the selective model can be associated with detections at a slightly lower FDR than the inclusive model although this is not a large effect but the true positive rate is very different. If w exceeds wT then the selective model has the higher true positive rate where:

Table S29 shows some values of wT for different values of p0 and M. However, in the limit :

So, when (M-1)p0 is small very large values of wT would be needed for the selective model to attain the same true positive rate. However for large values of p0:

and so for vary large values of M, the true positive rate is better in the selective model even with marginally better weights for the correct variable. Table S29 illustrates the weight required for the two methods to achieve the same true positive rate while maintaining .

This demonstrates that there are situations in which the inclusive model can be better than the selective model. In the statistical experiment reported in this paper M is less than 1000 and p is less than 0.001 so that the inclusive model would be better if a single variable were being selected.

Note that the above analysis referred only to the situation were was only 1 significant relationship and only univariate testing was performed. With the inclusive model, the false positive rate can be lowered significantly using multivariate methods.

**3.2.2 A More Complex Situation with Simulation**

It is helpful to look numerically at the more complex situation in which there are more than 1 variable that contribute to the outcome. In the simulations that follow there are 100 variables each of which have 10 cases. Each of the variables was taken as a normally distributed random variable with a mean of zero and standard deviation of 1. The outcome variable is taken as the sum of Nin variables that were chosen as significant plus normally distributed random noise with a mean of zero and standard deviation 1 multiplied by a factor, f. When f is small the noise is small and the correlation between the outcome variable and the explanatory variables in the model is high. When f is large the correlation between the outcome variable and the explanatory variable is low. The variables for inclusion in each simulation were chosen according to weights applied to each variable. For the inclusive model the weight associated with each variable is 1. For the selective model the weight associated with the variables in the model is w it is in the group of variables a priori known to be in the model and 1 otherwise. Only univariate testing was used to find relationships in the inclusive model.

Figure S24 shows the relationship between the true positives and false positives identified in the model as the p-value threshold for significance (t in the above example) varies from 0 to 1. This is the receiver operating characteristic curve (ROC). The outcome variable is a priori chosen as the sum of two variables. In the inclusive model, where all variables are tested individually against the outcome (S24.a and S24.g), the expected result is found. For low noise levels, the number of true positives for a given false positive level is greater than expected by chance which in the ROC curve the straight line connecting (0,0) and (1,1). With high noise levels, the number of true positives is equal to the number of false positives as would be expected in the absence of a relationship between the outcome and the explanatory variables. It should be noted that even in the presence of very little noise the ROC (receiver operating characteristic) curve does not have an area under the curve (AUC) approaching 1 as might be expected since the outcome variable is related to two variables and the inclusive model uses only univariate tests.

The outcome of the selective model simulations is more complex. The first observation is that the percentage of true positive results is very low whenever the weighting factor is less than 100 or so. This is exactly what would be expected based on the following argument. The maximum number of true positives is primarily determined by the probability that the variables selected are the correct ones. In general the probability that all of the Nin variables are selected is (if Nin<<M):

This means that the a priori weight assigned to the actual variables in the model must be on the order of a few times number of variables in order to have a reasonable chance of choosing all of the correct variables. Although, under exceptional circumstances an investigator might be able to correctly say that one variable is 10 times more likely than another to be in the model on the basis of intuition, it is hard to believe that a priori weights on the order of or greater than 100 can be assigned by that means. Thus, the selective model fails dramatically when there are a large number of variables. In a problem such as that addressed in this paper with 600 explanatory variables and say 4 variables in the model (age, race, and two dietary factors), a weight of 100 gives only 2% chance of an accurate selection while a weight of 1000 gives a 57% probability of selecting the correct variables and a weight of 10000 gives a 94% probability of selecting the correct variables. The selective model generates a very low true positive rate when the a priori weights are not very large.

As the true positive rate in the selective model increases with w, the maximum false positive rate decreases so that the dependence of the number of true positives on the number of false positives becomes more acute as w increases. Overall, the effect of the noise level on the ROC curve is less than that of the a priori weights in the selective model while the noise level has a very significant effect in the inclusive model. The ROC curve for the selective model begins to approach that in the inclusive model when w is on the order of magnitude of the number of explanatory variables and is associated with a smaller AUC value (and hence worse) if w is smaller. Thus for a large number of potential explanatory variables in the face of w<M, the inclusive model provides the optimal ROC curve. This is a rough criterion for the selective model to be chosen over the inclusive model when looking for significant effects.

Figure S17 and figure S18 show the ROC curves for high and low noise conditions and different weights when Nin=3 and 8 respectively. As expected the ROC curve in the inclusive model drops toward that which would be seen in the absence of a relationship (true positive rate=false positive rate) as the number of variables in the model grows. The ROC curve for the selective model shows minor changes with the major effect being the value of the a priori weights.

In the end, both the selective models and the inclusive models have severe problems as the number of variables increases. The inclusive model has the advantage that it is easy to understand its statistical limitations while the selective model relies on an a priori selection weight for the explanatory variables that may be difficult or impossible to estimate. In fact, the above simulations demonstrate that there must be an extremely high degree of certainty in the a priori selection process or else the statistical properties of the model are poor. In addition the ROC curve for the selective model is most strongly determined by the a prior weights which makes it strongly susceptible to bias.

This brings up the question as to whether a hybrid approach can be helpful. This will be addressed in the next sections through the viewpoint of linear equation solution.

**3.3-General Model Formulation**

The attempt to find a relationship between a few outcomes and many explanatory variables is an important problem that is classically ill-posed {{2280 Tikhonov 1987;}}because there is no unique solution, especially when there are more explanatory variables than outcome variables. This does not mean that there are not reasonable solutions just that additional information must be supplied about the relative value of different solutions. Problems of this sort arise in solving the electromagnetic inverse problem {{2431 Grech,R. 2008;}} and in imaging applications. The purpose of this appendix is to place the problem of finding dietary influences on dementia in context with these more general approaches and then to outline the specific approaches taken in this paper.

Many medical studies attempt to find a relationship between an outcome variable and a set of possible explanatory factors specified for each case. If the number of cases (in this particular example: regions of England) is nc, then the outcome variable can be written as . If the number of factors (diet, demographics, etc.) studied is nF, the explanatory data is contained in the array . Finding the relationship between the explanatory variables and the outcome variables involves solving an equation of the form:

where the elements of X specify the connections between each data variable and the outcome. In fact, the most general problem of this type can be cast in the above LINEAR form. For example, no constant term is explicit in (1.17) but adding another explanatory factor containing nc 1’s and increasing nF by 1 will incorporate this. Also, if it is believed that Y may depend on a non-linear function of the explanatory variables, that may be added as another factor. More generally, the most general relationship between the outcome and the explanatory variables is:

where the explanatory values and the outcome variables are known and it is desired to find the which specify the relationships between the outcome and explanatory factors. It is important to recognize that this is a purely linear relationship between the explanatory variables and the outcome variables. If only first order effects are considered, this simplifies to:

When up to second order effects are considered, it is possible to define:

So that:

The same logic can be extended to the higher order terms. Thus, the general relationship between the outcome variables and the explanatory variables is linear as long as it is remembered that the group of explanatory variables in the analysis can be chosen to include any function of the measured explanatory variables as well as the raw variables. For the sake of simplicity the relationship between explanatory and outcome variables can be written as:

It should be noted that the use of equality in (1.22) is not the only way to choose the values of X. Other choices could include:

1. choose X to minimize the distance between Y and AX
2. choose X to maximize the correlation between Y and AX
3. choose X to optimize the mutual entropy between Y and AX

At a deeper level, the above is a search for the conditional probability of various outcome values given explanatory variables:

Where is the probability distribution for the outcome variable, is the probability distribution of the explanatory variables, and is the conditional probability of an outcome value given the values of the explanatory variables. In short-hand form it would be possible to write:

where it is understood that is an integral over all possible explanatory data matrices each weighted by an a priori probability . In this context, (1.22) results if the conditional probability functions are restricted to functions of the form:

for some choice of the unknowns xi that produce the best fit answer to (1.22) .

In the next section we will discuss the linear solution model as in (1.22) and its role in isolating dependencies of outcomes on explanatory variables.

**3.4-Reformulations of the General Model**

3.4.1 Introduction

There are two problems with the general linear model (1.22). One is that for certain values of A and Y, there may be no solution. This problem is traditionally overcome by using the least squares (or another method) to determine the value of X (termed XLS) that minimizes the difference between and . Although the least squares problem always has a solution, there may be many solutions and the solutions may be highly influenced by small changes in the value of . This is especially problematic when the number of potential explanatory variables nF is larger than the number of cases nc where the number of solutions to (1.22) can be infinite. In this case, finding the variables X that predict the outcome Y is critically dependent on finding either the optimal set of variables to be included in the matrix A (at most nc) or finding ancillary criteria to restrict the solution space. This can be especially difficult since the discussions in section 1 demonstrate that any sort of a priori selection process leads to many problems.

In this section, the advantages of different least squares based reformulations of (1.22) will be discussed.

*3.4.2—*The Least Squares Formulation

The simplest reformulation to solvinginvolves finding XLS that minimizes

The value of X that minimizes I in (1.26) is determined by:

Where is the set of regression parameters which minimizes the least squares distance I. One difference between the equation and is that, although Y has dimension of nC has the dimension of nF. Another difference is that, unlike A, C is a real symmetric, square matrix of dimensions nFxnF. The values of and have clear interpretations based on the data. If (the data in the j’th column of A) then:

Thus, C is the covariance between the different explanatory variables and is the covariance between the outcome variable and the explanatory variables. These physical interpretations will become critical to finding optimal solutions in the following sections.

3.5-Residuals and Measures of Influence

The formal solution to (1.27) is:

However, the inverse of C does not always exist and so the above solution is applicable only when it does. However, the Moore-Penrose or pseudoinverse always does and is unique. In terms of , the general solution for is:

where is any vector. This is true because as part of the definition of the Moore-Penrose inverse. The predicted values of Y, predicted from the least squares solution are then given by:

Since and so the residuals are given by:

More intuition into the meaning of (1.31) and (1.32) can be found by using the singular value decomposition of the matrix A (which always exists):

where U is a unitary matrix of dimension ncxnc and V is a unitary matrix of nfxnf dimensions. D is a rectangular matrix of dimensions ncxnF that has non-negative values on the main diagonal. The element can be chosen so that the they are in decreasing order. The least squares solution for the regression coefficients and residuals is:

where E+ is a square matrix of dimensions nFxnF whose elements are the reciprocal of all elements in E that are non-zero and zero for all elements of E that were zero. is a square matrix of dimension ncxnc zero everywhere but on the main diagonal where elements can be either 1 or zero. If nC>nF some of the elements in F are zero and the residual cannot be zero independent of Y. If nC<=nF then it is possible to for there to be zero residuals for any value of Y if the number of non-zero elements in D is greater than nc. H is the idempotent ncxnc “hat matrix” that projects Y into the subspace with non-zero singular values. This provides significant insight {{2433 Hoaglin 1978;}} into the residuals associated with the least squares solution to the problem (1.22) in particular into which cases contribute most to the magnitude of the residuals (when such exists).

The average of the diagonal elements of H . If any hat diagonal element is greater than twice this, that case is generally considered to be influential since mean square magnitude of change in the residuals by deleting case k is HkkYk2. Note that since D is not generally square the number of entries on the main diagonal of E that are non-zero cannot exceed the minimum of nc and nf. So, if nc>nf the average value of the hat matrix diagonals must always be less than or equal to 1. If if nc<nf then it is possible for the mean value of all the hat diagonal elements to be 1 an a perfect fit may be made. This provides an idea as to which cases have a greater effect on the outcome and is called the leverage. This is an interesting marker because it doesn’t depend on the Y’s and only on the structure of A.

If we write the regression equation in the form:

where is a random variable with zero mean and variance . Then the variance in the residuals is:

We can then create studentized residuals:

The nice thing is that like the leverage these are dimensionless. Large values are associated with problems in a given case.

Another quantitative measure of the influence of a data point on the regression is Cooks’s distance which is the difference in the residuals when the ith case is deleted divided by the mean sum of squares of the residuals without the ith case being deleted. Large values are then values that have a great deal of influence on the regression.

This makes sense only when nc>nf in the other cases where there may be no error in the fit because there are a large number of explanatory variables. D is approximately F distributed an using a cutoff of 1 for influential results is typical.

This is not generally useful if there are many more factors than cases as the residuals are generally zero. And it is useful to know how important each case is our main issue is with the variables and knowing how important each variable is. The next section outlines a way to figure that out.

3.5.1-Transformation and normalization of the problem

Another transformation of the problem can provide important physical insights and lead to potentially useful markers of association. If both sides of the equation are multiplied by the non-singular diagonal matrix:

and X’ is defined by:

the resulting equation is:

where:

And:

This transformation allows many useful insights into the linear equation problem. First, S is the vector of the correlations between each explanatory variable and the outcome variable and is hence always between -1 and 1. R is the matrix of correlations between each of the explanatory variables. Every element in R is between -1 and 1 and the main diagonal of R has 1 for all of its elements. Thus, R becomes the identity matrix when the explanatory variables are uncorrelated so that X’=S in this case. In addition, all of the variables S, R, and X’ are dimensionless. A useful consequence of (1.42) is that when the explanatory variables are uncorrelated and R is the identity matrix then:

The meaning of this is clear. If all of the are uncorrelated then are a set of unit basis vectors which span a space of dimension nF. Thus the elements in the sum are the squares of the projections of Y onto each basis vector. As long as then so that and the residual sum will be zero.

This process identifies the elements of S as indicators of the strength of the relation between the outcome and explanatory variables. It also suggests that X’ is also a possible marker at least when R is invertible.

3.5.2-A Special Case

The simple structure of the matrix R makes it possible to consider certain special cases that provide additional insight into the solutions. In the case where R takes the form:

where J is a matrix in which every element takes on the value 1. For example, when nF=7, this matrix has the form:

It is easy to see that when a=1, R is the identity matrix (so that all explanatory variables are uncorrelated) and when a=0 all of the explanatory factors are perfectly correlated. Thus, a is a measure of the degree of correlation between explanatory factors. The determinant of this matrix is: and its inverse R-1 has a simple form with all diagonal elements equal to and all the off diagonal elements equal to **:**

And thus:

More generally, if the order of the explanatory variables can be chosen so that R has a block diagonal form with q blocks:

Then if (i,j) is the I’th variable in the j’th block then:

Where nj is the number of variables in the j’th square block and aj is the value of a in the j’th block and is the sum of all the S’s in block j. Thus the mean value of X’ in each block is:

(1.50) and (1.51) imply that as correlations between variables increases and a approaches 0 (perfect correlation):

Thus as correlations within a block increase, the values of the individual X’s vary greatly while the mean value has a stable solution. This suggests that in the presence of highly correlated groups of explanatory variables the mean value of X rather than the individual values is important.

So far we have explored means of identifying variables with a high likelihood of being related to the outcome variables. Another approach is the addition of external constraints that remove the problem with multiple solutions.

3.6 Solutions

3.6.1-Clustering

Reducing the effective number of variables by clustering similar variables into a larger variable is one method of reducing the problems that are associated with large data sets. The question is how to rationally cluster variables in such a way as to not bias results. The analyses in the first part of this appendix show that the clustering method is critical to being able to detect variables that are associated with another variable. Two problems were evident, First, clustering variables based only on their mathematical similarity to one another may put together variables with very different relations to the dependent variable and hence obscure important effects. Second, purely mathematical clustering methods produce output variables of questionable biological significance and so results are difficult to interpret. Second, clustering variables after data analysis may bias results and so that should be avoided.

For the future the key is to find an a priori clustering scheme that associates variables based on a biologic similarity as well as according to their associations with the dependent variable.

3.6.2-Regularizer

One approach is to acknowledge that the explanatory variables are measured and hence have intrinsic uncertainty. This means that any solution to must provide relatively good solutions for a number of other related problems which could occur as A takes on different values. In particular if there are Q different explanatory data matrices that might likely arise out of slightly different measurements, then one criterion for selecting the best value is to minimize the sum of squared errors over each different value of A.

For the sake of simplicity, let:

In this case, X is determined from:

where the second equation is true only when the matrix C+D is invertible. Using the definitions of the explanatory variables that make up the matrix A where aij are the elements of A, it is possible to write (the data in the j’th column of ) and so:

In the case where the are not correlated then D becomes a diagonal matrix:

with entries on the main diagonal representing the expected variance in each variable. The experimental variations in each of the variables may uncorrelated even if the variables are not. For example in case of the Family Food database, errors due to miscoding might be uncorrelated.

The importance of this is that since C has non-negative eigenvalues, if D is diagonal and has all positive elements, the matrix C+D will always be invertible. So, D takes the form of a traditional regularizer in traditional ill-posed problems. The difficulty with this approach is determining the actual value of the uncertainties in each variable since these will have a huge effect on the results of the regression.

**4.0-A Short Illustrated Tutorial on the Distribution of p-values**

A common problem encountered in clinical studies is determining the chance that two sets of data are drawn from the same population. Mathematically, if there are two groups of data labelled then the problem is to compute p, the probability of observing both of these data sets under the null hypothesis that they were drawn from the same distribution. Many statistical tests can be devised to try to calculate this probability. One choice is to ask whether the mean values of the data in are the same. If the distribution of the measurements in each group is approximately Gaussian, then the t-test results. Another choice might be to compare the rank ordering of the data points in the two groups, resulting in a test like the Mann-Whitney test. No matter what specific test is applied, the testing follows the pattern outlined in Figure T1 where the data from the first group are shown in red and the data from the second group are shown in blue. Now, consider the situation shown in Figure T2 where the exact probability distribution of the two samples is known and the statistical test is applied to multiple samples randomly taken from each distribution. As shown in that figure, this will lead to a probability distribution of p-values. If in an experiment samples taken from happened to be larger and the samples from were smaller, the computed p-values would be near 1 and if the samples taken from were smaller and the samples taken from happened to be larger the p-values would be small.

Mathematically, statistical testing involves taking the sample of data and replacing it with a statistic t(k) , which, if the are drawn from a known probability distribution, will also have a probability distribution given by say f(t). Look at the probability distribution of the cumulative density function for t, for :

where is the traditional p-value, the probability that the cumulative density function of for the measured value of t is less than the threshold T. Note that the cumulative density function F is invertible as long as the distribution is continuous without any zeroes. This shows that the p-values must be uniformly distributed on [0,1] in this case. This is independent of the actual distribution for t. This means that if the distribution of p-values is not uniform then the samples drawn do not come from the proposed probability distribution.

It is useful to illustrate this graphically as in the simulations shown in Figure T3 when 100 data points are randomly chosen from two normal distributions with standard deviation of 1 and differing mean values with Student’s t-test used to compare the samples. Figure T4 shows the results of testing the same data using the Mann-Whitney test to compare the samples. Similar results are found using either statistical test. Figure T5 demonstrates that using the t-test when the underlying distribution is lognormal which is very different than the normal distribution yields similar results. Figure T6 illustrates different patterns that may be seen in the p-value cumulative density plots.

In the specific case of the study discussed in this paper, the null hypothesis is that the there are no relationships between any of the explanatory variables and the dementia outcome variable. Thus, by the same arguments as adduced above, the distribution of p-values describing the relationship between each explanatory variable and the outcome variable would be uniformly distributed. Significant deviations of the p-value distribution from the uniform distribution indicate that there is a significant relationship.

**5.0-Tables**

**Table. S1** The effects of normally distributed random noise and the criterion for selecting variables as significant in the univariate analysis on the true positive detection rate (TPR) and the false positive discovery rate (FDR). The outcome variable was entirely determined only by the value of variable #203 and the random noise (frax is the ratio of the noise variance to the variance in variable #203).

| Noise Level  (frax) | Spearman p FDR Selection Criterion | TPR Univariate | TPR Backward  Stepwise  Regression | TPR Forward  Stepwise  Regression | FPR  Univariate | FPR  Backward  Stepwise  Regression | FPR  Forward  Stepwise  Regression |
| --- | --- | --- | --- | --- | --- | --- | --- |
| 2.0 | 0.05 | 0.05 | 0 | 0 | 0.007 | .0007 | 0.0023 |
| 1.0 | 0.05 | 0.3 | 0 | 0.25 | 0.014 | 0.0016 | 0.0025 |
| 0.6 | 0.05 | 0.85 | 0 | 0.8 | 0.03 | 0.0035 | 0.002 |
| 0.3 | 0.05 | 1 | 0 | 0.95 | 0.12 | 0.01 | 0.003 |
| 0.1 | 0.05 | 0.95 | 0 | 0.95 | 0.21 | 0.01 | 0.002 |
| 2.0 | 0.001 | 0 | 0 | 0 | 0.007 | 0.0006 | 0.0025 |
| 1.0 | 0.001 | 0.14 | 0 | 0.11 | 0.01 | 0.001 | 0.0028 |
| 0.6 | 0.001 | 0.9 | 0 | 0.7 | 0.014 | 0.001 | 0.002 |
| 0.3 | 0.001 | 1.0 | 0 | 1.0 | 0.022 | 0.0002 | 0.0014 |
| 0.1 | 0.001 | 0.97 | 0 | 0.97 | 0.15 | 0.01 | 0.002 |

**Table. S2** The effects of normally distributed random noise and the criterion for selecting variables as significant in the univariate analysis on the true positive detection rate (TPR) and the false positive discovery rate (FDR). The outcome variable was entirely determined by an equally weighted standardized sum of the independent variables: #103,203,303,403,503 203 and the random noise (frax is the ratio of the noise variance to the variance in the sum of the variables).

| Noise Level  (frax) | Spearman p FDR Selection Criterion | Variable | TPR Univariate | TPR Backward  Stepwise  Regression | TPR Forward  Stepwise  Regression | FPR  Univariate | FPR  Backward  Stepwise  Regression | FPR  Forward  Stepwise  Regression |
| --- | --- | --- | --- | --- | --- | --- | --- | --- |
| 0.3 | .001 |  |  |  |  | .013 | .0017 | .003 |
| 0.3 | .001 | 103 | 0.2 | 0 | .15 |  |  |  |
| 0.3 | .001 | 203 | 0 | 0 | 0 |  |  |  |
| 0.3 | .001 | 303 | 0.1 | 0 | .1 |  |  |  |
| 0.3 | .001 | 403 | 0.1 | 0 | .15 |  |  |  |
| 0.3 | .001 | 503 | 0.3 | 0 | .3 |  |  |  |
| 0.3 | .05 |  |  |  |  | .011 | .002 | .0028 |
| 0.3 | .05 | 103 | 0.3 | 0 | 0.2 |  |  |  |
| 0.3 | .05 | 203 | 0 | 0 | 0 |  |  |  |
| 0.3 | .05 | 303 | 0.05 | 0 | 0.05 |  |  |  |
| 0.3 | .05 | 403 | 0.1 | 0 | 0.05 |  |  |  |
| 0.3 | .05 | 503 | 0.45 | 0 | .45 |  |  |  |
| 0.1 | .001 |  |  |  |  | .039 | .00019 | .00086 |
| 0.1 | .001 | 103 | .75 | 0 | .6 |  |  |  |
| 0.1 | .001 | 203 | 0 | 0 | 0 |  |  |  |
| 0.1 | .001 | 303 | .55 | 0 | .2 |  |  |  |
| 0.1 | .001 | 403 | .65 | 0 | .3 |  |  |  |
| 0.1 | .001 | 503 | 1 | 0 | 1 |  |  |  |

**Table S3**.—Summary of global tests of association using the distribution of p-values in each group. The p-values were taken from the Spearman rank correlation test. All indicates that all data from each time point is used. In this table, “averaged” means that all of the data from different time periods was averaged and the only cases were the different regions of England. KS=Kolmogorov-Smirnov test. AD=Anderson-Darling test.

| Data | Group | KS Actual-Uniform | AD Actual-Uniform | KS Actual-Permutation | AD Actual-Permutation |
| --- | --- | --- | --- | --- | --- |
| All | All | <0.0000001 | <0.0000001 | <0.0000001 | <0.0000001 |
| All | Demographics | <0.0000001 | <0.0000001 | <0.0000001 | <0.0000001 |
| All | Food | <0.0000001 | <0.0000001 | <0.0000001 | <0.0000001 |
| Averaged | All | <0.0000001 | <0.0000001 | <0.0000001 | <0.0000001 |
| Averaged | Demographics | <0.0000001 | <0.0000001 | <0.0000001 | <0.0000001 |
| Averaged | Food | <0.0000001 | <0.0000001 | <0.0000001 | <0.0000001 |

**Table S4a**.—Summary of global tests of association using the R2 test. The sum of the square of all Spearman rank correlations from every variable in each group was computed and the probability that the values obtained after permutation of the dependent variable exceeded this value was computed. All indicates that all data from each time point is used. Averaged means that all of the data from different time periods was averaged (There are only 9 cases in the averaged set and 72 in the full data set.)

| Data | Group | Probability that permutation of the dependent variable yields a value of  R2 greater than that in the actual data. |
| --- | --- | --- |
| All | All | 0 (out of 200) |
| All | Demographics | 0 (out of 200) |
| All | Food | 0 (out of 200) |
| Averaged | All | 0.065 (out of 200) |
| Averaged | Demographics | 0 (out of 200) |
| Averaged | Food | 0.085 (out of 200) |

**Table S4b**.—Summary of global tests of association using the R2 test. Unlike the data in Table S4a, the Spearman R values are those obtained after the effects of the covariates are removed from both the independent and dependent variable. All indicates that all data from each time point is used. Averaged means that all of the data from different time periods was averaged.

| Data | Group | Probability that permutation of the dependent variable yields a value of  R2 greater than that in the actual data. |
| --- | --- | --- |
| All | All | 0 (out of 200) |
| All | Demographics | 0 (out of 200) |
| All | Food | 0 (out of 200) |
| Averaged | All | 0.405 (out of 200) |
| Averaged | Demographics | 0.1 (out of 200) |
| Averaged | Food | 0.445 (out of 200) |

**Table S5.** Estimates of concurrence between the univariate tests of association. The specific question being answered is if the p-value generated by the Spearman rank correlation is less than pc what is the fraction of univariate tests also yield a p-value less than pc. This analysis overestimates the degree of discordance as a test that yields p=0.0001 in the Spearman test and p=0.001 in the Pearson test would be marked as discordant even though both may be statistically significant.

| pc | Fraction of tests concurring |
| --- | --- |
| 0.00001 | 0.95 |
| 0.0001 | 0.93 |
| 0.0005 | 0.91 |
| 0.001 | 0.89 |
| 0.005 | 0.84 |
| 0.01 | 0.82 |
| 0.05 | 0.77 |

**Table S6.** Effects of the various selection criteria on the number of variables selected. The first six criteria are applied to the full data set and the last is applied to the averaged data set. Note that the number of variables excluded by the permutation tests may vary from one analysis to another slightly.

| Criterion | Name | #Variables Before | #Variables After |
| --- | --- | --- | --- |
| 1 | Spearman p-FDR<0.05 | 519 | 55 |
| 2 | Spearman Permutation<0.05 | 55 | 55 |
| 3 | Pearson p<0.05 | 55 | 55 |
| 4 | Min Cross-Tab Table p<0.05 | 55 | 55 |
| 5 | Partial Correlation p<0.05 | 55 | 35 |
| 6 | Partial Correlation Permutation<0.05 | 35 | 33 |
| 7 | Averaged Data Spearman p<0.05 | 33 | 13 |

**Table S7.** Some results of the univariate testing. 20 variables with the lowest Spearman p values describing its relationship to the dementia variable. Variables 3 and 8 are included as covariates and are entered into the model independent of the results of the partial correlation analysis. This is not a complete list. All results are in supplementary spreadsheet s 2 and 3.

| Variable  Number | Variable  Name | Spearman p | Spearman R | Partial Correlation Spearman p | Partial Correlation Spearman R |
| --- | --- | --- | --- | --- | --- |
| 3 | Fraction 65+ | 2.66E-17 | 0.801724 | -- | -- |
| 250 | Other cereal convenience foods | 1.6E-13 | 0.736832 | 8.12E-06 | 0.499035 |
| 255 | Other cereal foods - frozen and not frozen | 4.81E-13 | 0.726992 | 7.22E-09 | 0.618175 |
| 78 | Other convenience meat products - frozen or not frozen | 2.63E-12 | 0.710882 | 0.000424 | 0.404528 |
| 240 | Cooked rice | 5.61E-12 | 0.703357 | 6.19E-13 | 0.724677 |
| 14 | Stroke: QOF prevalence (all ages) | 3.96E-09 | 0.626439 | 0.019085 | 0.275677 |
| 76 | Meat based ready meals and convenience meat products | 7.44E-09 | 0.617757 | 0.01568 | 0.283845 |
| 330 | Hot dogs and sausage sandwiches | 1.94E-08 | 0.604026 | 1.61E-07 | 0.571194 |
| 355 | Fresh and processed potatoes | 4.12E-08 | 0.592739 | 0.001148 | 0.375619 |
| 194 | Frozen fruit and fruit products | 4.54E-08 | 0.59126 | 5.01E-05 | 0.458968 |
| 248 | Pizzas - frozen and not frozen | 7.58E-08 | 0.583285 | 0.043899 | 0.238215 |
| 186 | Other soft fruit, fresh | 8.43E-08 | 0.581613 | 0.012607 | 0.292655 |
| 477 | Other sponge cakes or desserts (not cream cakes) | 2.76E-07 | 0.562223 | 7.99E-05 | 0.447842 |
| 301 | Champagne, sparkling wines and wine with mixer | 3.92E-07 | 0.556242 | 2.72E-06 | 0.520902 |
| 8 | White British | 4.35E-07 | 0.554412 | -- | -- |
| 287 | Soft drinks, not concentrated, low calorie | 4.48E-07 | 0.553926 | 0.003145 | 0.343398 |
| 429 | Butter and margarine eaten out | 4.5E-07 | 0.55383 | 0.012874 | 0.291819 |
| 373 | Other root vegetables or tubers e.g. turnip, parsnip, radish, beetroot | 1.47E-06 | 0.53251 | 0.000732 | 0.389028 |
| 247 | Pizza | 5.16E-06 | 0.508296 | 2.25E-07 | 0.565631 |

**Table S8**—The best (minimum AIC) order 3 regression models. This table shows the slope and p-value for the significance of the variable in the model. The AIC and regression R2 are noted.

| Variable | Model1 (slope) | Model1  (p-value) | Model2  (slope) | Model2  (p-value) |
| --- | --- | --- | --- | --- |
| 3 | 0.039 | 1.6x10-24 | .030 | 7.88x10-18 |
| 120 | -.000026 | 1.6x10-8 | -- | -- |
| 198 | -9.5x10-6 | 5x10-9 | -0.00001 | 1.0x10-9 |
| 255 | -- | --- | .00004 | 1.2x10-7 |
| AIC | -888 | | -885 | |
| R2 | .886 | | .879 | |

**Table S9**. After conducting multiple regression analyses (exhaustive regression) of up to 3 of the independent variables selected on the basis of the univariate analysis, this table gives the fraction of the lowest AIC models that include the given variable. A * indicates that the variable was also in the final forward regression model.

| Variable number | Name | Fraction of the best exhaustive regression models including this variable | Fraction of time variable appears in the cluster with the lowest p-value in the cluster regression. |
| --- | --- | --- | --- |
| 3* | Fraction 65+ | 1 | 0.5 |
| 198 | Pure fruit juices | 1 | 0.0 |
| 120* | Sugar | 0.5 | 0.15 |
| 255 | Other cereal foods - frozen and not frozen | 0.5 | 0.35 |

**Table S10**—Forward stepwise regression with p=0.05 and VIF=10 to enter or remove. Two analyses were performed one with all of the variables in the model after the univariate selection process and the second with only the variables in the exhaustive regression models. Selection criteria did not include the averaged data. In order to elucidate why some variables are in one model and not the other, Spearman rank correlations between variables in the model created from the exhaustive regression and the model resulting from the use of all variables were computed. These are in the columns marked Corr1 and Corr2. v=variable, p=p-value, R=Spearman R. Selection criteria did NOT include any based on the averaged data.

| Variable | Name | Slope Parameter | p | Slope Variance | Variance Inflation Factor | AIC/Corr1 | R2/Corr2 |
| --- | --- | --- | --- | --- | --- | --- | --- |
| All Variables in the After the Univariate Selection | | |  |  |  | -948 | 0.957 |
| 3 | Fraction 65+ | 0.029 | 4.8x10-22 | .0019 | 1.9 |  |  |
| 120 | Sugar | -,000015 | 2.6x10-6 | 2.9x10-6 | 1.6 |  |  |
| 190 | Processed fruit and fruit products | -5.73x10-6 | 5.3x10-9 | 8.5x10-7 | 1.6 |  |  |
| 301 | Champagne, sparkling wines and wine with mixer | 0.000019 | 0.0014 | 5.7x10-6 | 1.9 |  |  |
| 330 | Hot dogs and sausage sandwiches | .00014 | .00027 | .000036 | 1.6 |  |  |
| 373 | Other root vegetables or tubers e.g. turnip, parsnip, radish, beetroot | .00038 | .005 | .00013 | 1.4 |  |  |
| 410 | Other savoury or sweet sandwiches | -0.000064 | 1.5x10-7 | 0.000018 | 1.4 |  |  |
| 477 | Other sponge cakes or desserts (not cream cakes) | .00019 | .001 | .00006 | 1.8 |  |  |
| All Variables In the Exhaustive Regression | | |  |  |  | -881 | 0.9 |
| 198 | Pure Fruit Juice | -8.8x10-6 | 1.3x10-8 | 1.3x10-6 | 1.5 | v:330  p:2.3x10-8  R:-0.601 | v:190  p:2.6x10-30  R:0.956 |
| 3 | Fraction 65+ | 0.0348 | 1.1x10-19 | 0.0027 | 1.6 | v:373  p:2.4x10-10  R:0.66 | v:477  p: .00003  R:0.47 |
| 255 | Other cereal foods - frozen and not frozen | .000024 | .005 | 8.1x10-6 | 2.4 | v:373  p:9.3x10-7  R:0.54 | v:301  p:2.6x10-10  R:0.66 |
| 120 | Sugar | -.000018 | .0006 | 4.9x10-6 | 2.0 | v:301  p:1.2x10-6  R:-0.53 | v:190  p:0.0006  R:0.40 |

**Table S11**—Forward Stepwise Multiple Regression with p=0.05 and VIF<10 to enter. Two analyses were performed one with all of the variables in the model after the univariate selection process and the second with only the variables in the exhaustive regression models. Selection criteria included Spearman R in the averaged data <0.05. This is the difference between the data in this table and in S10.

| Variable | Name | Slope Parameter | p | Slope Variance | Variance Inflation Factor | AIC/Corr1 | R2/Corr2 |
| --- | --- | --- | --- | --- | --- | --- | --- |
| All Variables in the After the Univariate Selection | | |  |  |  | -875 | 0.87 |
| 2 | Fraction Female | -.167 | .0001 | .04 | 1.7 |  |  |
| 3 | Fraction 65+ | 0.039 | 2.2x10-16 | .004 | 2.1 |  |  |
| 240 | Cooked rice | 0.00009 | 1.1x10-10 | 0.00001 | 1.2 |  |  |
| 355 | Fresh and processed potatoes-eaten out | 0.000023 | .01 | 8.8x10-6 | 1.5 | v:198  p:0.00001  R:-0.44 | v:3  p:0.00004  R:0.47 |
| 359 | Potatoes - mashed | .00017 | .02 | .00007 | 1.3 |  |  |
| All Variables In the Exhaustive Regression | | |  |  |  | -870 | 0.86 |
| 240 | Cooked rice | 0.000095 | 1.25x10-10 | 0.000012 | 1.22 | v:198  p:7.4x10-8  R:-0.58 | v:120  p:2.0x10-9  R:-0.64 |
| 3 | Fraction 65+ | 0.042 | 3.4x10-18 | 0.0035 | 1.9 | v:373  p:2.4x10-10  R:0.66 | v:477  p: .00003  R:0.47 |
| 359 | Potatoes - mashed | .00022 | 0.003 | 0.00007 | 1.8 | v:120  p:0.0098  R:-0.30 | v:255  p:0.0017  R:-0.36 |
| 2 | Fraction Female | -.165 | 0.0003 | 0.04 | 1.7 | v:3  p:0.0006  R:-0.40 |  |

**Table S12.** Outcome of 20 simulations where normally distributed noise is added to the measured dependent variable. The forward multivariable model referred to in this table is that one which begins with all of the variables considered significant after the univariate testing. The false positive rate is computed as the rate at which any non-target variable appears in the model.

| Target Variable | Probability of significance in univariate model | Probability of being included in the forward multivariable model | Probability of being included in one of the exhaustive regression models |
| --- | --- | --- | --- |
| Variables in the forward stepwise regression using all significant variables in the univariate testing | | | |
| 3 | 1 | 1 | 1 |
| 120 | 0.65 | 0.65 | 0.5 |
| 190 | 1 | 1 | 0 |
| 301 | 1 | 1 | 0 |
| 330 | 1 | 1 | 0 |
| 373 | 1 | 0.65 | 0 |
| 410 | 1 | 1 | 0 |
| 477 | 1 | 0.65 | 0 |
| Average # Non Target  Variables in Model | 28.1 | 1.05 | 0 |
| (Average # non-target variables in model)/total number of variables | 0.054039 | 0.002019 | 0 |
| Variables in the forward stepwise regression using only variables in the exhaustive regression models | | | |
| 3 | 1 | 1 | 1 |
| 198 | 1 | 0 | 1 |
| 120 | 0.85 | .85 | .5 |
| 255 | 1 | 0 | .5 |
| Average # Non Target  Variables in Model | 32.1 | 6.15 | 0 |
| (Average # non-target variables in model)/total number of variables | 0.061 | .008 | 0 |

**Table S13**. Overall results of 20 simulations with 5 target variables (103, 203, 303, 403, and 505) equally contributing to the dependent variable with a low noise level (frax) =0.1. Method=Raw cluster according to each data vector, Method Correlation-cluster according to the Spearman R describing each variable’s relationship with the outcome variable. pth is the p value for entering or removal and vth is the threshold for the variance inflation factor.

| Characteristic | Method-Raw  pth=0.05  Vth=10.0 | Method-Correlation  pth=0.05  Vth=10.0 | Method-Correlation  pth=0.001  Vth=2.0 |
| --- | --- | --- | --- |
| Mean # elements in cluster with largest slope | 151 | 6.85 | 22.5 |
| Mean # elements in cluster with lowest p-value | 62.7 | 4.0 | 22.5 |
| Criterion-Significant Cluster  False Positive Rate | 0.34 | 0.045 | 0.047 |
| Criterion-Significant Cluster  True Positive Rate | 0.36 | 0.62 | 0.62 |
| Criterion-Largest Slope Cluster  False Positive Rate | 0.29 | 0.008 | 0.032 |
| Criterion- Largest Slope Cluster  True Positive Rate | 0.003 | .38 | 0.74 |
| Criterion-Smallest p-value Cluster  False Positive Rate | 0.12 | 0.003 | 0.032 |
| Criterion- Smallest p-value Cluster  True Positive Rate | 0.001 | .51 | 0.74 |
| R2 for regression analysis | .40 | 0.80 | 0.64 |

**Table S14.** Results of 20 simulations with 5 target variables (103, 203, 303, 403, and 505) equally contributing to the dependent variable with a low noise level (frax) =0.1. Criteria refer to the p-value threshold for entering or removing a variable from the forward stepwise regression or the threshold for the variance inflation factor.

| Method | Criteria | Variable | Probability of Target Variable found in Significant Cluster | Probability of Target Variable found in Cluster with largest slope | Probability of Target Variable found in Cluster with smallest p |
| --- | --- | --- | --- | --- | --- |
| Correlation | pth=0.05  Vth=10 | 103 | 0.95 | 0.6 | 0.75 |
| Correlation | pth=0.05  Vth=10 | 203 | 0.1 | 0 | 0 |
| Correlation | pth=0.05  Vth=10 | 303 | 0.9 | 0.35 | 0.4 |
| Correlation | pth=0.05  Vth=10 | 403 | 0.65 | 0 | 0.05 |
| Correlation | pth=0.05  Vth=10 | 503 | 0.5 | 0.05 | 0.2 |
| Correlation | pth=0.001  Vth=2 | 103 | 0.9 | 0.9 | 0.9 |
| Correlation | pth=0.001  Vth=2 | 203 | 0 | 0 | 0 |
| Correlation | pth=0.001  Vth=2 | 303 | 0.8 | 0.75 | 0.75 |
| Correlation | pth=0.001  Vth=2 | 403 | 0.55 | 0.5 | 0.5 |
| Correlation | pth=0.001  Vth=2 | 503 | 0.85 | 0.85 | 0.85 |
| Raw | pth=0.05  Vth=10 | 103 | 0.3 | 0.3 | 0.1 |
| Raw | pth=0.05  Vth=10 | 203 | 0.6 | 0.3 | 0.1 |
| Raw | pth=0.05  Vth=10 | 303 | 0.3 | 0.3 | 0.1 |
| Raw | pth=0.05  Vth=10 | 403 | 0.3 | 0.3 | 0.1 |
| Raw | pth=0.05  Vth=10 | 503 | 0.3 | 0.3 | 0.1 |

**Table S15.**—Forward stepwise regression results using the test data cluster method simulation with the raw and correlation clustering methods. pth=0.05, Vth=10. A * next to a variable indicates that it was a target variable. These were randomly selected from the 20 simulations for the sake of illustration.

| Method-Raw | | | | Method-Correlation | | | |
| --- | --- | --- | --- | --- | --- | --- | --- |
| Cluster | Variables | Slope | p | Cluster | Variables | Slope | p |
| 12 | 21,24,25,40,66,76,87,106,140,187,200,202,203*,206,  216,221,302,501 | -1.68 | 5.3x10-6 | 2 | 3,8,77,117,  303*,389,435,  480,494,508,  512 | 1.76 | 9.5x10-10 |
| 15 | 28,30,46,58,59,60,74,107,113,114,134,141,145,156,  173,177,183,188,189,195,196,232,268,278,279,289,  292,294,305,357,364,401,437,438,439,444,445,456,  494,495,512 | 1.95 | 3.1x10-6 | 3 | 4,5,6,7,10,238,239 | 0.726 | 0.00002 |
| 16 | 29,31,38,50,57,61,65,71,86,101,108,109,115,159,160,  201,204,209,228,230,233,234,249,252,258,307,345,  410,457,469 | 2.07 | .0009 | 6 | 15,72,75,226,  231,270,298,  306,329,330,  342,355,369,  431,479,506,  510 | -0.985 | 0.00004 |
|  |  |  |  | 21 | 46,89,120,169,170,180,198,  281,307,328,  387,395,416,  434,466,501 | -0.799 | 0.00009 |
|  |  |  |  | 29 | 103*,104,289,  296, | 0.768 | 5.3x10-9 |

**Table S16**—Regressions from a randomly selected simulation of cluster regression with the correlation clustering methods. pth=0.001, vth=2. A * next to a variable indicates that it was a target variable. This were randomly selected from the 20 simulations for the sake of illustration.

| Method-Correlation | | | |
| --- | --- | --- | --- |
| Cluster | Variables | Slope | p |
| 30 | 103*,104,300,503* | 0.747 | 1.4x10-7 |
| 32 | 117,174,289,296,301,302,303*,403*,435,512 | 0.629 | 0.00009 |

**Table S17**—Analysis of significant variable clusters using the correlation clustering followed by forward stepwise multiple regression with pth=0.05, vth=10 to enter/remove from the forward stepwise analysis. This is the best model out of all the different number of clusters specified.

| Cluster | Slope Parameter | Cluster  Size | p | Slope Variance | Variance Inflation Factor | AIC | R2 |
| --- | --- | --- | --- | --- | --- | --- | --- |
| 2 | 0.49 | 1 | 8.6x10-7 | 0.089 | 9.6 | 12.4 | 0.95 |
| 3 | 0.49 | 1 | 1.1x10-6 | 0.090 | 9.8 |  |  |
| 6 | -0.38 | 2 | 2.97x10-6 | 0.074 | 4.5 |  |  |
| 10 | 0.181 | 4 | 0.038 | 0.085 | 4.2 |  |  |
| 15 | -0.414 | 11 | 0.0001 | 0.1 | 2.9 |  |  |
| 34 | 0.187 | 6 | 0.017 | 0.07 | 4.3 |  |  |
| 37 | 0.210 | 2 | 0.00017 | 0.05 | 2.2 |  |  |
| 40 | -0.2 | 2 | 0.00079 | 0.06 | 2.4 |  |  |

**Table S18.** Some names of the components of the different clusters (CHD-Chronic heart disease CKD=chronic kidney disease). A * next to a variable name indicates that it was also found to be significant in the first analysis of univariate selection followed by forward stepwise regression.

| Cluster | Variables | Name1 | Name2 | Name3 | Other |
| --- | --- | --- | --- | --- | --- |
| 2 | 3* | 65+ |  |  |  |
| 3 | 4 | Population Density |  |  |  |
| 6 | 7,198 | Mixed Race | Pure Fruit Juices |  |  |
| 10 | 13,186,330*,429 | CKD QOF Prevalence | Other soft fruit fresh | Eating out-hot dog and sausage sandwhich | Butter and margarine |
| 15 | 23,62,120*,215,386,395,412,426,463,  464,496 | Condensed or evaporated milk | Takeaway Chicken | Sugar | Flour  Rice pasta or noodles  Yogurt and fromage frais  Polyunsaturated fats  Solid chocolate bars |
| 34 | 136,248,286,288,294,512 | Crisps and potato snacks | Pizzas frozen and not froze | Low calorie soft drinks unconcentrated | Confectionary  Alcoholic drinks  Bread |
| 37 | 194,477* | Frozen Fruit and Fruit Products | Other sponge cakes or desserts |  |  |
| 40 | 308,419 | Eating Out Indian Food | Meat Based Sauce Chile con carne/ Bolognese Sauce |  |  |

**Table S19.** Post-hoc simulations. The predicted value of the dependent variable is determined from the linear combination of the 8 variables in the forward stepwise analysis in table S10 with noise added. In the regularized regression, a variable is considered “detected” if it is in the 8 variables with the largest regression coefficient.

| Variable | Probability of selection in univariate analysis | Probability in forward stepwise regression | Probability of inclusion in exhaustive  regression | Rank in regularized regression coefficients |
| --- | --- | --- | --- | --- |
| frax=0.2—higher noise level compatible with the residuals in the forward stepwise analysis R2 | | | |  |
| 3 | 1 | 1 | 1 | 1.7 |
| 120 | 0.95 | 0.65 | .58 | 8.35 |
| 190 | 1 | 1 | 0.5 | 6.55 |
| 301 | 1 | .95 | 0.5 | 15.1 |
| 330 | 1 | .95 | 0 | 16.35 |
| 373 | 1 | .6 | 0 | 17.7 |
| 410 | 1 | 1 | 0 | 7.6 |
| 473 | 1 | .85 | 0.5 | 10.45 |
| Target # | 7.95 | 7 | 3.08 | 5.9 |
| Target % | 0.994 | 0.88 | 0.39 | 0.74 |
| Non target  Avg # | 28 | 0.9 | 0 | 4.1 |
| Non target  Probability | 0.055 | 0.0017 | 0 | 0.008 |
| frax=0.01-very low noise level much below that determined from the forward stepwise regression R2 | | | | |
| 3 | 1 | 1 | 1 | 1 |
| 120 | 1 | 1 | 0.625 | 3.95 |
| 190 | 1 | 1 | .5 | 2.5 |
| 301 | 1 | 1 | .5 | 6.85 |
| 330 | 1 | 1 | 0 | 5 |
| 373 | 1 | 1 | 0 | 8 |
| 410 | 1 | 1 | 0 | 2.55 |
| 473 | 1 | 1 | 0 | 6.15 |
| Target # | 8 | 8 | 2.6 | 8 |
| Target % | 1.0 | 1.0 | 0.33 | 8 |
| Non target  Avg # | 32.1 | 0.15 | 0 | 0 |
| Non target  Probability | 0.067 | .000028 | 0 | 0 |

**Table S20**—Post-hoc forward stepwise multiple regression with p=0.05 and VIF=10 to enter or remove. The single food variable consisted of the standardized food variables multiplied by the Spearman R for the partial correlation residuals in the top boxes. Under the row permutation 200000 simulations were performed with different permutations of the weights under random 200000 simulations were performed with randomly chosen weights between -1 and 1. In no simulation was the regression R2 equal to or greater than that obtained with the spearman partial correlation weights. In the lower box the weights were determined from the iterative process which started with the partial correlation weights and randomly changed weights to find a better fit. The final entries report the results when only variables with the absolute value of R>0.6 are used in computing the weights

| Variable | Name | Slope Parameter | p | Slope Variance | Variance Inflation Factor | AIC | R2 |
| --- | --- | --- | --- | --- | --- | --- | --- |
| Partial Correlation Weights | | | | | | | |
| 2 | Fraction Female | -0.092 | 0.0067 | 0.033 | 1.8 | -913 | 0.92 |
| 3 | Fraction 65+ | 0.057 | 5.1x10-14 | 0.0024 | 1.6 |  |  |
| food | Weighted Food Variables | 0.00016 | 3.7x10-22 | 1.1x10-6 | 1.2 |  |  |
| Permutation |  |  |  |  |  |  | Mean:0.70  Max:0.85  Std:0.025  %>Baseline:0 |
| Random |  |  |  |  |  |  | Mean:0.70  Max:0.86  Std:0.028  %>Baseline:0 |
| Weights from the Iterative Procedure | | | | | | | |
| 2 | Fraction Female | -0.065 | 0.02 | 0.027 | 1.8 | -942 | 0.946 |
| 3 | Fraction 65+ | 0.048 | 4.21x10-35 | 0.002 | 1.7 |  |  |
| 4 | Weighted Food Variables | 0.000013 | 3.67x10-28 | 6.95x10-7 | 1.1 |  |  |
| Partial Correlation Weights—R threshold 0.6 | | | | | | | |
| 2 | Fraction Female | -0.085 | 0.019 | 0.035 | 1.8 | -904.2 | 0.908 |
| 3 | Fraction 65+ | 0.052 | 3.8x10-30 | 0.0026 | 1.64 |  |  |
| 4 | Weighted Food Variables | 0.00011 | 2.5x10-20 | 8.2x10-6 | 1.12 |  |  |
| Weights from Iterative Procedure—R threshold 0.6 | | | | | | | |
| 2 | Fraction Female | -0.072 | 0.022 | 0.031 | 1.8 | -924 | 0.930 |
| 3 | Fraction 65+ | 0.047 | 2.1x10-30 | 0.0023 | 1.7 |  |  |
| 4 | Weighted Food Variables | 0.00023 | 2.6x10-24 | 0.000015 | 1.1 |  |  |

**Table S20(b).** List of the variables and weights for the food index derived by selecting only the variables with a partial correlation with a magnitude greater than 0.6. Note that the Spearman correlation coefficient for the iteratively weighted food variable with dementia prevalence is 0.56 which is less than that of some of the raw variables.

| # | Variable Name | Spearman  p | Spearman R | Partial Correlation Spearman p | Partial Correlation Spearman R | Iterative Weight  End | Iterative Weight  Start |
| --- | --- | --- | --- | --- | --- | --- | --- |
| 240 | Cooked rice | 5.6E-12 | 0.70 | 6.2E-13 | 0.72 | 0.60 | 0.72 |
| 255 | Other cereal foods - frozen and not frozen | 4.8E-13 | 0.73 | 7.2E-09 | 0.62 | 0.35 | 0.62 |
| 119 | Sugar and preserves | 0.001 | -0.38 | 6.8E-15 | -0.76 | -0.0031 | -0.76 |
| 498 | Carbohydrates | 0.15 | -0.17 | 7.8E-09 | -0.62 | -0.0034 | -0.62 |
| 115 | Reduced fat spreads | 0.12 | -0.19 | 5.2E-09 | -0.62 | -0.0059 | -0.62 |
| 500 | Non-Milk Extrinsic Sugars | 0.13 | -0.18 | 1.6E-15 | -0.77 | -0.0096 | -0.77 |
| 499 | Beef and veal | 0.44 | -0.092 | 1.6E-13 | -0.74 | -0.0097 | -0.74 |
| 190 | Processed fruit and fruit products | 1.3E-06 | -0.54 | 9.6E-11 | -0.67 | -0.34 | -0.67 |
| 199 | Bread | 0.049 | -0.23 | 9.3E-10 | -0.65 | -0.44 | -0.65 |
| 284 | Soft drinks, concentrated (excluding low calorie) | 0.00087 | -0.38 | 5.0E-10 | -0.65 | -0.56 | -0.65 |
| 120 | Sugar | 9.2E-05 | -0.44 | 7.2E-15 | -0.76 | -0.58 | -0.76 |
| 69 | Sausages, pork, uncooked | 0.58 | 0.066 | 4.0E-10 | -0.66 | -0.70 | -0.66 |
| 198 | Pure fruit juices | 2.7E-10 | -0.66 | 3.6E-13 | -0.73 | -0.96 | -0.73 |

**Table S21.** Data sources. The first column is the data element that was to be abstracted and the second two columns provide the website where the data may be obtained for the regions of England and the entire UK. The quotation marks (“”) in the second column indicate the same data source was used for the UK and the regions of England. A dash (-) in the second column indicates that the data was not abstracted. These links were active at the time of the original submission of the manuscript.

| **Data Element** | **Regions of England** | **UK** |
| --- | --- | --- |
| ***UK Family Food* Household Purchases** | https://www.gov.uk/government/statistical-data-sets/family-food-datasets | “” |
| ***UK Family Food* Nutrient Intakes** | https://www.gov.uk/government/statistical-data-sets/family-food-datasets | “” |
| ***UK Family Food* Eating Out Purchases** | https://www.gov.uk/government/statistical-data-sets/family-food-datasets | “” |
| **Dementia**  **Prevalence** | https://fingertips.phe.org.uk/profile-group/mental-health/profile/dementia/data#page/0/gid/1938133052/pat/6/par/E12000005/ati/102/are/E08000025 | https://www.dementiastatistics.org/statistics/diagnoses-in-the-uk/  <https://fingertips.phe.org.uk/profile-group/mental-health/profile/dementia/data#page/6/gid/1938132891/pat/6/par/E12000005/ati/202/are/E08000025/cid/4/page-options/ovw-tdo-0_eng-vo-0_eng-do-0> |
| **Population**  **Characteristics** | https://www.ons.gov.uk/peoplepopulationandcommunity/populationandmigration/populationestimates/bulletins/annualmidyearpopulationestimates/mid2018/relateddata | “” |
| **Ethnicity** | https://www.ethnicity-facts-figures.service.gov.uk/uk-population-by-ethnicity/national-and-regional-populations/regional-ethnic-diversity/latest#areas-of-england-and-wales-by-ethnicity | https://www.statista.com/statistics/367842/scotland-ethnicity-of-population/  https://www.assemblyresearchmatters.org/2019/02/07/a-demographic-profile-of-northern-ireland-in-2017/ |
| **GDHI** | https://www.ons.gov.uk/economy/regionalaccounts/grossdisposablehouseholdincome/datasets/regionalgrossdisposablehouseholdincomegdhi. | “” |
| **Epilepsy** | https://www.epilepsy.org.uk/professional/epilepsy-data-visualisation/prevalence-seizure-freedom-rates-and-hospital-data | https://www.epilepsyscotland.org.uk/wp-content/uploads/2019/04/Joint_Epilepsy_Council_Prevalence_and_Incidence_September_11_3.pdf |
| **Hypertension** | https://fingertips.phe.org.uk/profile-group/mental-health/profile/dementia/data#page/3/gid/1938132859/pat/6/par/E12000009/ati/102/are/E06000022/iid/273/age/1/sex/4 | https://www.ncbi.nlm.nih.gov/pmc/articles/PMC4515998/  Bhatnagar {{2310 Bhatnagar,P. 2015;}} |
| **Obesity** | https://fingertips.phe.org.uk/profile-group/mental-health/profile/dementia/data#page/3/gid/1938132859/pat/6/par/E12000009/ati/102/are/E06000022/iid/273/age/1/sex/4. | https://www.cancerresearchuk.org/health-professional/cancer-statistics/risk/overweight-and-obesity#headingOne02/1362B_Facts%20and%20stats%20Update%20Jan%202019_LOW%20RES_EXTERNAL.pdf |
| **Diabetes** | https://fingertips.phe.org.uk/profile-group/mental-health/profile/dementia/data#page/3/gid/1938132859/pat/6/par/E12000009/ati/102/are/E06000022/iid/273/age/1/sex/4. | https://www.diabetes.org.uk/resources-s3/2019-02/1362B_Facts%20and%20stats%20Update%20Jan%202019_LOW%20RES_EXTERNAL.pdf |
| **Smoking** | https://fingertips.phe.org.uk/profile-group/mentalhealth/profile/dementia/data#page/3/gid/1938133052/pat/6/par/E12000007/ati/102/are/E09000002/iid/92443/age/168/sex/4 | https://www.ons.gov.uk/peoplepopulationandcommunity/healthandsocialcare/healthandlifeexpectancies/datasets/smokinghabitsintheukanditsconstituentcountries |
| **Inactivity** | https://www.bhf.org.uk/informationsupport/publications/statistics/physical-inactivity-report-2017 | “” |
| **Stroke** | https://fingertips.phe.org.uk/profile-group/mentalhealth/profile/dementia/data#page/3/gid/1938133052/pat/6/par/E12000007/ati/102/are/E09000002/iid/92443/age/168/sex/4 | https://www.ncbi.nlm.nih.gov/pmc/articles/PMC4515998/  Bhatnagar {{2310 Bhatnagar,P. 2015;}} |
| **Coronary Heart Disease** | https://fingertips.phe.org.uk/profile-group/mentalhealth/profile/dementia/data#page/3/gid/1938133052/pat/6/par/E12000007/ati/102/are/E09000002/iid/92443/age/168/sex/4 | - |
| **Depression** | https://fingertips.phe.org.uk/profile-group/mentalhealth/profile/dementia/data#page/3/gid/1938133052/pat/6/par/E12000007/ati/102/are/E09000002/iid/92443/age/168/sex/4 | - |

**Table S22**—Results of the univariate correlation analysis with each of the factors with the dementia prevalence. RP is the Pearson correlation coefficient and pP is the probability that there is no association by the Pearson method. pS is the probability of no association by the Spearman rank correlation test. All of the variables listed were associated with FDR<0.05. For this data, the Bonferroni level required to have a total p<.05 is pB=7.4E-05. The pP values for the first 10 variables is less than this. Only the 25 variables with the smallest pP values are shown. The category other food and drink included (mineral or spring water, baby foods, soups, spreads and dressings, pickles and sauces, jelly squares, ice cream, soy and novel protein foods among others). The superscript e indicates a factor significant with a significant relationship to epilepsy prevalence.

| **Factor** | **RP** | **pP** | **pS** |
| --- | --- | --- | --- |
| % White British Race In Population | 0.989 | 4.86E-07 | 0.00024 |
| % Mixed Race in Population | -0.985 | 1.22E-06 | 0.0011 |
| % Black Race in Population | -0.976 | 6.49E-06 | 0.0037 |
| Dried Rice Consumptione | -0.972 | 1.18E-05 | 0.00094 |
| % Other Race in Population | -0.969 | 1.66E-05 | 0.017 |
| Total Rice Consumption | -0.968 | 1.94E-05 | 0.0025 |
| Death Ratee | 0.965 | 2.44E-05 | 0.0016 |
| Birth Rate | -0.965 | 2.56E-05 | 0.0016 |
| %Asian Race in Population | -0.956 | 5.48E-05 | 0.042 |
| Mineral or Spring Waters | -0.946 | 1.17E-04 | 0.036 |
| Population density | -0.941 | 1.57E-04 | 0.0096 |
| Fraction of Population 65+ | 0.938 | 1.88E-04 | 0.013 |
| % Other White Race in Population | -0.937 | 1.97E-04 | 0.049 |
| Prevalence of Hypertensione | 0.934 | 2.27E-04 | 0.04 |
| Bacon and Ham Uncooked | 0.926 | 3.38E-04 | 0.05 |
| Salmon, fresh, chilled or frozen--total | -0.915 | 5.39E-04 | 0.0125 |
| Median Age | 0.914 | 5.55E-04 | 0.03 |
| Other Food And Drink | -0.908 | 7.11E-04 | .077* |
| Stroke Prevalencee | 0.907 | 7.25E-04 | .00391 |
| Salmon, fresh, chilled | -0.905 | 7.99E-04 | 0.0125 |
| Bacon and Ham Cooked | 0.901 | 9.25E-04 | 0.286* |
| Non-carcase meat and meat products | 0.895 | 1.12E-03 | 0.154* |
| Coronary Heart Diseasee | 0.894 | 1.17E-03 | 0.0079 |
| White fish, fresh, chilled or frozen | -0.880 | 1.74E-03 | 0.332* |

**Table S23**-Multivariate results with the prevalence of dementia as the dependent factor and the dependent variables listed. Data comes from the Regions of England only. Includes the 4 Models with lowest AIC. There were a total of 15 statistical tests performed in this group and so the p value for significance is .05/15=.003. se=standard error of the parameter estimate. vif=variance inflation factor. The F value and the p value in italics in the fifth column refers to the change in variance with adding the diet/nutritional variable.

| **Model 1** | **Variable 1** | **Variable 2** | **Variable 3** | **AIC** | **R2** |
| --- | --- | --- | --- | --- | --- |
| **1** | %Asian Race in Population | Dried Rice Consumption |  | -50.16 | 0.95 |
| **Slope**  **se**  **p**  **vif** | -0.01  0.0017  0.001*  4.3 | -0.0017  0.00022  0.0002*  4.3 |  | *F(1,5)=48*  *p=0.001** |  |
| **2** | Prevalence of Hypertension | %Asian Race in Population | 279  Dried Rice Consumption | -50.0 | 0.99 |
| **Slope**  **se**  **p**  **vif** | 0.0099  0.0092  0.33  8.8 | -0.01  0.0017  0.002*  4.3 | -0.0014  0.0003  0.011  11.1 | *F(1,4)=12.3*  *p=.024* |  |
| **3** | Coronary Heart Disease | %Asian Race in Population | 278  Total Rice Consumption | -49.4 | 0.99 |
| **Slope**  **se**  **p**  **vif** | .032  .014  .07  4.0 | -.01  .0018  .0025*  4.46 | -.0014  .0003  .007  6.62 | *F(1,4)=16.1*  *p=.016* |  |
| **4** | Coronary Heart Disease | %Asian Race in Population | 279  Dried Rice Consumption | -49.2 | .99 |
| **Slope**  **se**  **p**  **vif** | 0.013  0.017  0.47  5.8 | -0.01  0.0017  0.0022*  4.3 | -0.0015  0.00034  0.007  9.37 | *F(1,4)=15.5*  *p=0.017* |  |

**Table S24.** Correlations between the variables found significant in the first and second studies. In each block is the probability that there is no correlation according to the Spearman rank correlation test. R2 is determined by the multiple regression fit of each variable in the first study against all variables in the second study.

|  | # Significant in First Study | 238 | 239 | 240 | 263 | 93 | 56 |
| --- | --- | --- | --- | --- | --- | --- | --- |
|  | Name | All Rice | Dried Rice | Cooked Rice | Mineral Water | Salmon, Fresh chilled or frozen | Bacon and Ham, uncooked |
| # Significant  In Second Study | R2 | 0.677 | 0.694 | 0.641 | 0.611 | 0.548 | 0.707 |
| 3 | Fraction 65+ | 0.0001 | 0.0001 | 0.0003 | 0.484 | 0.713 | 0.037 |
| 120 | Sugar | 0.426 | 0.487 | <.0001 | 0.000 | 0.231 | 0.021 |
| 190 | Processed Fruit and fruit products | 0.870 | 0.244 | <.00001 | 0.470 | 0.000 | 0.465 |
| 198 | Pure fruit juice | 0.940 | 0.177 | <.00001 | 0.907 | 0.018 | 0.499 |
| 255 | Other cereal foods-frozen or not frozen | 0.378 | 0.042 | <.00001 | 0.002 | 0.632 | 0.707 |
| 301 | Champagne, sparkling wines and wine with mixer | 0.646 | 0.627 | <.00001 | 0.000 | 0.026 | 0.023 |
| 330 | Hot dogs and sausage sandwiches | 0.633 | 0.076 | <.00001 | 0.792 | 0.010 | 0.018 |
| 373 | Other root vegetables or tubers e.g. turnip, parsnip, radish, beetroot | 0.165 | 0.103 | 0.002 | 0.020 | 0.029 | 0.906 |
| 410 | Other savoury or sweet sandwiches | 0.793 | 0.195 | 0.0001 | 0.636 | 0.073 | 0.025 |
| 477 | Other sponge cakes or desserts (not cream cakes) | 0.453 | 0.325 | 0.003 | 0.007 | 0.011 | 0.221 |

**Table S25**. Comparison of index elements in the DASH, Mediterranean and MIND diets {{2440 Morris,M.C. 2015;}}. that are also in the current study. In columns 2,3,4, a + sign indicates that consuming more of the given food changes the index in such a way as to increase the risk of dementia. A – sign indicates the opposite. There are often many entries in the family food database that would be associated with the index element indicated in each diet. The values listed under current target group are the mean partial correlation coefficient and its standard deviation over all food elements that fit the Index element in the current study. To the right of this is the food variable in the group with the smallest R and the food variable with the largest R. If the results from {{2440 Morris,M.C. 2015;}} and the current study were in agreement the signs of the R value would be the same as that of the index for a given diet.

|  | Prior Study  Indices | | | Current Target Group | | Current Study  Group Extremes | | | |
| --- | --- | --- | --- | --- | --- | --- | --- | --- | --- |
| Index  Element | DASH | Med Diet | MIND | R  Mean | R  Std | R  Min | Minimum  Element | R  Max | Maximum  Element |
| Grains | - | - | - | -0.12 | 0.28 | -0.65 | Bread | 0.72 | Cooked rice |
| Vegetables | - | - |  | 0.031 | 0.22 | -0.51 | Fresh Green Vegetables | 0.39 | Other root vegetables |
| Green Leafy Vegetables |  |  | - | -0.44 | 0.076 | -0.49 | Lettuce and Leafy Salads | -0.39 | Leafy Salad Fresh |
| Potatoes |  | - |  | -0.074 | 0.28 | -0.53 | Potatoes | 0.43 | Potatoes-Mashed |
| Fruits | - | - |  | -0.10 | 0.34 | -0.73 | Pure fruit Juice | 0.46 | Frozen Fruit and fruit products |
| Dairy | - | + |  | -0.11 | 0.29 | -0.60 | Hard Cheese Cheddar type | 0.43 | Soft drinks including Milk |
| Red meats |  | + | + | -0.08 | 0.19 | -0.30 | Beef Steak-More Expensive | 0.22 | Corned Meat |
| Fish |  | - | - | 0.078 | 0.16 | -0.18 | White Fish Frozen | 0.38 | Other tinned or bottled fish |
| Poultry |  | - | - | 0.10 | 0.23 | -0.27 | Other poultry | 0.52 | Chicken burger |
| Nuts, seeds, and legumes | - | - |  | 0.065 | 0,075 | -0.046 | Nuts and Crisps | 0.11 | Nuts edible seeds and peanut butter |
| Beans |  |  | - | -0.18 | 0.32 | -0.50 | Beans, fresh | 0.19 | Other canned beans and pulses |
| Nuts |  |  | - | 0.065 | 0.075 | -0.046 | Nuts and crisps | 0.11 | Nuts edible seeds and peanut butter |
| Fast/Fried food |  |  | + |  |  |  |  |  |  |
| Total Fat | + |  |  | -0.19 | 0.26 | -0.60 | All other fats | 0.09 | Fats, preserves, sugar and custard |
| Olive oil |  | - | - | -0.4 |  |  |  |  |  |
| Butter margarine |  |  | + | 0.11 | 0.26 | -0.08 | Butter | .291 | Butter and margarine |
| Cheese |  |  | + | -0.026 | 0.30 | -0.60 | Hard cheese cheddar type | .37 | Cheese and egg dishes or pizza |
| Sweets | + |  |  | -0.085 | 0.28 | -0.40 | Chocolate coated bars and sweets | 0.33 | Boiled sweets |
| Pastries sweets |  |  | + | -0.30 | 0.27 | -0.52 | Cakes and pastries not frozen | .17 | Take away pastries |
| Sodium | + |  |  | -0.49 |  |  |  |  |  |
| Alcohol |  | + | + | 0.038 | 0.31 | -0.40 | Fortified wines | .52 | Champagne, sparkling wines and wine with mixer |

**Table S26**. Comparison of index elements in the diet inflammatory index (DII) {{2444 Shivappa,N. 2014;}} that are also in the current study. In column 2, a + sign indicates that consuming more of the given food changes the index in such a way as to increase the risk of dementia. A – sign indicates the opposite. There are often many entries in the family food database that would be associated with the index element indicated in each diet. The values listed under current target group are the mean partial correlation coefficient and its standard deviation over all food elements that fit the Index element in the current study. To the right of this is the food variable in the group with the smallest R and the food variable with the largest R.

| Variable | Diet Inflammatory  Index | R  Mean | R  Std | R  Min | Minimum  Element | R  Max | Maximum  Element |
| --- | --- | --- | --- | --- | --- | --- | --- |
| Alcohol | - | 0.038 | 0.31 | -0.40 | Fortified wines | 0.52 | Champagne, sparkling wines and wine with mixer |
| B12 | + | -0.38 |  |  |  |  |  |
| B6 | - | -0.54 |  |  |  |  |  |
| Beta carotene | - | 0.17 |  |  |  |  |  |
| Carbohydrate | + | -0.47 | 0.20 | -0.62 | Carbohydrates | -0.33 | Starch |
| Cholesterol | + | -0.24 |  |  |  |  |  |
| Energy | + | -0.46 | 0.015 | -0.48 | Energy from all sources excluding Alcohol | -0.45 | Energy all source |
| Total fat | + | -0.19 | 0.26 | -0.60 | All other fats | 0.09 | Fats, preserves, sugar and custard |
| Fibre | - | -0.32 |  |  |  |  |  |
| Garlic | - | -0.11 |  |  |  |  |  |
| Fe | + | -0.39 |  |  |  |  |  |
| Mg | - | -0.29 |  |  |  |  |  |
| MUFA | - | -0.01 |  |  |  |  |  |
| Niacin | - | 0.21 |  |  |  |  |  |
| Onion | - | -0.16 |  |  |  |  |  |
| PUFA | - | -0.025 |  |  |  |  |  |
| Riboflavin | - | -0.47 |  |  |  |  |  |
| Thiamine | - | -0.3 |  |  |  |  |  |
| Vitamin A | - | -0.26 | 0.37 | -0.56 | Retinol | 0.17 | carotene |
| Vitamin C | - | -0.24 |  |  |  |  |  |
| Vitamin D | - | -0.37 |  |  |  |  |  |
| Vitamin E | - | 0.09 |  |  |  |  |  |
| Zinc | - | -0.39 |  |  |  |  |  |
| Green/black tea | - | -0.15 | 0.37 | -0.53 | tea | 0.21 | Tea black including Chinese herbal and fruit teat |

**Table S27**—Demographic and food variables related to epilepsy with FDR<.05 in univariable analyses. The marker (eo) indicates that the given food was from the list of foods consumed “eating out”.

| **Column** | **Name** | **RP** | **PP** | **RS** | **PS** |
| --- | --- | --- | --- | --- | --- |
| 6 | Obesity | 0.96 | 3.18E-05 | 0.93 | 0.0003 |
| 477 | Other Fish Products (squid, sushi, crabsticks) (eo) | -0.96 | 3.57E-05 | -0.88 | 0.0015 |
| 10 | Coronary Heart Disease | 0.96 | 4.6E-05 | 0.90 | 0.0008 |
| 158 | Honey | -0.94 | 0.000156 | -0.83 | 0.005 |
| 239 | White Bread | 0.94 | 0.000197 | 0.83 | 0.005 |
| 283 | Infant Cereal Foods | -0.93 | 0.000251 | -0.68 | 0.04 |
| 24 | Other White Race | -0.93 | 0.000299 | -0.88 | 0.0018 |
| 8 | Prevalence of Hypertension | 0.93 | 0.000305 | 0.69 | 0.038 |
| 241 | White Bread Standard | 0.92 | 0.000356 | 0.85 | 0.0037 |
| 611 | Cheese Based Sandwhich Bread | -0.92 | 0.00038 | -0.73 | 0.025 |
| 185 | Other Fresh Green Vegetables | -0.92 | 0.000433 | -0.87 | 0.0025 |
| 223 | Fresh Stone Fruit | -0.91 | 0.000623 | -0.93 | 0.00024 |
| 7 | Stroke Prevalence | 0.91 | 0.000749 | 0.75 | 0.021 |
| 279 | Dried Rice | -0.90 | 0.000992 | -0.87 | 0.0025 |
| 17 | Death Rate | 0.90 | 0.001034 | 0.78 | 0.013 |
| 556 | Meat and Fish Soups (eo) | -0.89 | 0.001158 | -0.8 | 0.0096 |

**Table S28**-20 elements with the largest absolute value of the eigenvector of the covariance matrix with the largest eigenvalue. It is also the eigenvector which shows best correlation with the dementia prevalence. The top 20 are the same for the selection vector, M.

| **Column** | **Name** | **Value** |
| --- | --- | --- |
| 14 | Population Density | 0.294 |
| 21 | % Black Race in Population | 0.218 |
| 38 | Infant or Baby Milks-ready to drink | 0.203 |
| 25 | % Other Race in Population | 0.168 |
| 480 | Fish Burgers (in Bun)(eo) | 0.154 |
| 283 | Infant Cereal Foods | 0.147 |
| 282 | Invalid Foods, Slimming Foods and Sports Foods | 0.142 |
| 279 | Dried Rice | 0.132 |
| 127 | White Fish Dried Salted or Smoked | 0.131 |
| 24 | % Other White Race in Population | 0.129 |
| 543 | Fish Salad (Tuna, Salmon)(eo) | 0.128 |
| 432 | Chinese of Thai Buffet(eo) | 0.126 |
| 556 | Meat and Fish Soups(eo) | 0.118 |
| 511 | Other Fresh Vegetables Courgettes, Marrow aubergine, pumpkin (eo) | 0.112 |
| 611 | Cheese Based Sandwich Bread (eo) | 0.112 |
| 477 | Other Fish Products (squid, sushi etc)(eo) | 0.109 |
| 119 | White Fish Frozen | 0.107 |
| 722 | Savory Biscuits(eo) | 0.107 |
| 443 | All offal including liver kidney tongue (eo) | 0.107 |
| 206 | Dried Pulses other than air dried seed | 0.104 |

**Table S29.** The effect of number of variables and the univariate probability of association on the necessary degree of weighting for the chosen variable for the selective model to have the same true positive rate as the inclusive model.

| M | p0 | wT |
| --- | --- | --- |
| 10 | 0.1 | 6.32 |
| 100 | 0.1 | 0.0029 |
| 1000 | 0.1 | 1.94x10-43 |
| 10 | 0.01 | 105 |
| 100 | 0.01 | 58.4 |
| 1000 | 0.01 | 0.043 |
| 10 | 0.001 | 1020 |
| 100 | 0.001 | 95.2 |
| 1000 | 0.001 | 584.8 |

**Table S30**—Summary Statistics for the demographic factors used in the second study

| # | Name | Mean | Standard Deviation |
| --- | --- | --- | --- |
| 2 | Fraction of Population Female | 0.507 | 0.002 |
| 3 | Fraction 65+ | 0.18 | 0.025 |
| 4 | Population Density | 930.56 (#/km2) | 1615.56 (#/km2) |
| 5 | Fraction Population Asian | 7.13% | 4.7% |
| 6 | Fraction Population Black | 2.92% | 3.76% |
| 7 | Fraction Population Mixed | 2.07% | 1.112% |
| 8 | Fraction Population White British | 82.0% | 13.7% |
| 9 | Fraction of Population White Other | 5.0% | 3.7% |
| 10 | Other | 0.9% | 0.9% |
| 11 | Dementia QOF | 0.0069 | 0.001 |
| 12 | Chronic Heart Disease: QOF prevalence (all ages) | 0.033 | 0.006 |
| 13 | Chronic Kidney Disease: QOF prevalence (18+) | 0.045 | 0.007 |

**Table S31**. Summary statistics for some of the food variables. The letter in parenthesis next to the name gives the unit used.

| # | Name | Mean | Standard Deviation |
| --- | --- | --- | --- |
| 120 | Sugar (g) | 80.52 | 15.78 |
| 190 | Processed Fruit and fruit products (g) | 336.03 | 54.11 |
| 198 | Pure fruit juice (ml) | 251.73 | 48.26 |
| 240 | Cooked Rice (g) | 18.00 | 5.72 |
| 255 | Other cereal foods-frozen or not frozen (g) | 49.17 | 10.43 |
| 301 | Champagne, sparkling wines and wine with mixer (ml) | 17.92 | 8.75 |
| 330 | Hot dogs and sausage sandwiches (g) | 3.93 | 1.31 |
| 373 | Other root vegetables or tubers e.g. turnip, parsnip, radish, beetroot (g) | 0.53 | 0.33 |
| 410 | Other savoury or sweet sandwiches (g) | 22.26 | 4.05 |
| 477 | Other sponge cakes or desserts (not cream cakes) (g) | 4.09 | 0.90 |

**6.0-Figure Legends**

**Figures S1-S6.** Detailed block diagrams of the statistical data analysis.

**Figure S7.** Shows a histogram of p-values generated by using the Spearman rank correlation test to measure the association between each independent variable and the dependent variable for each group (a-All, b-demographic factors, c-food factors). Cumulative density function (CDF) for the p-values for each group (d-all, e-demographic factors, f-food factors). 200 random permutations were used in the permutation analysis. This is a global indicator that there is a statistically significant relation between each group of factors and dementia. All of this data is obtained using the full set of data from all of the time points.

**Figure S8**. Histograms showing the values of R2 for the actual Spearman rank correlations and those obtained after 200 random permutations of the dependent variable (dementia) for the actual data (blue) and the randomly permuted data (red). For all groups this global measure of the degree of correlation in the actual data is much higher than that seen with the random permutations. This further supports the idea that there are strong relationships between the food variables and dementia. Groups: (a) all data, (b) demographic and risk factor data and (c) food variables. All of this data is obtained using the full set of data from all of the time points. (d) shows the values of R2 for the partial correlation p-values using the full set of data. Even in this case, the values of the measured coefficients are much larger than those seen from the random permutations.

**Figure S9.** Shows cumulative density function (CDF) for the p-values for each group (a-all, b-demographic factors, c-food factors). 200 random permutations were used in the permutation analysis. This is a global indicator that there is a statistically significant relation between each group of factors and dementia. All of this data is obtained using the averaged set of data.

**Figure S10**. Histograms showing the values of R2 for the actual Spearman rank correlations and those obtained after 200 random permutations of the dependent variable (dementia) for the actual data (blue) and the randomly permuted data (red). For all groups this global measure of the degree of correlation in the actual data is much higher than that seen with the random permutations. Groups: (a) all data, (b) demographic and risk factor data and (c) food variables. All of this data is obtained using the averaged set of data.

**Figure S11.** Graphs of the univariate effect of twelve (a)-(l) variables that appear in multivariable models on dementia prevalence. The data comes from the full data set.

**Figure S12.** Graphs of the univariate effect of six (a)-(f) variables that appear in multivariable models on dementia prevalence. The data comes from the averaged data set and hence the number of cases is considerably smaller.

**Figure S13**. Plot of the residuals from the forward stepwise regression model derived from all variables significant in the univariate analysis (a) for each case. (b)Histogram of residual values. (c) Coefficients in the regularized regression after standardization for three different noise variance levels.

**Figure S14**. Correlations between the 35 independent variables identified as significant in the univariate analysis. (a) shows the p-values for the Spearman correlation analysis demonstrating that many are small and statistically significant. (b) and (c) show respectively the histogram and cumulative density function for the p-values for the off-diagonal Spearman rank correlation tests showing that they are far from linearly distributed. Thus, the low p-values are not the results of a random process.

**Figure S15**. (a)Scree plot of the eigenvalues of the covariance matrix of the 35 variables identified as significant in the univariate analyses. (b), (c) and (d) illustrations of the eigenvectors associated with the three largest eigenvalues. In this graph the “peak” variable is the variable that has the most weight in the eigenvector (positive or negative).

**Figure S16**. Plot of the relationship between the weighted food variable and Dementia QOF for (a) the partial correlation weights and (b) weights that were associated with the lowest AIC. In addition (c) shows the weights for the random noise simulations for each variable at each simulation. Simulations with lowest AIC (best model) are at the top. (d) shows the weights obtained with the iterative process in which each simulation uses the previous best set of weights. There are changes at the beginning but the weights become stable and the process converges. The small graphs to the left of the density plot are the weights for the first, 25000 iteration and last (50000) iteration. (e),(f),(g) show results when the iterative process rather than capping a weight at 1 or -1 forces no change if a proposed weight is outside the range [-1,1]. (g) shows the difference between the weights found with this process and the original partial correlation related weights.

**Figure S17**. Distributions of the Pearson p-values for the correlation between the variables and the prevalence of dementia. (a)Raw distribution (with Bonferroni threshold for p-value significance indicated), (b)Histogram of p-values (probability that the distribution is uniform is 1.4x10-23 by the Kolmogorov-Smirnov test.),(c)Cumulative Density Function (CDF) of all the observed p-values compared with the uniform distribution and the bootstrap distribution, (d) CDF for the demographic/risk factor covariates compared with the uniform distribution and the bootstrap distribution (By the Kolmogorov-Smirnov test the probability that the observed distribution is the same as the observed distribution is 6x10-14),(e) CDF for the dietary variables compared with the bootstrap and uniform distributions (By the Kolmogorov-Smirnov test the probability that the distribution of p values is the same as the bootstrap distribution is 1.8x10-19). All of the CDF’s show that there are more small p-values than expected if there were no relationship between the variables and the prevalence of dementia. First study.

**Figure S18**. Plots of four factors (a) % of population that is white British (b)dried rice consumption, (c)fraction of the population 65+ and (d)salmon consumption as a function of dementia prevalence. First study

**Figure S19.** Heat map showing the Pearson R values between all of the dietary variables and the demographics/risk factor covariates. The first row represents the correlation between these variables and dementia prevalence. The columns have been re-ordered so that the first row is in numerical order of decreasing R values. This demonstrates that overall the relationship between the dietary values and most of the covariates is strongly correlated or anti-correlated. There are substantial variations at the individual level. First study

**Figure S20.**-(a)scree plot showing that nearly all of the variance is in the first few eigenvectors. (b)Top eigenvector which contributes most to the variance has largest weights for demographic (Demo) variables and for some of the household and “eating out” dietary items but little weight for the measured nutrients. First study.

**Figure S21.** Shows the Pearson R for correlations between the variables that had DFR<0.05 in the first study

**Figure S22.** Demonstrates the correlations between variables that were significant in the first study and the second study with the forward stepwise regression model starting with all variables found significant in the univariate analyses. (a) Spearman rank correlations, (b)Spearman p values, (c) histogram of cross correlation histograms, (d) CDF of the p values recorded versus that expected if there were no correlation.

**Figure S23.** Demonstrates graphs of the relationships between the variables in the first and second studies. The variables listed on the left form the x-axis and the variables listed on the top are on the y-axis in each plot.

**Figure S24**. Plots of true positive rate vs false positive rate for the selective and inclusive models computed in 500 simulations as a function of the weights for the a priori variable selection and the noise level. 2 variables in model.

**Figure S25**. Plots of true positive rate vs false positive rate for the selective and inclusive models as a function of the weights for the a priori variable selection and the noise level. 500 simulations. 3 variables in model.

**Figure S26**. Plots of true positive rate vs false positive rate for the selective and inclusive models as a function of the weights for the a priori variable selection and the noise level. 500 simulations. 8 variables in model.

**Figure 1**. Illustration of the global tests for significance. (a) Histogram of the p-values generated by the Pearson correlation tests between each food variable and dementia in the first study. (b)The cumulative density function (CDF)describing the p-values. The bootstrap distribution is that derived by randomizing the dementia variable values and is linear as expected. The Kolmogorov-Smirnov test demonstrates that the observed distribution is unlikely to be uniform. (c) Histogram of Spearman correlation p-values for the food variables in the second study with the “full” data set. (d) the CDF of the p-values showing a strongly non-uniform shape. (e)The CDF of Spearman correlation p-values in the second study with the “averaged” data set. (f) The CDF of the partial correlation p-values with the “full” data set. Although the shape is closer to that of a uniform distribution, the Kolmogorov-Smirnov test still shows a large statistical difference.

**Figure 2**. Data from the R2 test for global significance with (a) the Spearman correlation coefficients and (b) the partial correlation coefficients for each food variable. In each case the single bar at the right represents the actual sum of R2 over all food variables. The smaller bars to the left in each graph show the results of computing the sum of the values of R2 over 200 random permutations of the data in the dementia variable.

**Figure 3**. Plot of the relationship between the food index derived by standardizing the food variables and multiplying each by their partial correlation coefficients and dementia.

**Figure T1.** Shows the flow of analysis in a typical test for a significant difference between two observed sets of data, yielding a single “p-value”.

**Figure T2.** Shows the flow of analysis when multiple data points can be sampled from two different data distributions. This leads to the possibility of multiple “p-values” one for each simulation. The result then is a statistical distribution of p-values.

**Figure T3.** Considers the situation in which 100 random samples are drawn from two normal distributions both with a variance of 1 and varying mean values. When both distributions have the same mean value as in the top row the p values resulting from a t-test are uniformly distributed on [0,1] and the cumulative density function is linearly increasing. As the difference in mean of the two normal distributions increases, the p-values resulting from the t-test are smaller and smaller and the cumulative density function for the p-values takes on a larger slope for small p-values indicating a significant effect. pKS is the probability that the given distribution of p-values is uniform according to the Kolmogorov-Smirnov test.

**Figure T4.** The same as Figure T3 except that the Mann-Whitney test is used instead of the t-test. Similar effects are noted on the distribution of p-values as seen with the t-test.

**Figure T5.** The same a Figure T3 except that each distribution is a lognormal distribution. This was chosen because it has a very different shape than the normal distribution. Again similar patterns are seen when the t-test is applied to samples taken from these two distributions.

**Figure T6.** Highlights important properties of the cumulative distribution function for the p-values in regard to whether there is a statistically significant effect. Note that only a CDF that was convex would be significant. A concave CDF might be significantly different from a uniform distribution but would not have an excess of small p-values required for statistical significance.

**7.0-Figures**

**Figure S1**

**
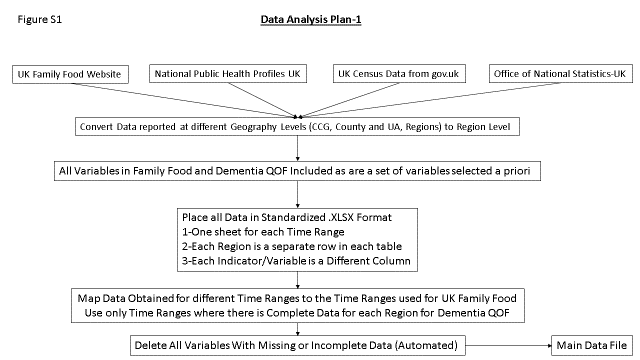
**

**Figure S2**

**
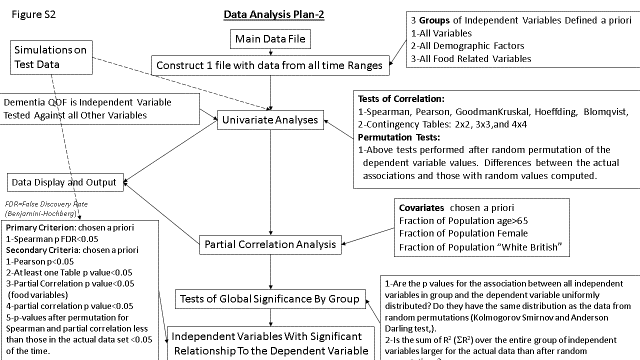
**

**Figure S3**

**
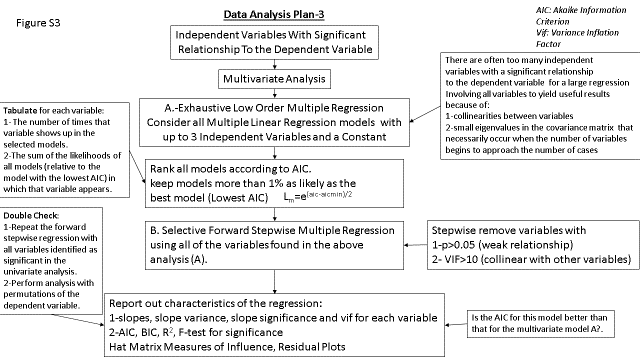
**

**Figure S4**

**
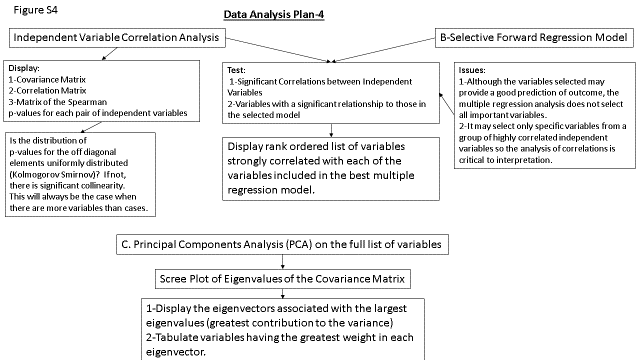
**

**Figure S5**

**
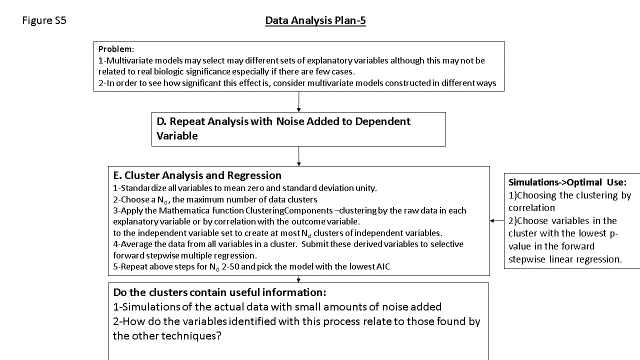
**

**Figure S6**

**
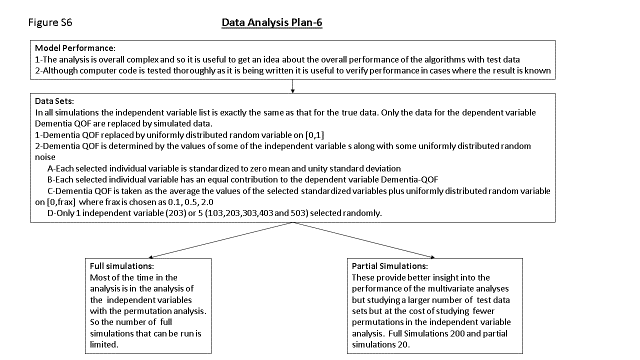
**

**Figure S7**

**
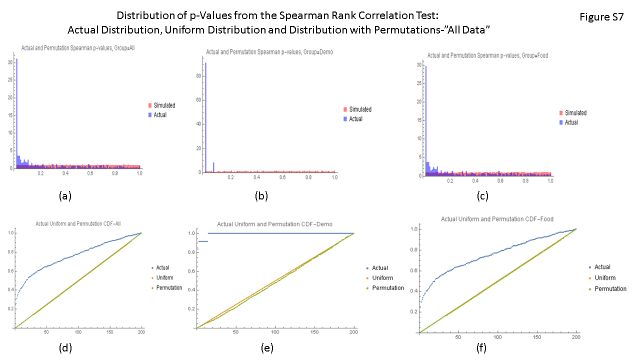
**

**Figure S8**

**
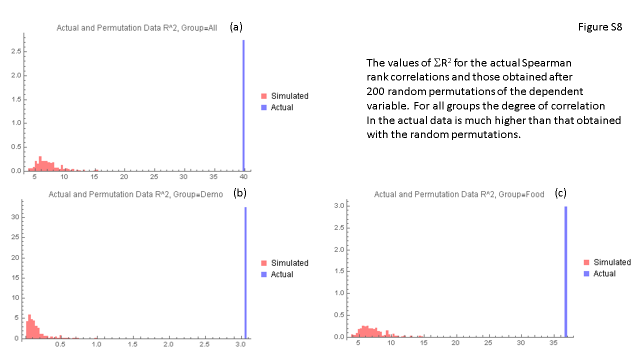
**

**Figure S9**

**
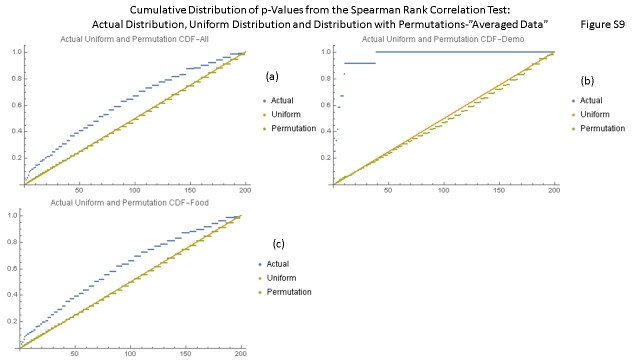
**

**Figure S10**

**
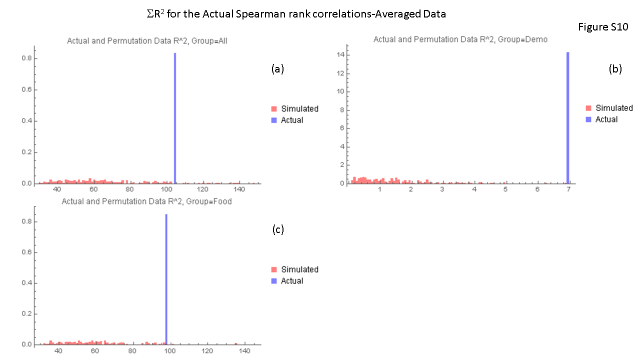
**

**Figure S11**

**
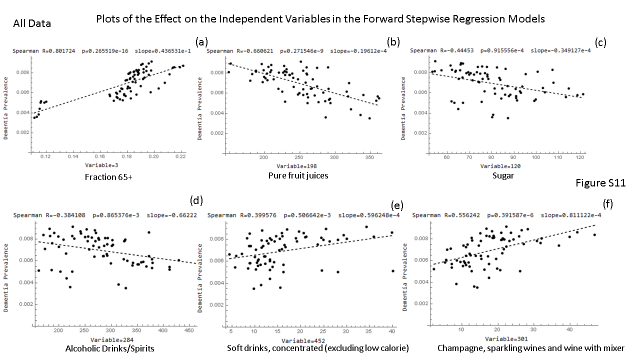
**

**
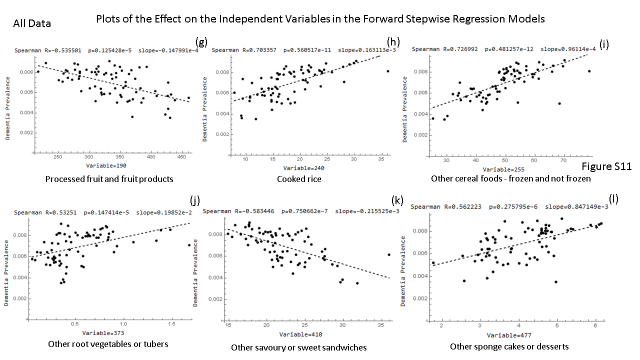
**

**Figure S12**

**
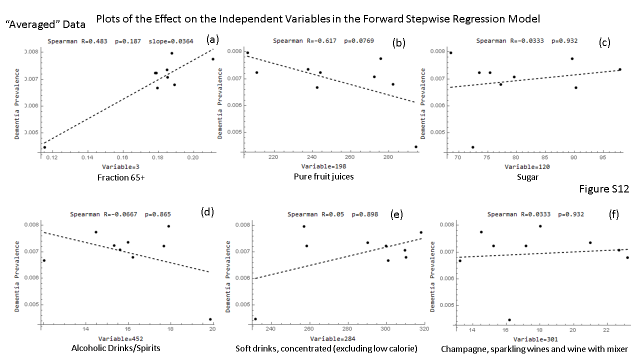
**

**Figure S13**

**
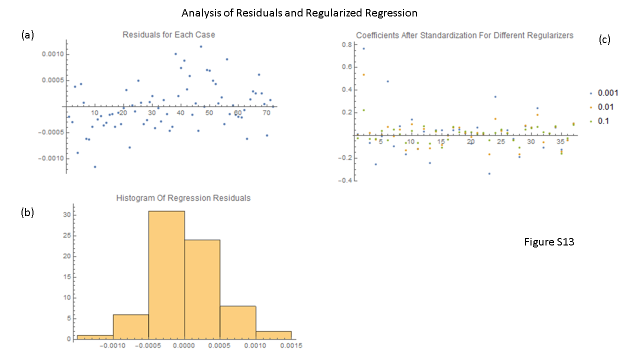
**

**Figure S14**

**
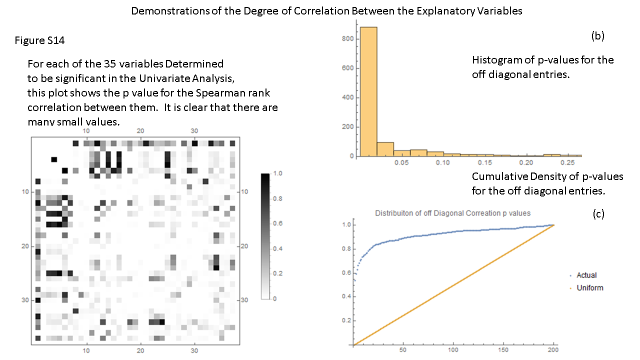
**

**Figure S15**

**
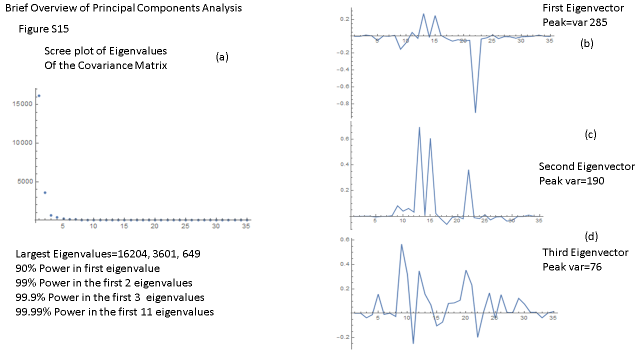
**

**Figure S16**

**
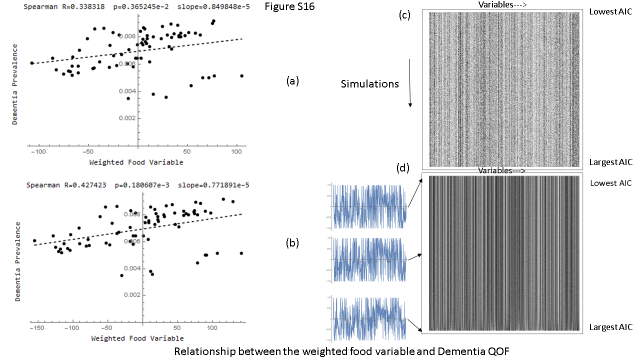
**

**
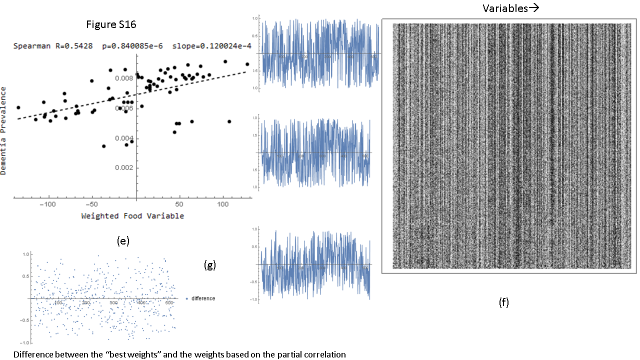
**

**
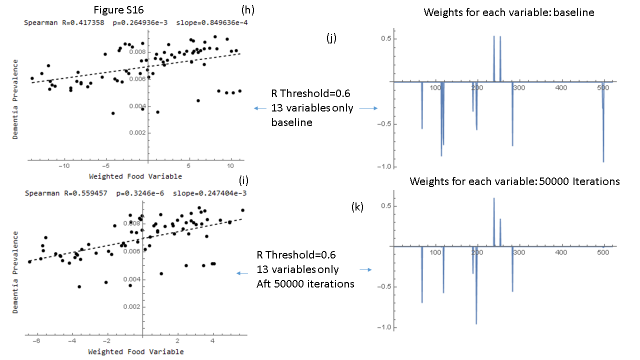
**

**Figure S17**

**
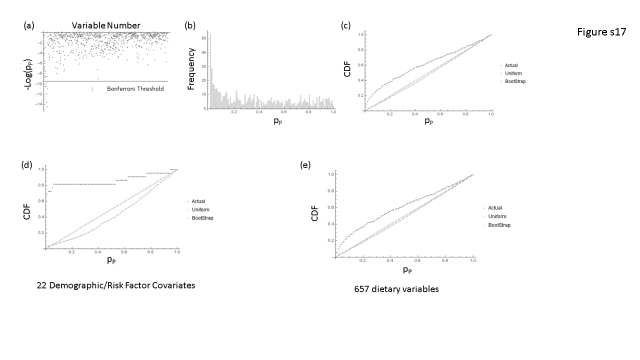
**

**Figure S18**

**
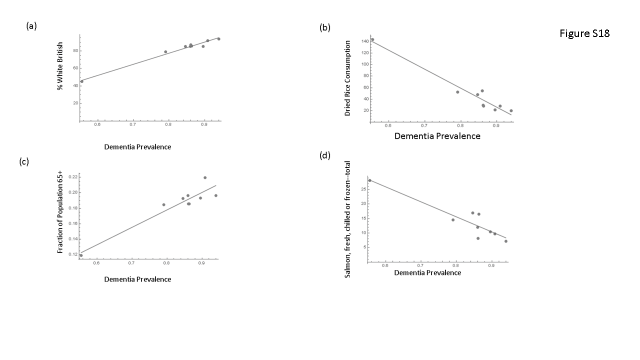
**

**Figure S19**

**
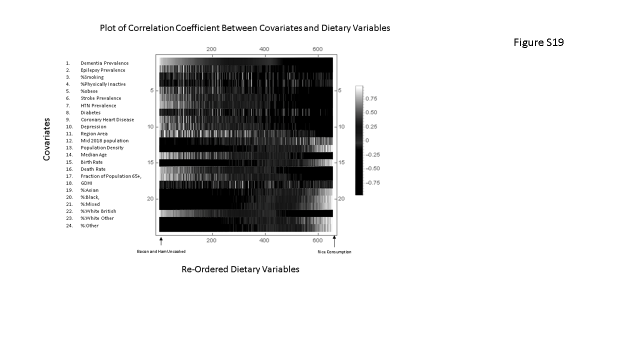
**

**Figure S20**

**
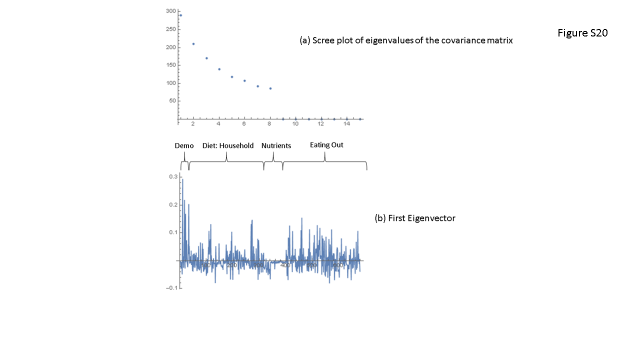
**

**Figure S21**

**
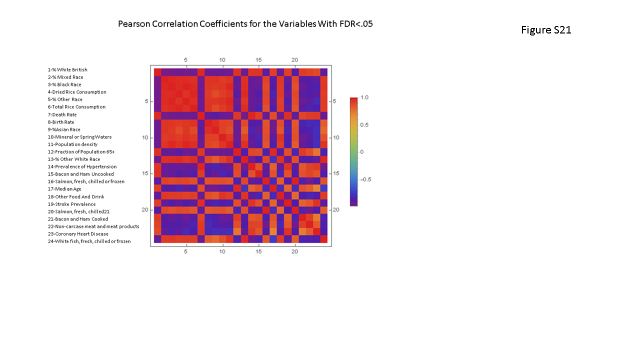
**

**Figure S22**

**
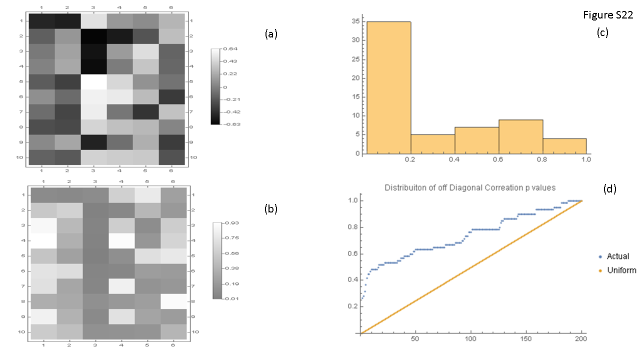
**

**Figure S23**

**
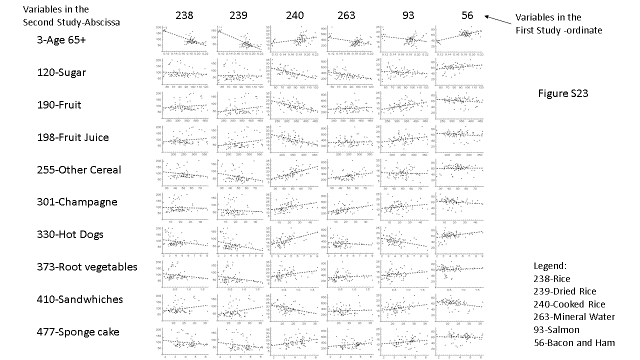
**

**Figure S24**

**
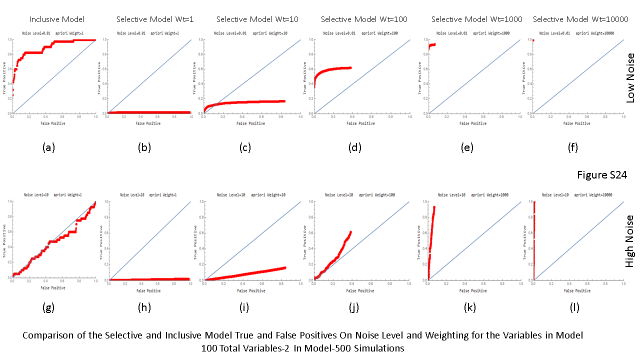
**

**Figure S25**

**
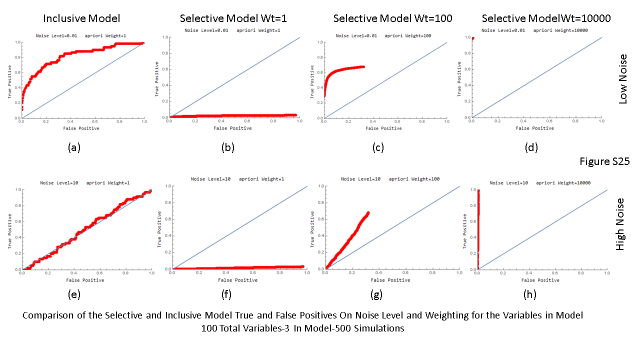
**

**Figure S26**

**
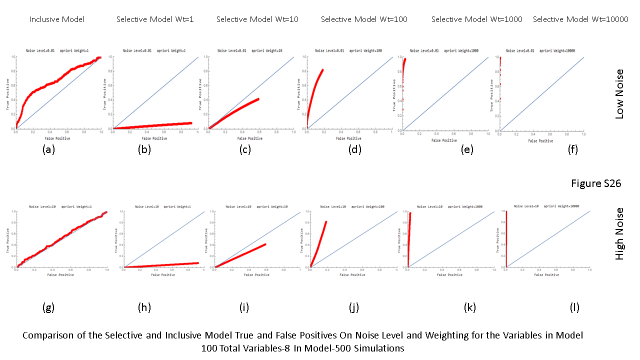
**

**Figure T1**


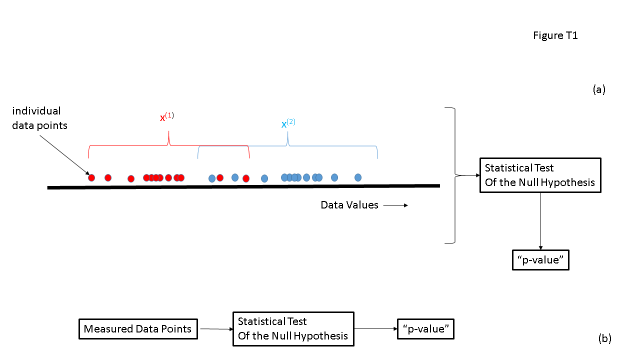


**Figure T2**


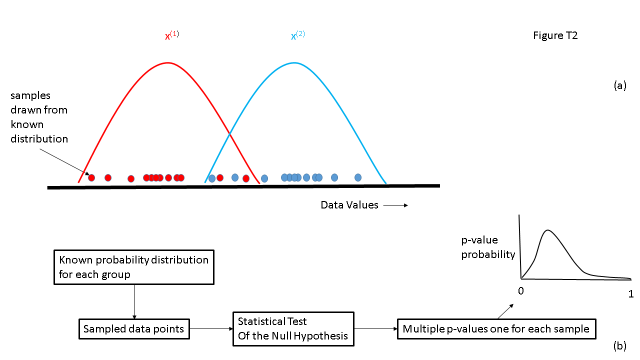


**Figure T3**


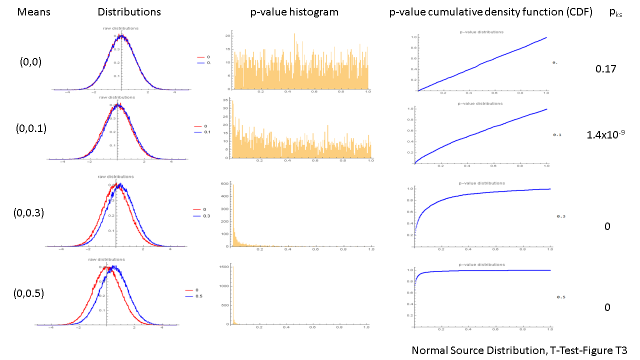


**Figure T4**


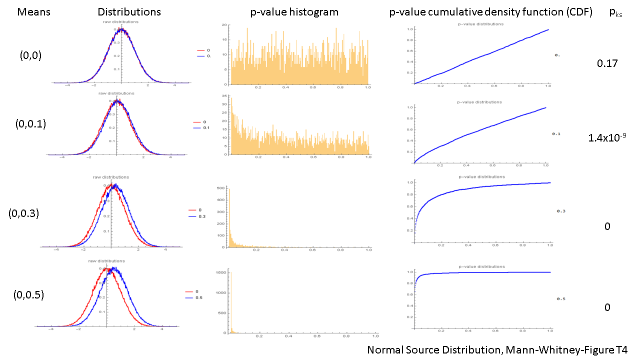


**Figure T5**


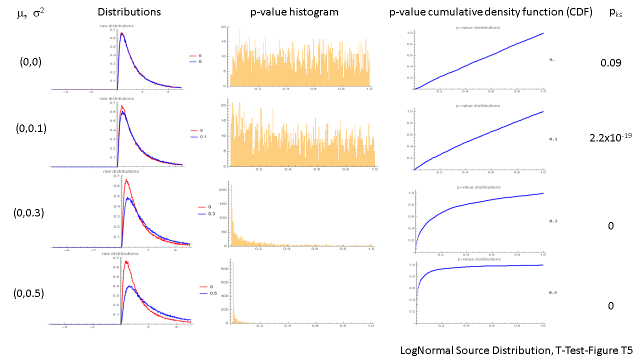


**Figure T6**


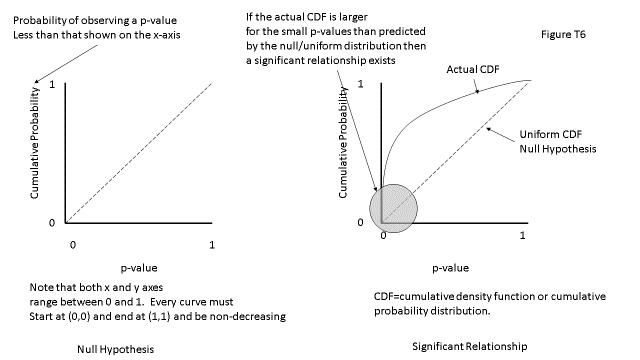


**8.0-Supplementary Spreadsheet Legends**

**Supplementary Spreadsheet S1**. The raw data file used in this study. The labels for each variable are approximations and the actual labels are found by going back to the raw data file using the code number with each entry.

**Supplementary Spreadsheet S2.** Output of the univariate data analysis for all of the data ranked by lowest value of the Spearman p first. The threshold p values to keep the FDR less than 0.001, 0.01, 0.05 and 0.1 are respectively0. 0.002, 0.016, and 0.04. The corresponding Bonferroni correction p values are 0.00002, 0.00009, and .0.0002.

**Supplementary Spreadsheet S3.** Output of the univariate data analysis of the full data set ranked by the degree of correlation between the dependent variable dementia and each independent variable after the effects of the covariates: the fraction of people in each region over 65, the fraction of people that are “white British” and the fraction of people that are female are removed. The threshold p values to keep the FDR less than 0.001, 0.01, 0.05 and 0.1 are respectively 0.0001, 0.002, 0.016, and 0.04. The corresponding Bonferroni correction p values are 0.000002, 0.00002, 0.00009, and .00002. Each cell is colored from red to green based on the value of the Spearman R describing the correlation between the dependent variable and each independent variable. Red colors correspond to positive correlations (ie increase in the independent variable increases the risk of dementia) and green colors indicate negative correlations (ie increase in the independent variable decreases the risk of dementia). More neutral colors indicate variables with minimal effect. For comparison the variable weights determined from the iterative process are shown in column 38. Also shows the approximate variable names derived from the multiple headers in the family food database for each variable number in column 2. The first column shows the headings derived from the raw family food spreadsheet that are more accurate short descriptions of the variable.

**Supplementary Spreadsheet S4**—Correlations between the independent variables found significant in the univariate analysis.

**9.0-Computer Programs**

These programs are attached for review purposes only. No use of this code is authorized without specific advanced written approval from Mark Stecker

1)Mainanalysis.nb—The main data analysis program. The main program allows choice of the root directory to read and write files. The input files must have the same structure as the file attached as supplemental spreadsheet1. The main program allows both simulations and creation of simulated data as well as reading raw data and adding noise. There is the opportunity to change variable selection criteria and criteria for the forward stepwise analyses. Due to the number of computations, the running time can be long.

2)Explorer2—finds basic summary information and graphical and correlation between specified variables.

3)Findweights—This program finds optimal weightings for the food variables and creates a global food index from those weights

4)redoer—analyzes the raw family food data and places into a single spreadsheet for analysis

5)trecoder—collects data from spreadsheets organized by county or ccg and places them in the proper format organized by region of England

**10.0-References**

1. Blumenthal JA, Smith PJ, Mabe S, Hinderliter A, Lin PH, Liao L, et al. Lifestyle and neurocognition in older adults with cognitive impairments: A randomized trial. *Neurology* (2019) **92**:e212-23 doi: 10.1212/WNL.0000000000006784 [doi].

2. Hu EA, Wu A, Dearborn JL, Gottesman RF, Sharrett AR, Steffen LM, et al. Adherence to Dietary Patterns and Risk of Incident Dementia: Findings from the Atherosclerosis Risk in Communities Study. *J Alzheimers Dis* (2020) **78**:827-35 doi: 10.3233/JAD-200392 [doi].

3. Morris MC, Tangney CC, Wang Y, Sacks FM, Bennett DA, Aggarwal NT. MIND diet associated with reduced incidence of Alzheimer's disease. *Alzheimers Dement* (2015) **11**:1007-14 doi: 10.1016/j.jalz.2014.11.009 [doi].

4. de Crom TOE, Mooldijk SS, Ikram MK, Ikram MA, Voortman T. MIND diet and the risk of dementia: a population-based study. *Alzheimers Res Ther* (2022) **14**:8,022-00957-1 doi: 10.1186/s13195-022-00957-1 [doi].

5. Munoz-Garcia MI, Toledo E, Razquin C, Dominguez LJ, Maragarone D, Martinez-Gonzalez J, et al. "A priori" Dietary Patterns and Cognitive Function in the SUN Project. *Neuroepidemiology* (2020) **54**:45-57 doi: 10.1159/000502608 [doi].

6. van den Brink AC, Brouwer-Brolsma EM, Berendsen AAM, van de Rest O. The Mediterranean, Dietary Approaches to Stop Hypertension (DASH), and Mediterranean-DASH Intervention for Neurodegenerative Delay (MIND) Diets Are Associated with Less Cognitive Decline and a Lower Risk of Alzheimer's Disease-A Review. *Adv Nutr* (2019) doi: nmz054 [pii].

7. Hayden KM, Beavers DP, Steck SE, Hebert JR, Tabung FK, Shivappa N, et al. The association between an inflammatory diet and global cognitive function and incident dementia in older women: The Women's Health Initiative Memory Study. *Alzheimers Dement* (2017) **13**:1187-96 doi: S1552-5260(17)30185-1 [pii].

8. Charisis S, Ntanasi E, Yannakoulia M, Anastasiou CA, Kosmidis MH, Dardiotis E, et al. Diet Inflammatory Index and Dementia Incidence: A Population-Based Study. *Neurology* (2021) **97**:e2381-91 doi: 10.1212/WNL.0000000000012973 [doi].

9. Takeuchi H, Kawashima R. Diet and Dementia: A Prospective Study. *Nutrients* (2021) **13**:10.3390/nu13124500 doi: 4500 [pii].

10. Benjamini y, Hochberg y. Controlling the False Discovery Rate: A Practical and Powerful Approach to Multiple Testing. *Journal of the Royal Statistical Society, Series B* (1995) **57**:289-300.

11. Tikhonov AN, Goncharov AV. *Ill-posed problems in the natural sciences.* Moscow: MIR (1987).

12. Grech R, Cassar T, Muscat J, Camilleri KP, Fabri SG, Zervakis M, et al. Review on solving the inverse problem in EEG source analysis. *J Neuroeng Rehabil* (2008) **5**:25,0003-5-25 doi: 10.1186/1743-0003-5-25 [doi].

13. Hoagline DC, . Welsch RE. The hat matrix in regression and ANOVA. *The American Statistician* (1978) **32**:17-22.

14. Bhatnagar P, Wickramasinghe K, Williams J, Rayner M, Townsend N. The epidemiology of cardiovascular disease in the UK 2014. *Heart* (2015) **101**:1182-9 doi: 10.1136/heartjnl-2015-307516 [doi].

15. Shivappa N, Steck SE, Hurley TG, Hussey JR, Hebert JR. Designing and developing a literature-derived, population-based dietary inflammatory index. *Public Health Nutr* (2014) **17**:1689-96 doi: 10.1017/S1368980013002115 [doi].
