# Supplementary material for: Dementia and Diet, Methodological and Statistical Issues: A Pilot Study
Source: Front Aging Neurosci. 2022 Jul 6;14:606424. doi: 10.3389/fnagi.2022.606424 (PMC9298542; doi:10.3389/fnagi.2022.606424)
Supplement: Supplementary file 1 [file Data_Sheet_1.zip › SupplementaryFigures.pptx]

## Slide 1
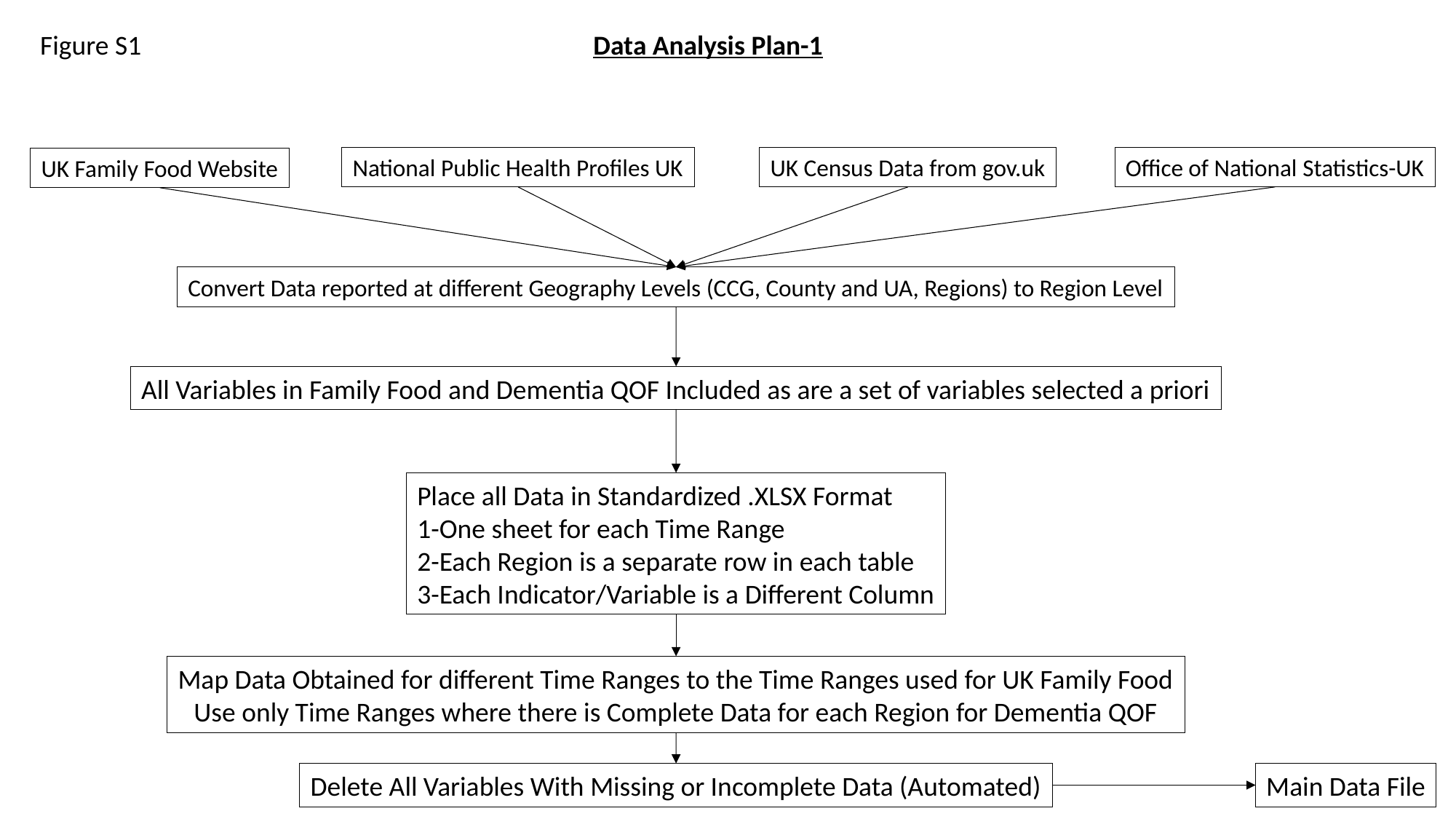

Figure S1
Data Analysis Plan-1
National Public Health Profiles UK
UK Census Data from gov.uk
Office of National Statistics-UK
UK Family Food Website
Convert Data reported at different Geography Levels (CCG, County and UA, Regions) to Region Level
All Variables in Family Food and Dementia QOF Included as are a set of variables selected a priori
Place all Data in Standardized .XLSX Format
1-One sheet for each Time Range
2-Each Region is a separate row in each table
3-Each Indicator/Variable is a Different Column
Map Data Obtained for different Time Ranges to the Time Ranges used for UK Family Food
Use only Time Ranges where there is Complete Data for each Region for Dementia QOF
Delete All Variables With Missing or Incomplete Data (Automated)
Main Data File

## Slide 2
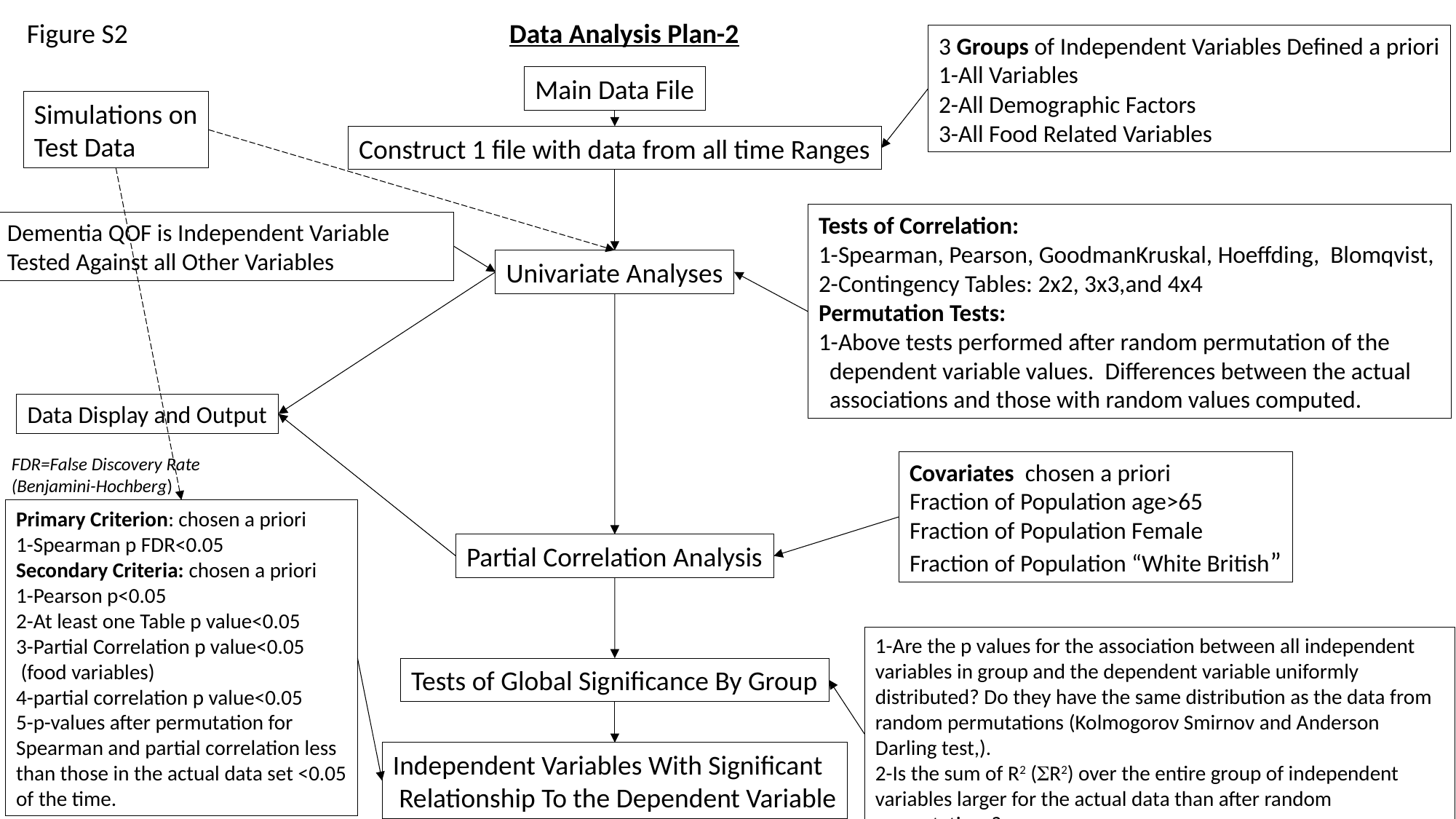

Figure S2
Data Analysis Plan-2
3 Groups of Independent Variables Defined a priori
1-All Variables
2-All Demographic Factors
3-All Food Related Variables
Main Data File
Simulations on
Test Data
Construct 1 file with data from all time Ranges
Tests of Correlation:
1-Spearman, Pearson, GoodmanKruskal, Hoeffding, Blomqvist,
2-Contingency Tables: 2x2, 3x3,and 4x4
Permutation Tests:
1-Above tests performed after random permutation of the
 dependent variable values. Differences between the actual
 associations and those with random values computed.
Dementia QOF is Independent Variable
Tested Against all Other Variables
Univariate Analyses
Data Display and Output
FDR=False Discovery Rate
(Benjamini-Hochberg)
Covariates chosen a priori
Fraction of Population age>65
Fraction of Population Female
Fraction of Population “White British”
Primary Criterion: chosen a priori
1-Spearman p FDR<0.05
Secondary Criteria: chosen a priori
1-Pearson p<0.05
2-At least one Table p value<0.05
3-Partial Correlation p value<0.05
 (food variables)
4-partial correlation p value<0.05
5-p-values after permutation for
Spearman and partial correlation less
than those in the actual data set <0.05
of the time.
Partial Correlation Analysis
1-Are the p values for the association between all independent variables in group and the dependent variable uniformly distributed? Do they have the same distribution as the data from random permutations (Kolmogorov Smirnov and Anderson Darling test,).
2-Is the sum of R2 (SR2) over the entire group of independent variables larger for the actual data than after random permutations?
Tests of Global Significance By Group
Independent Variables With Significant
 Relationship To the Dependent Variable

## Slide 3
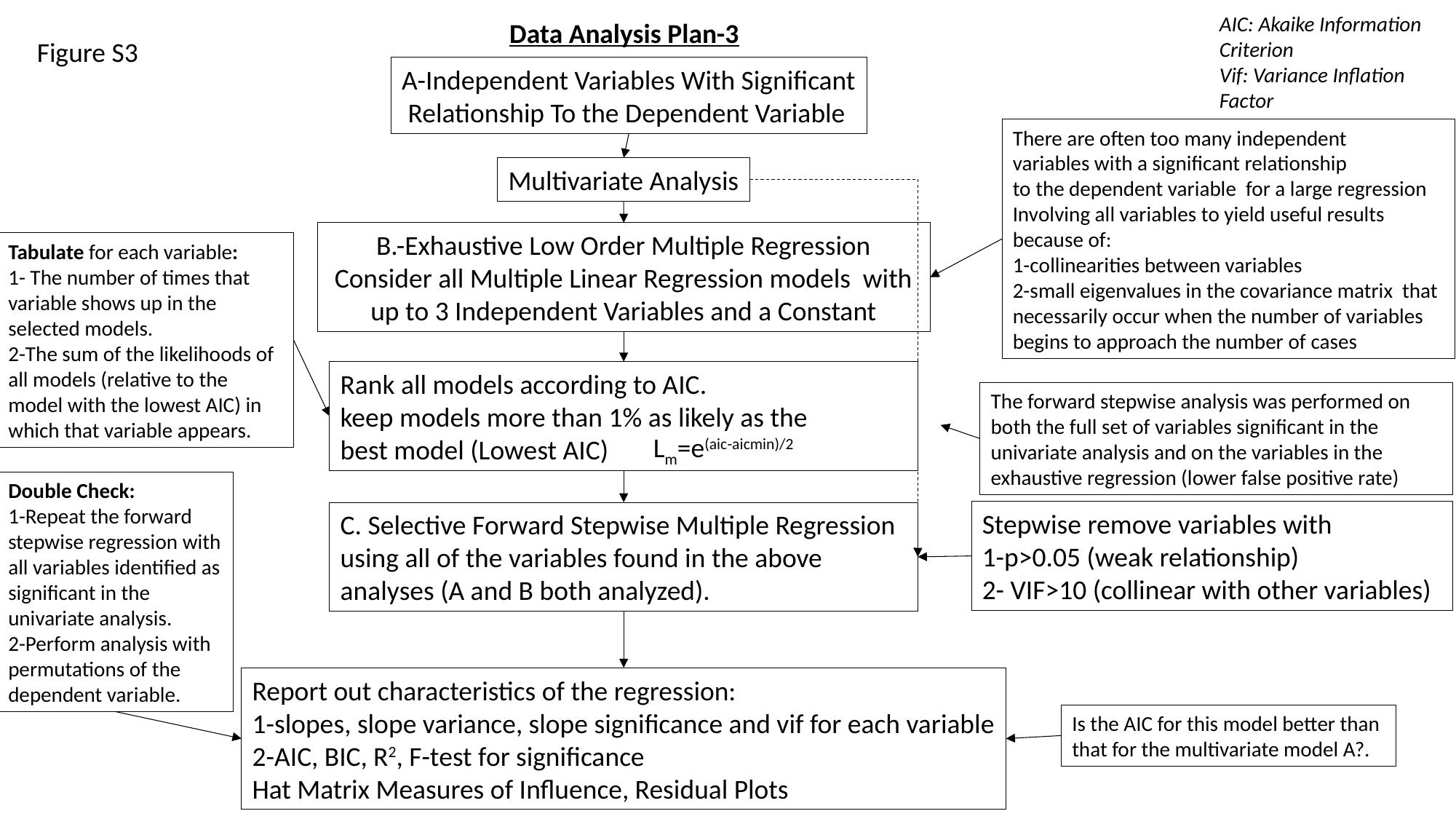

AIC: Akaike Information
Criterion
Vif: Variance Inflation
Factor
Data Analysis Plan-3
Figure S3
A-Independent Variables With Significant
 Relationship To the Dependent Variable
There are often too many independent
variables with a significant relationship
to the dependent variable for a large regression
Involving all variables to yield useful results because of:
1-collinearities between variables
2-small eigenvalues in the covariance matrix that necessarily occur when the number of variables begins to approach the number of cases
Multivariate Analysis
B.-Exhaustive Low Order Multiple Regression
Consider all Multiple Linear Regression models with up to 3 Independent Variables and a Constant
Tabulate for each variable:
1- The number of times that variable shows up in the selected models.
2-The sum of the likelihoods of all models (relative to the model with the lowest AIC) in which that variable appears.
Rank all models according to AIC.
keep models more than 1% as likely as the
best model (Lowest AIC)
The forward stepwise analysis was performed on both the full set of variables significant in the univariate analysis and on the variables in the exhaustive regression (lower false positive rate)
Lm=e(aic-aicmin)/2
Double Check:
1-Repeat the forward stepwise regression with all variables identified as significant in the
univariate analysis.
2-Perform analysis with
permutations of the
dependent variable.
Stepwise remove variables with
1-p>0.05 (weak relationship)
2- VIF>10 (collinear with other variables)
C. Selective Forward Stepwise Multiple Regression using all of the variables found in the above analyses (A and B both analyzed).
Report out characteristics of the regression:
1-slopes, slope variance, slope significance and vif for each variable
2-AIC, BIC, R2, F-test for significance
Hat Matrix Measures of Influence, Residual Plots
Is the AIC for this model better than that for the multivariate model A?.

## Slide 4
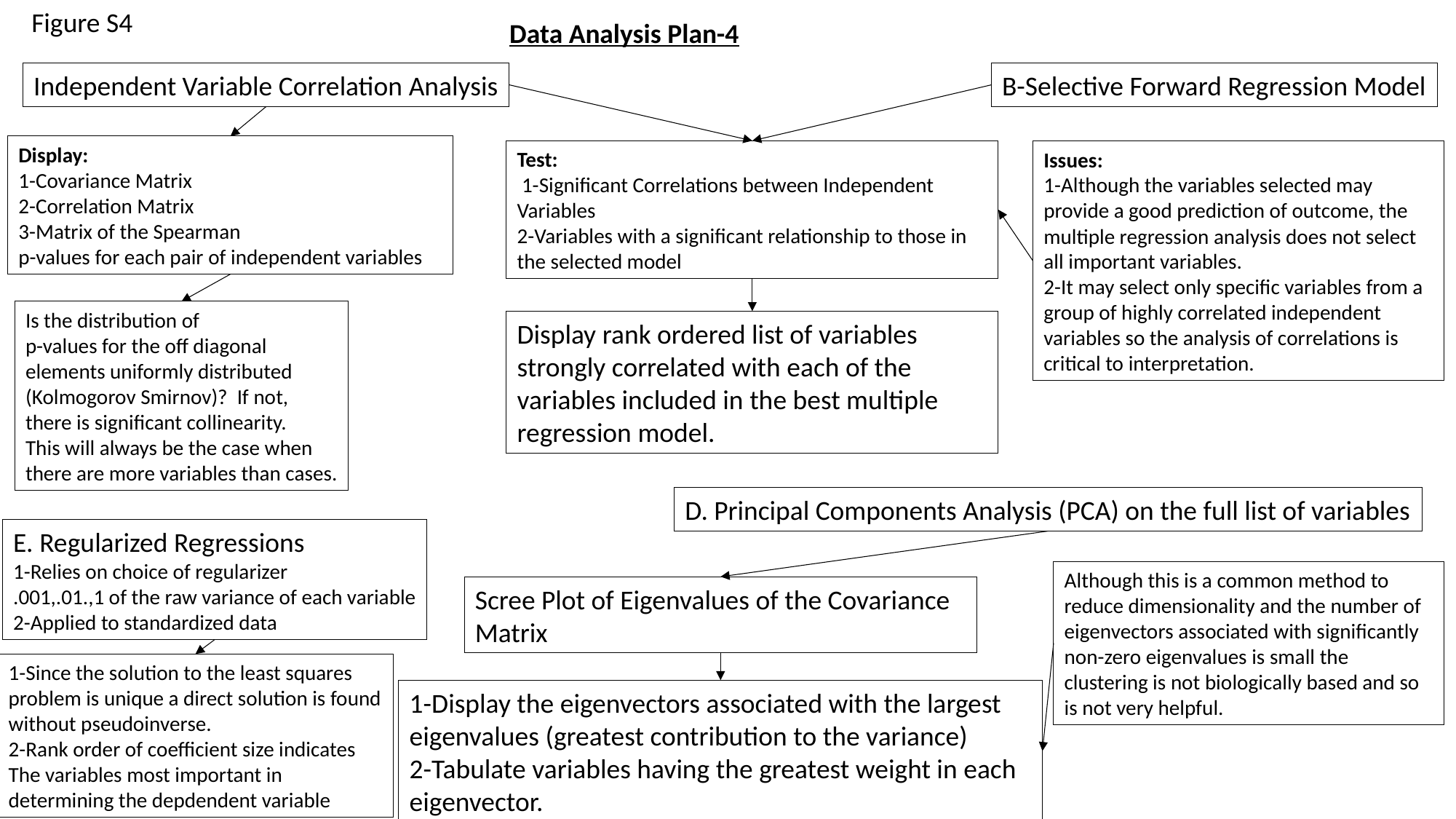

Figure S4
Data Analysis Plan-4
Independent Variable Correlation Analysis
B-Selective Forward Regression Model
Display:
1-Covariance Matrix
2-Correlation Matrix
3-Matrix of the Spearman
p-values for each pair of independent variables
Test:
 1-Significant Correlations between Independent Variables
2-Variables with a significant relationship to those in the selected model
Issues:
1-Although the variables selected may provide a good prediction of outcome, the multiple regression analysis does not select all important variables.
2-It may select only specific variables from a group of highly correlated independent variables so the analysis of correlations is critical to interpretation.
Is the distribution of
p-values for the off diagonal
elements uniformly distributed
(Kolmogorov Smirnov)? If not,
there is significant collinearity.
This will always be the case when
there are more variables than cases.
Display rank ordered list of variables strongly correlated with each of the variables included in the best multiple regression model.
D. Principal Components Analysis (PCA) on the full list of variables
E. Regularized Regressions
1-Relies on choice of regularizer
.001,.01.,1 of the raw variance of each variable
2-Applied to standardized data
Although this is a common method to reduce dimensionality and the number of
eigenvectors associated with significantly non-zero eigenvalues is small the clustering is not biologically based and so is not very helpful.
Scree Plot of Eigenvalues of the Covariance Matrix
1-Since the solution to the least squares problem is unique a direct solution is found without pseudoinverse.
2-Rank order of coefficient size indicates
The variables most important in determining the depdendent variable
1-Display the eigenvectors associated with the largest eigenvalues (greatest contribution to the variance)
2-Tabulate variables having the greatest weight in each eigenvector.

## Slide 5
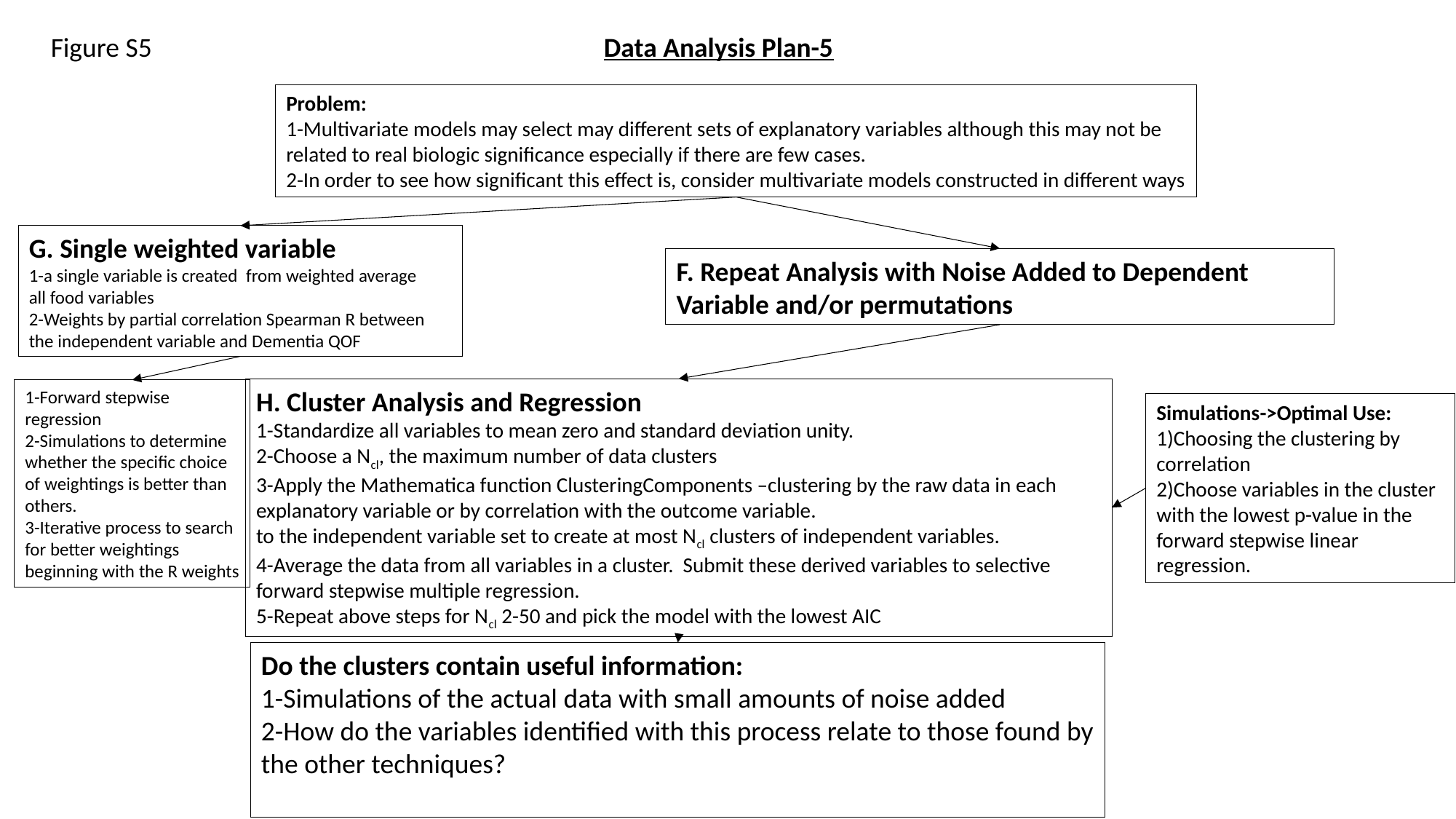

Figure S5
Data Analysis Plan-5
Problem:
1-Multivariate models may select may different sets of explanatory variables although this may not be
related to real biologic significance especially if there are few cases.
2-In order to see how significant this effect is, consider multivariate models constructed in different ways
G. Single weighted variable
1-a single variable is created from weighted average
all food variables
2-Weights by partial correlation Spearman R between the independent variable and Dementia QOF
F. Repeat Analysis with Noise Added to Dependent Variable and/or permutations
H. Cluster Analysis and Regression
1-Standardize all variables to mean zero and standard deviation unity.
2-Choose a Ncl, the maximum number of data clusters
3-Apply the Mathematica function ClusteringComponents –clustering by the raw data in each explanatory variable or by correlation with the outcome variable.
to the independent variable set to create at most Ncl clusters of independent variables.
4-Average the data from all variables in a cluster. Submit these derived variables to selective forward stepwise multiple regression.
5-Repeat above steps for Ncl 2-50 and pick the model with the lowest AIC
1-Forward stepwise
regression
2-Simulations to determine
whether the specific choice
of weightings is better than
others.
3-Iterative process to search
for better weightings
beginning with the R weights
Simulations->Optimal Use:
1)Choosing the clustering by correlation
2)Choose variables in the cluster with the lowest p-value in the forward stepwise linear regression.
Do the clusters contain useful information:
1-Simulations of the actual data with small amounts of noise added
2-How do the variables identified with this process relate to those found by
the other techniques?

## Slide 6
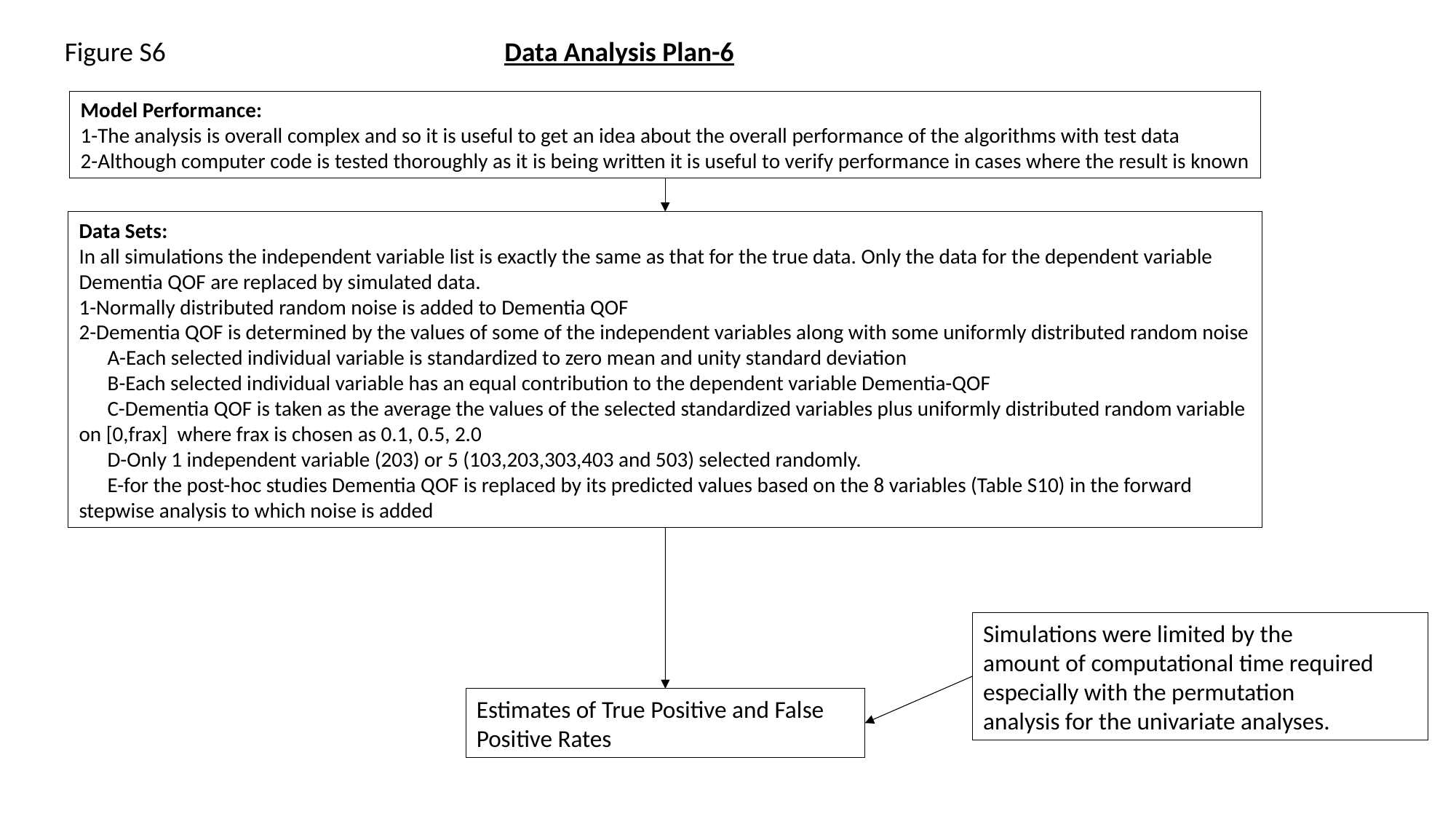

Figure S6
Data Analysis Plan-6
Model Performance:
1-The analysis is overall complex and so it is useful to get an idea about the overall performance of the algorithms with test data
2-Although computer code is tested thoroughly as it is being written it is useful to verify performance in cases where the result is known
Data Sets:
In all simulations the independent variable list is exactly the same as that for the true data. Only the data for the dependent variable Dementia QOF are replaced by simulated data.
1-Normally distributed random noise is added to Dementia QOF
2-Dementia QOF is determined by the values of some of the independent variables along with some uniformly distributed random noise
 A-Each selected individual variable is standardized to zero mean and unity standard deviation
 B-Each selected individual variable has an equal contribution to the dependent variable Dementia-QOF
 C-Dementia QOF is taken as the average the values of the selected standardized variables plus uniformly distributed random variable on [0,frax] where frax is chosen as 0.1, 0.5, 2.0
 D-Only 1 independent variable (203) or 5 (103,203,303,403 and 503) selected randomly.
 E-for the post-hoc studies Dementia QOF is replaced by its predicted values based on the 8 variables (Table S10) in the forward stepwise analysis to which noise is added
Simulations were limited by the
amount of computational time required especially with the permutation
analysis for the univariate analyses.
Estimates of True Positive and False Positive Rates

## Slide 7
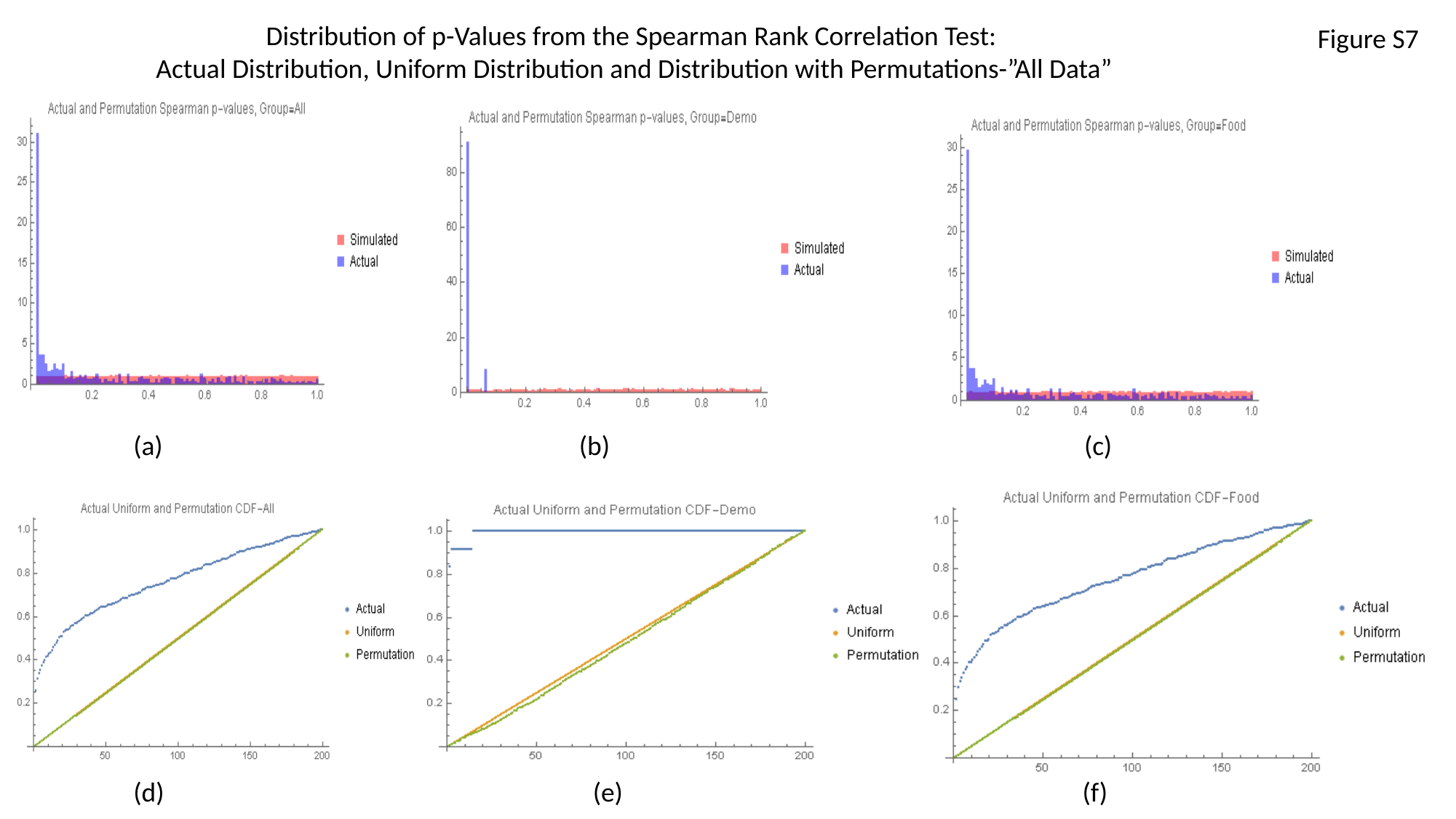

Distribution of p-Values from the Spearman Rank Correlation Test:
Actual Distribution, Uniform Distribution and Distribution with Permutations-”All Data”
Figure S7
(a)
(b)
(c)
(d)
 (e)
(f)

## Slide 8
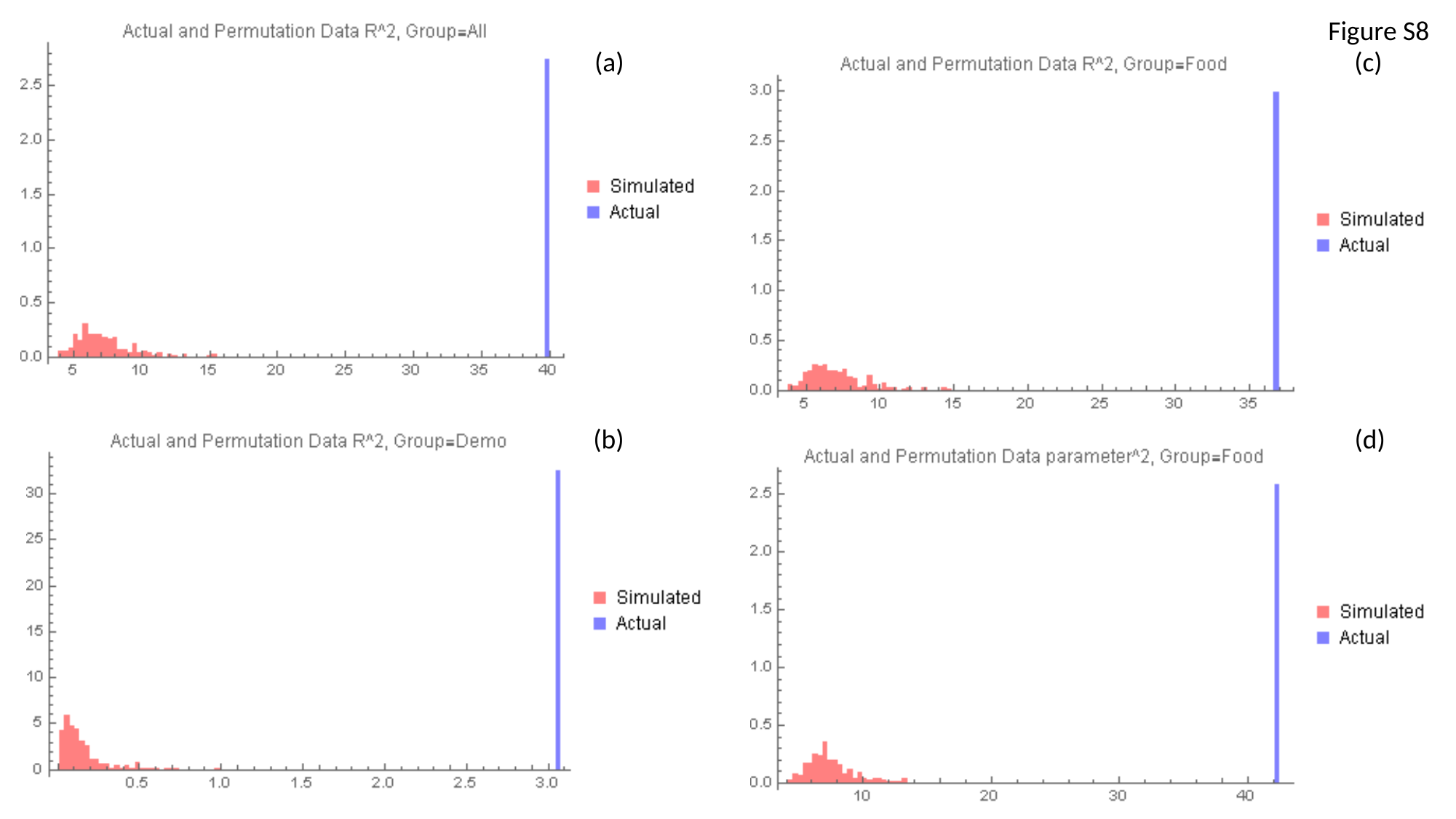

Figure S8
(a)
(c)
(b)
(d)

## Slide 9
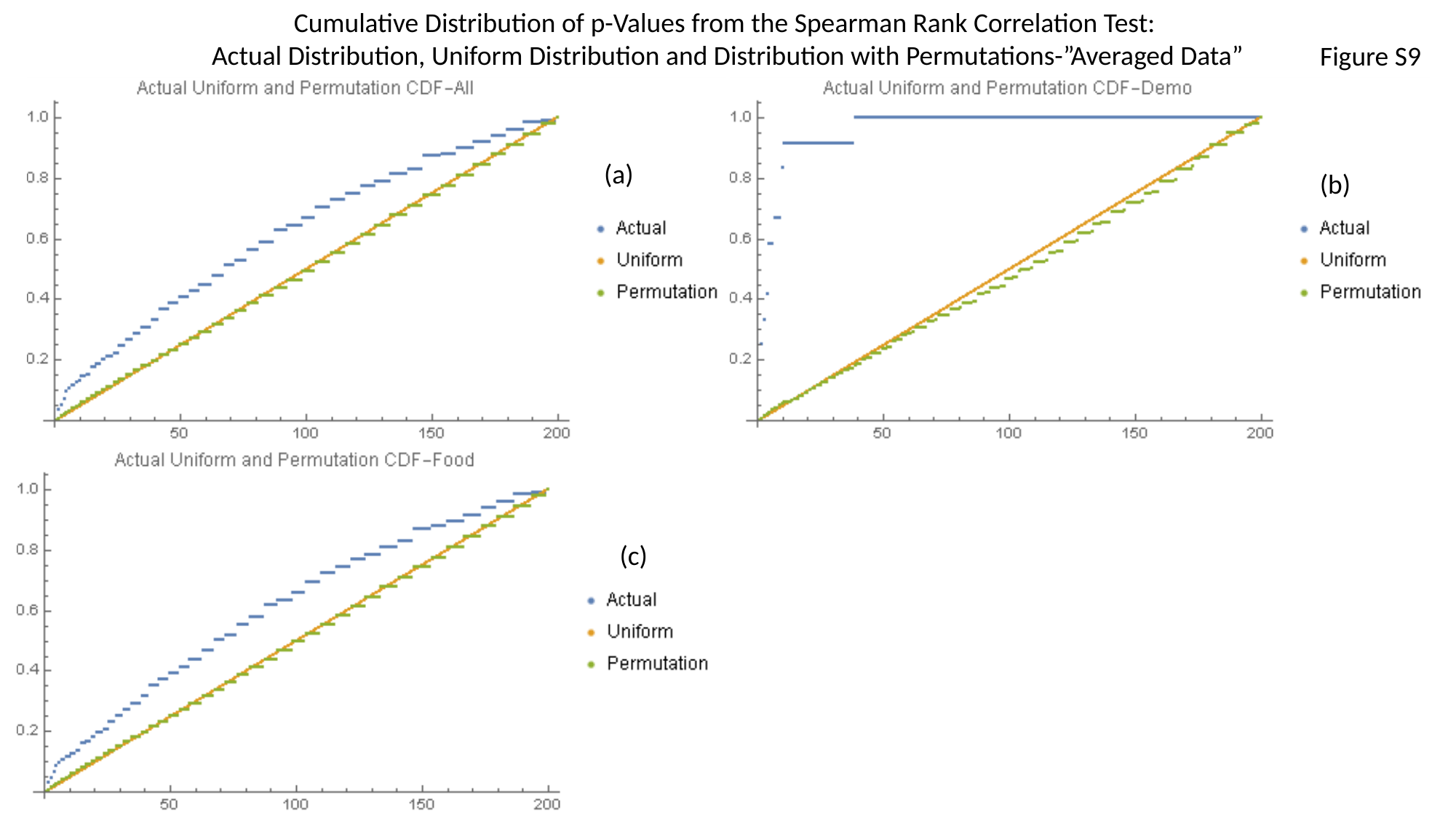

Cumulative Distribution of p-Values from the Spearman Rank Correlation Test:
Actual Distribution, Uniform Distribution and Distribution with Permutations-”Averaged Data”
Figure S9
(a)
(b)
(c)

## Slide 10
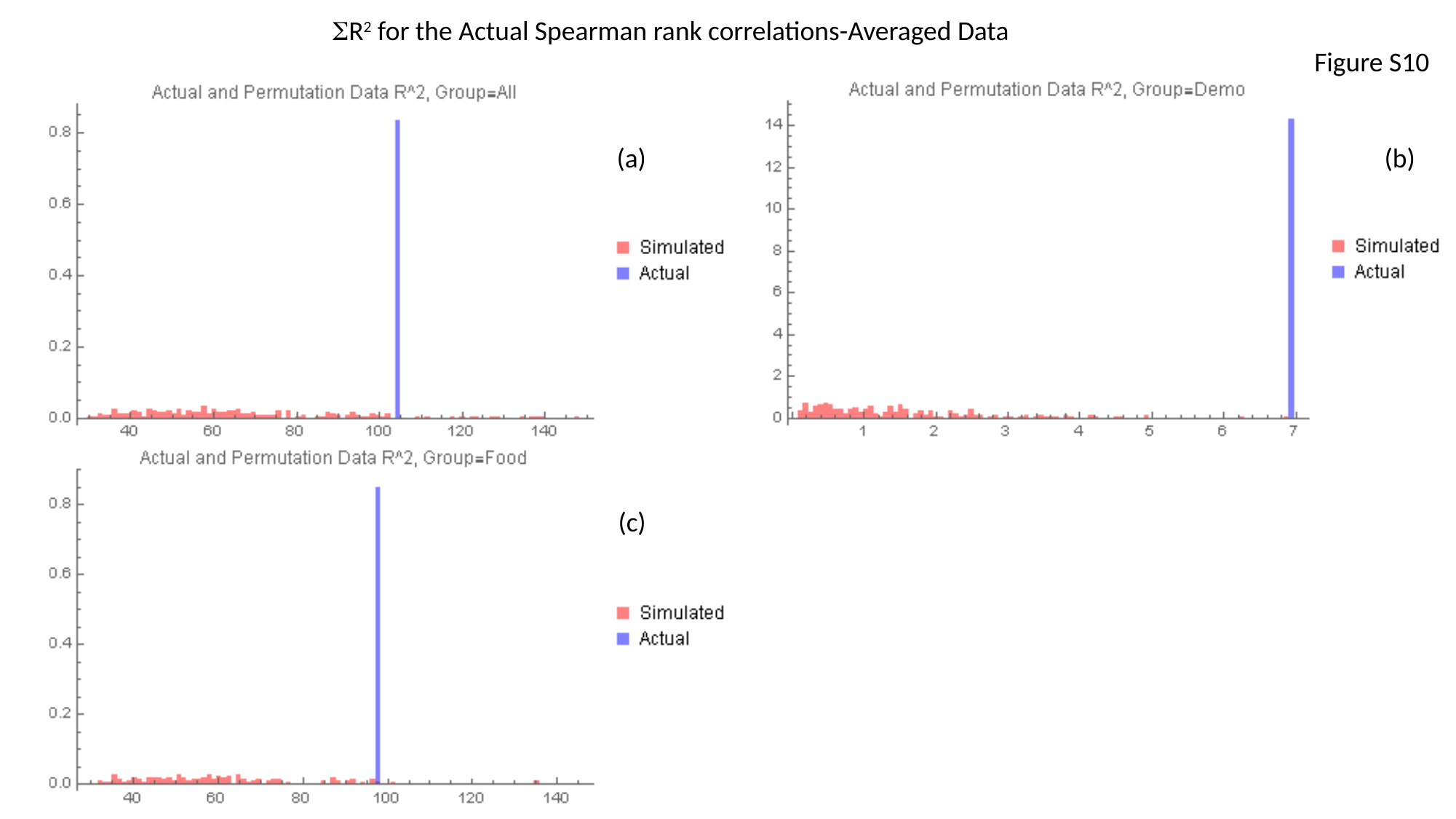

SR2 for the Actual Spearman rank correlations-Averaged Data
Figure S10
(a)
(b)
(c)

## Slide 11
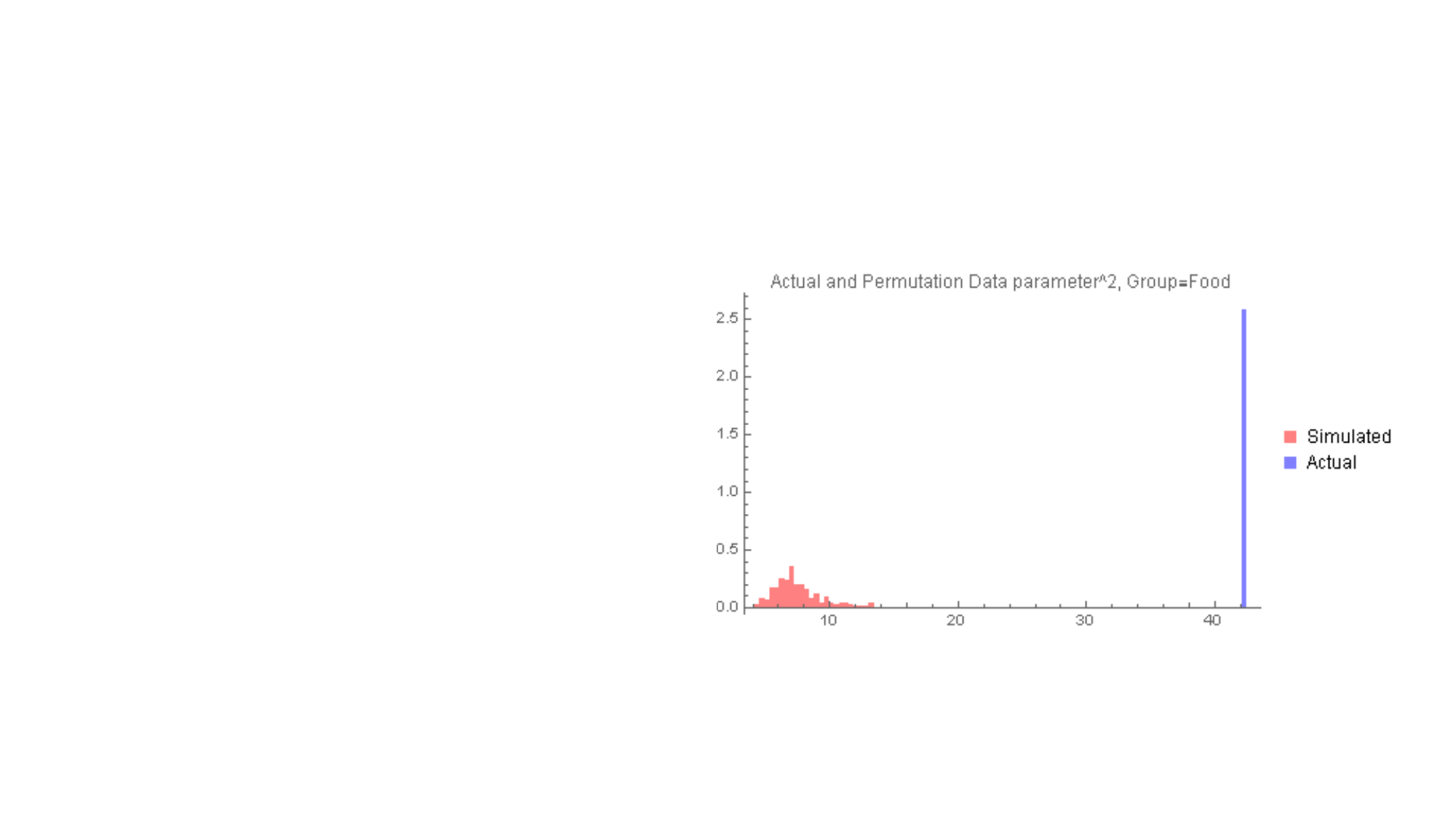

## Slide 12
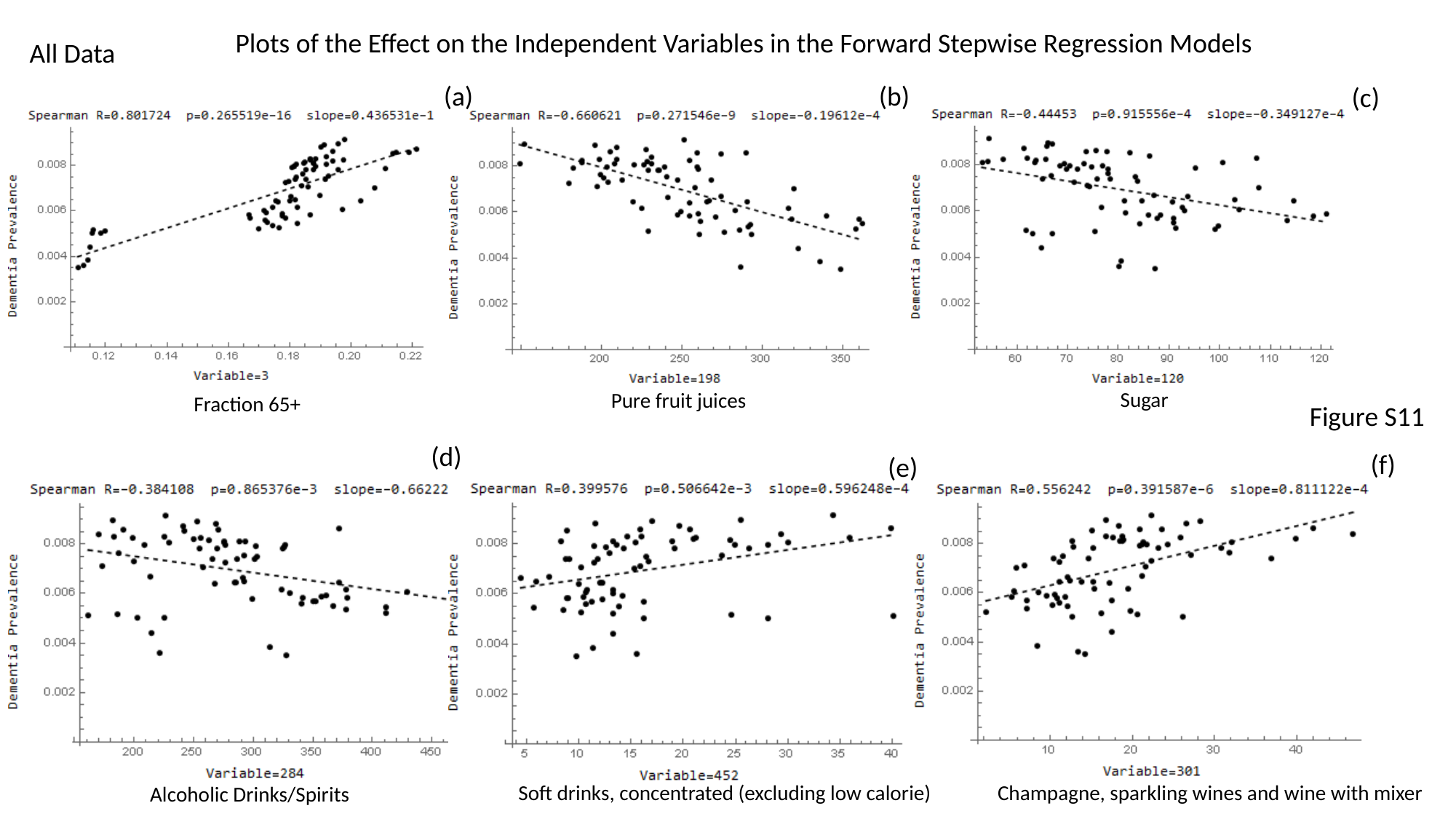

Plots of the Effect on the Independent Variables in the Forward Stepwise Regression Models
All Data
(a)
(b)
(c)
Sugar
Pure fruit juices
Fraction 65+
Figure S11
(d)
(f)
(e)
Champagne, sparkling wines and wine with mixer
Soft drinks, concentrated (excluding low calorie)
Alcoholic Drinks/Spirits

## Slide 13
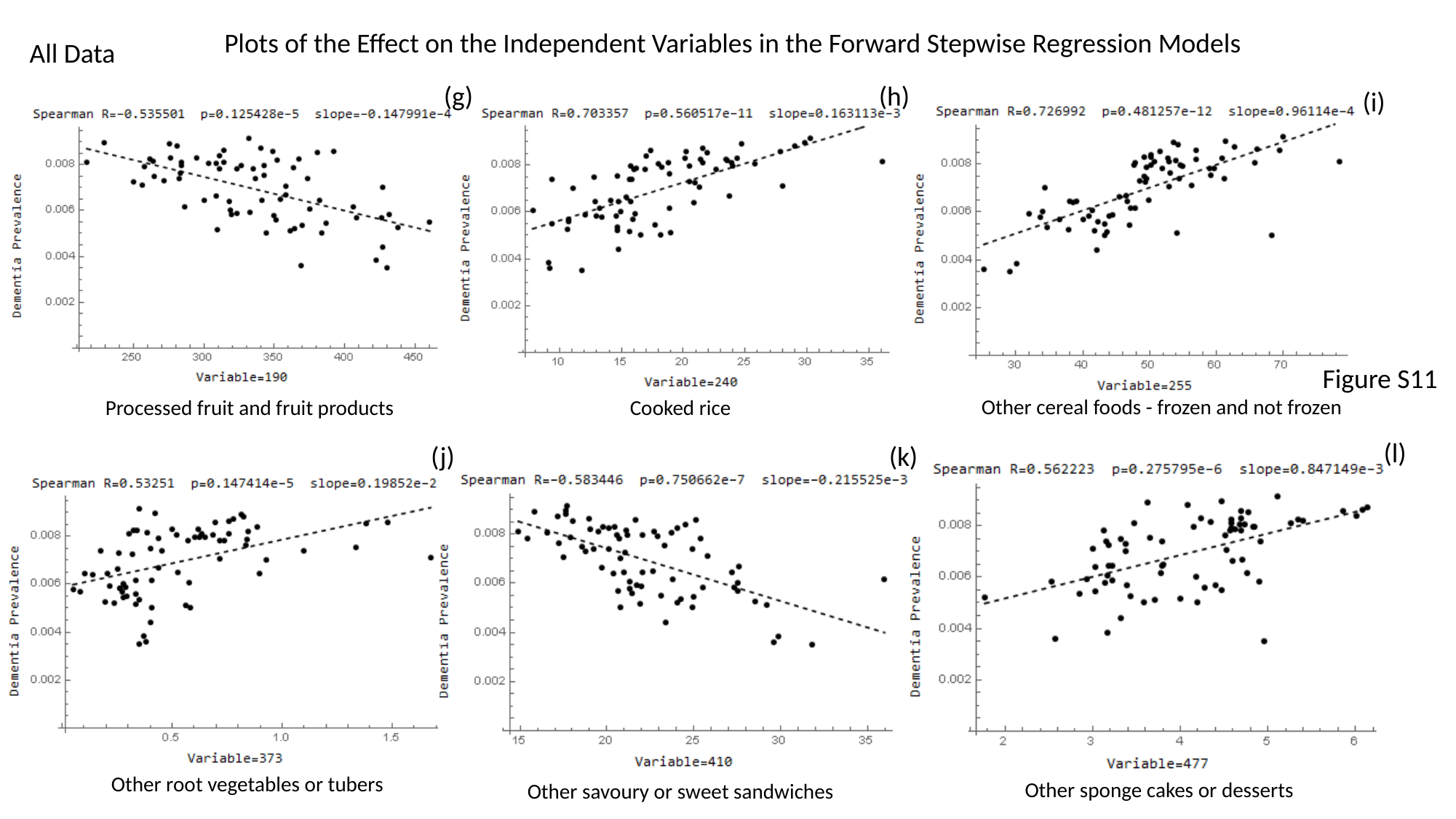

Plots of the Effect on the Independent Variables in the Forward Stepwise Regression Models
All Data
(g)
(h)
(i)
Figure S11
Other cereal foods - frozen and not frozen
Processed fruit and fruit products
Cooked rice
(l)
(k)
(j)
Other root vegetables or tubers
Other sponge cakes or desserts
Other savoury or sweet sandwiches

## Slide 14
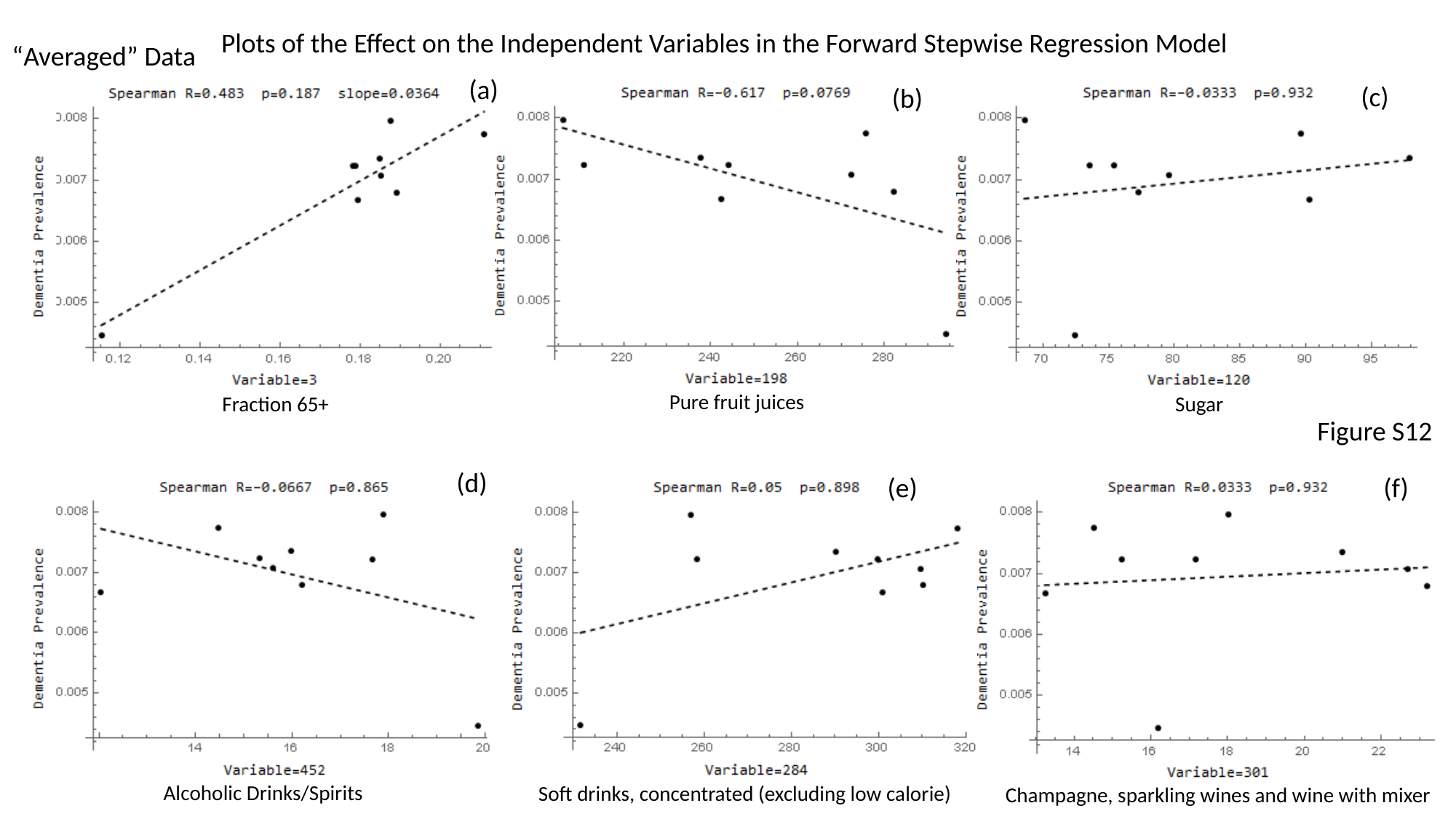

Plots of the Effect on the Independent Variables in the Forward Stepwise Regression Model
“Averaged” Data
(a)
(c)
(b)
Pure fruit juices
Sugar
Fraction 65+
Figure S12
(d)
(e)
(f)
Alcoholic Drinks/Spirits
Soft drinks, concentrated (excluding low calorie)
Champagne, sparkling wines and wine with mixer

## Slide 15
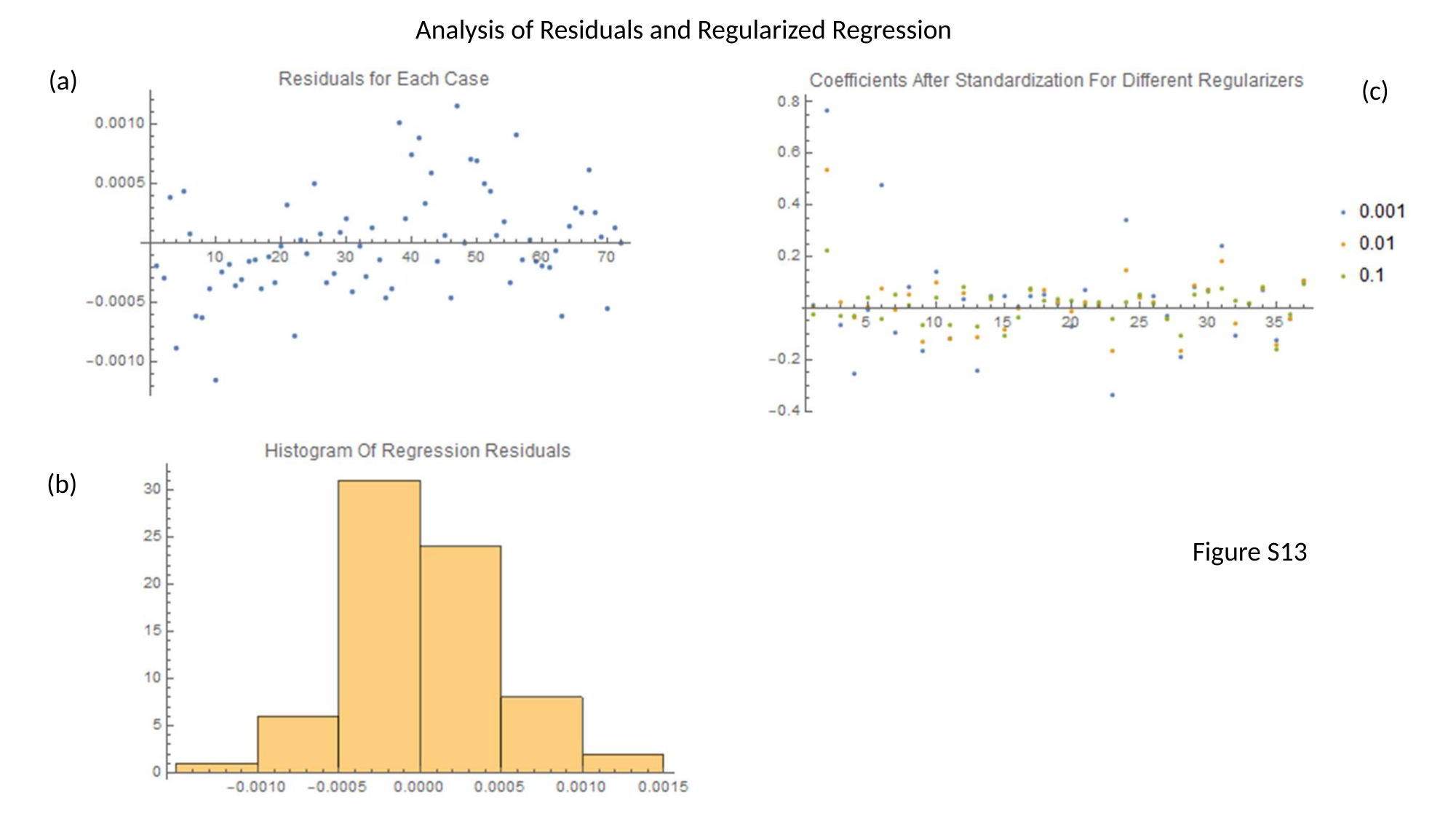

Analysis of Residuals and Regularized Regression
(a)
(c)
(b)
Figure S13

## Slide 16
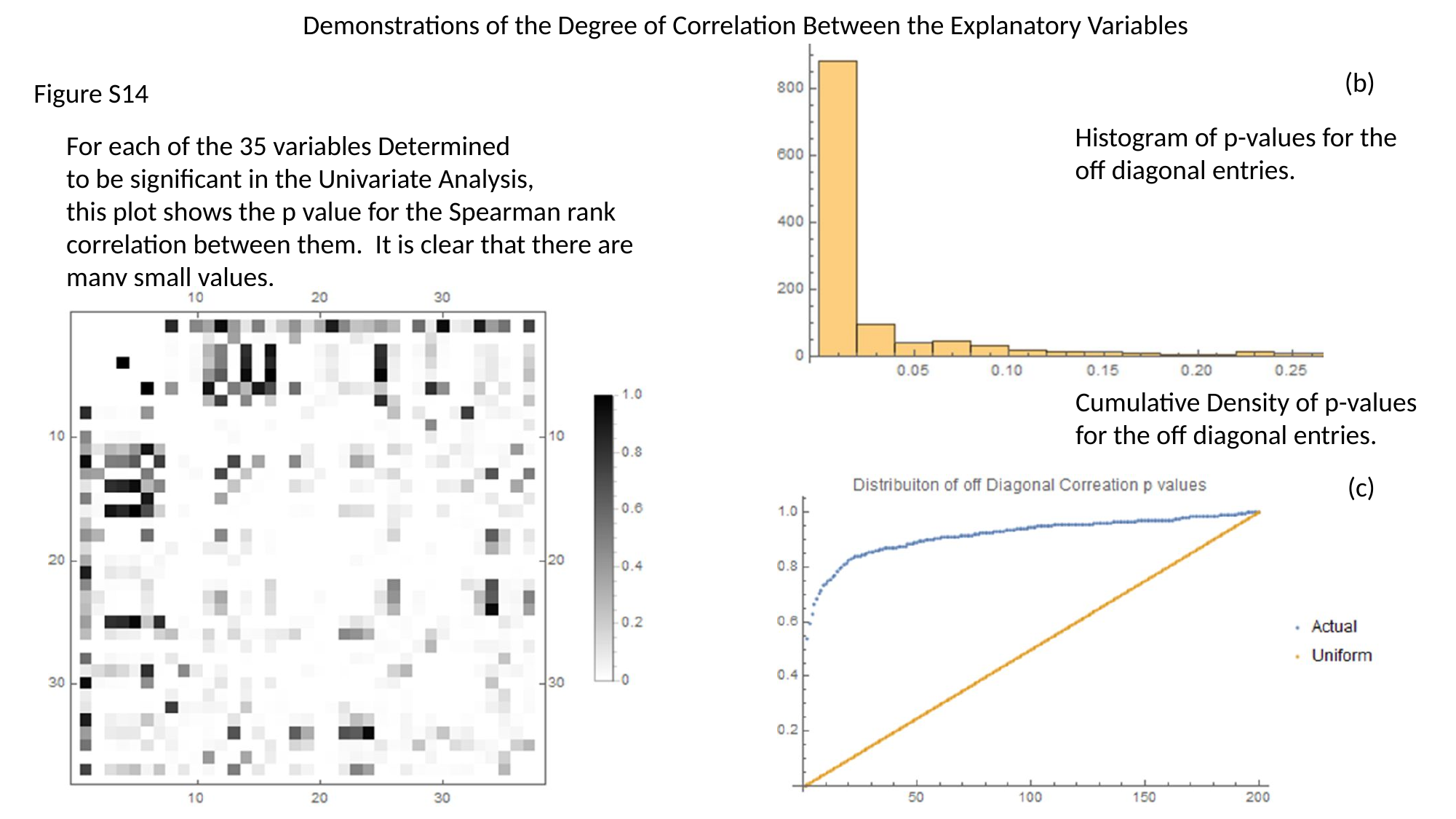

Demonstrations of the Degree of Correlation Between the Explanatory Variables
(b)
Figure S14
Histogram of p-values for the
off diagonal entries.
For each of the 35 variables Determined
to be significant in the Univariate Analysis,
this plot shows the p value for the Spearman rank
correlation between them. It is clear that there are
many small values.
(a)
Cumulative Density of p-values
for the off diagonal entries.
(c)

## Slide 17
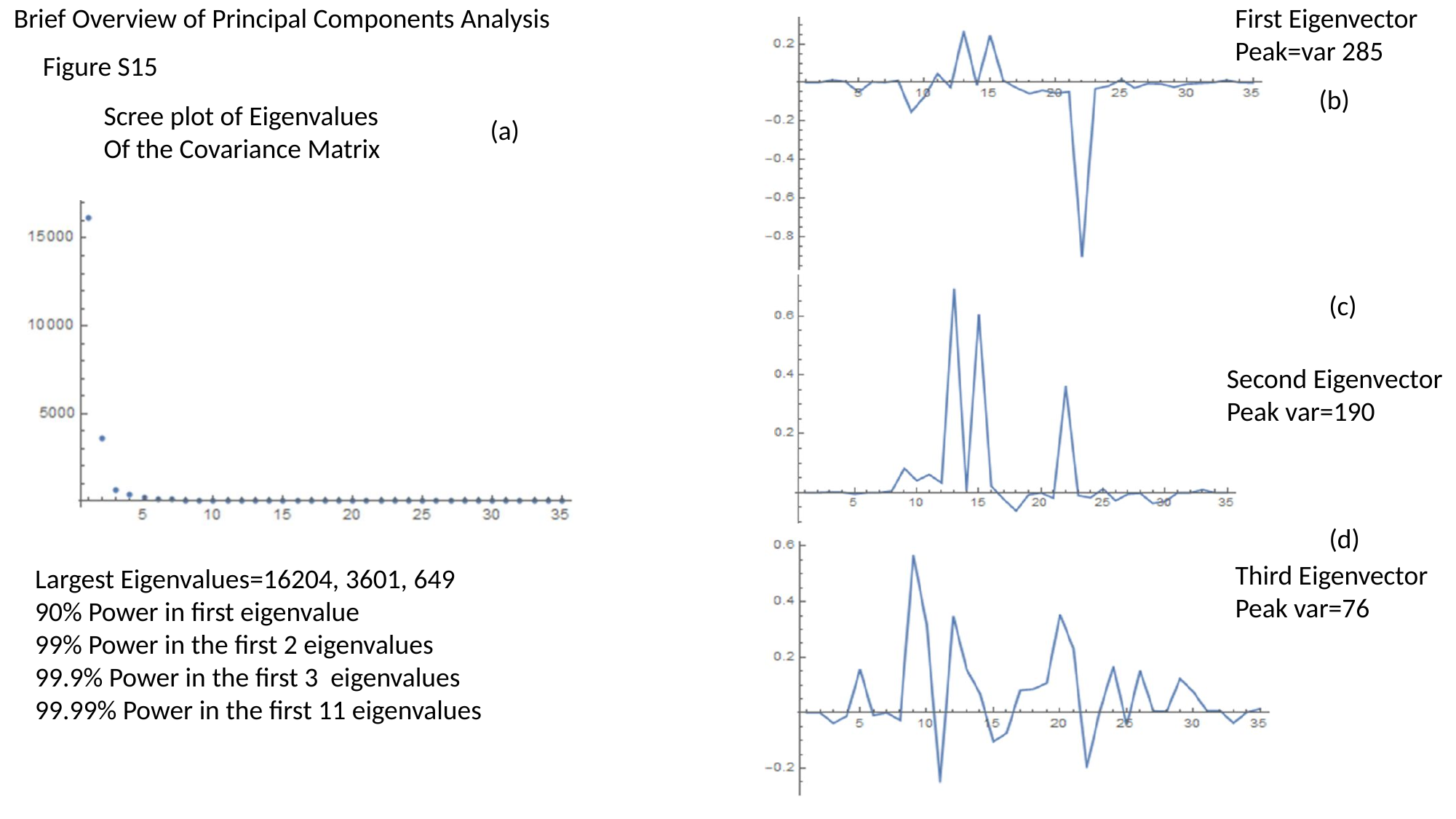

Brief Overview of Principal Components Analysis
First Eigenvector
Peak=var 285
Figure S15
(b)
Scree plot of Eigenvalues
Of the Covariance Matrix
(a)
(c)
Second Eigenvector
Peak var=190
(d)
Third Eigenvector
Peak var=76
Largest Eigenvalues=16204, 3601, 649
90% Power in first eigenvalue
99% Power in the first 2 eigenvalues
99.9% Power in the first 3 eigenvalues
99.99% Power in the first 11 eigenvalues

## Slide 18
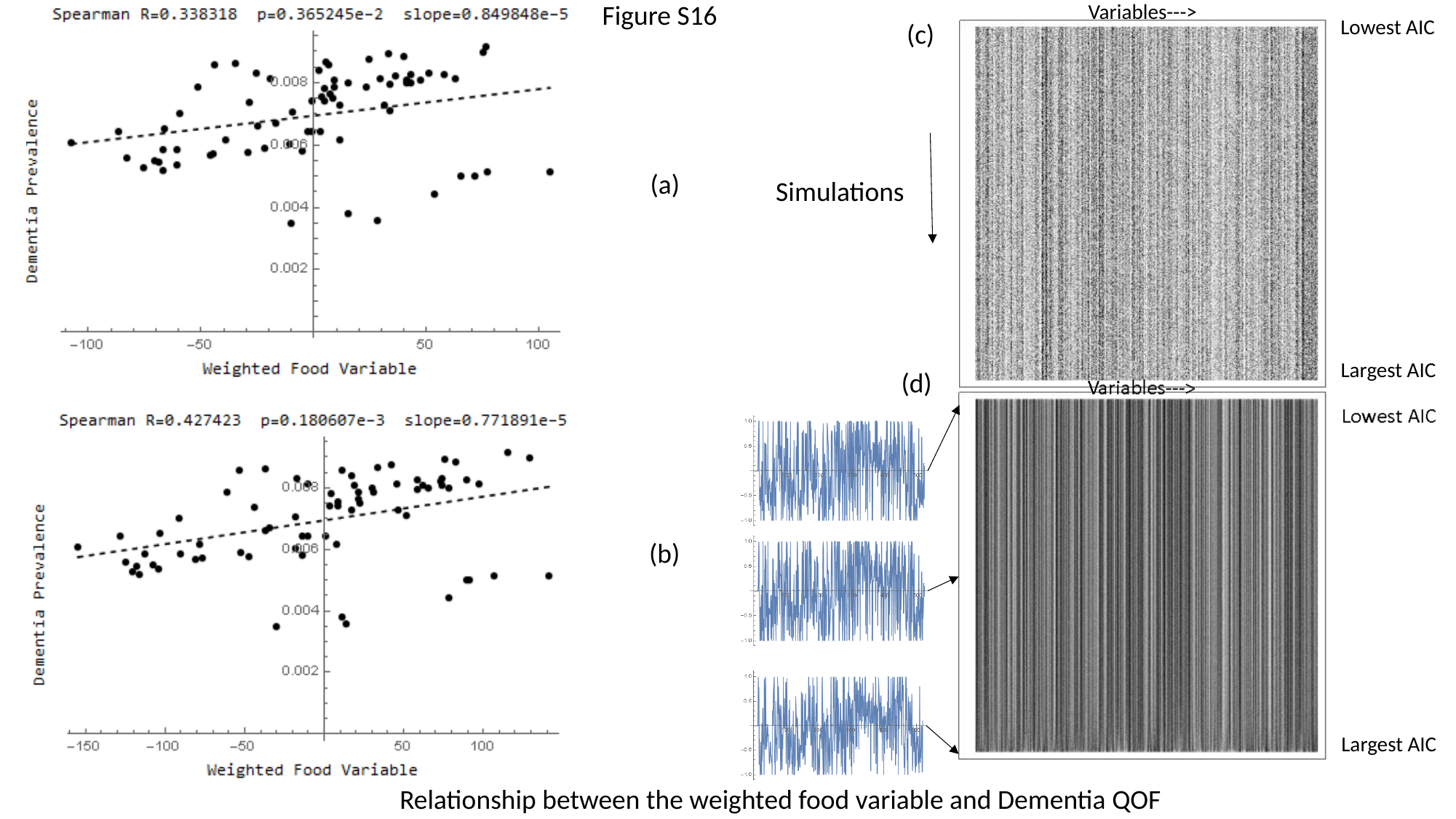

Figure S16
Variables--->
Lowest AIC
(c)
(a)
Simulations
Largest AIC
(d)
(b)
Largest AIC
Relationship between the weighted food variable and Dementia QOF

## Slide 19
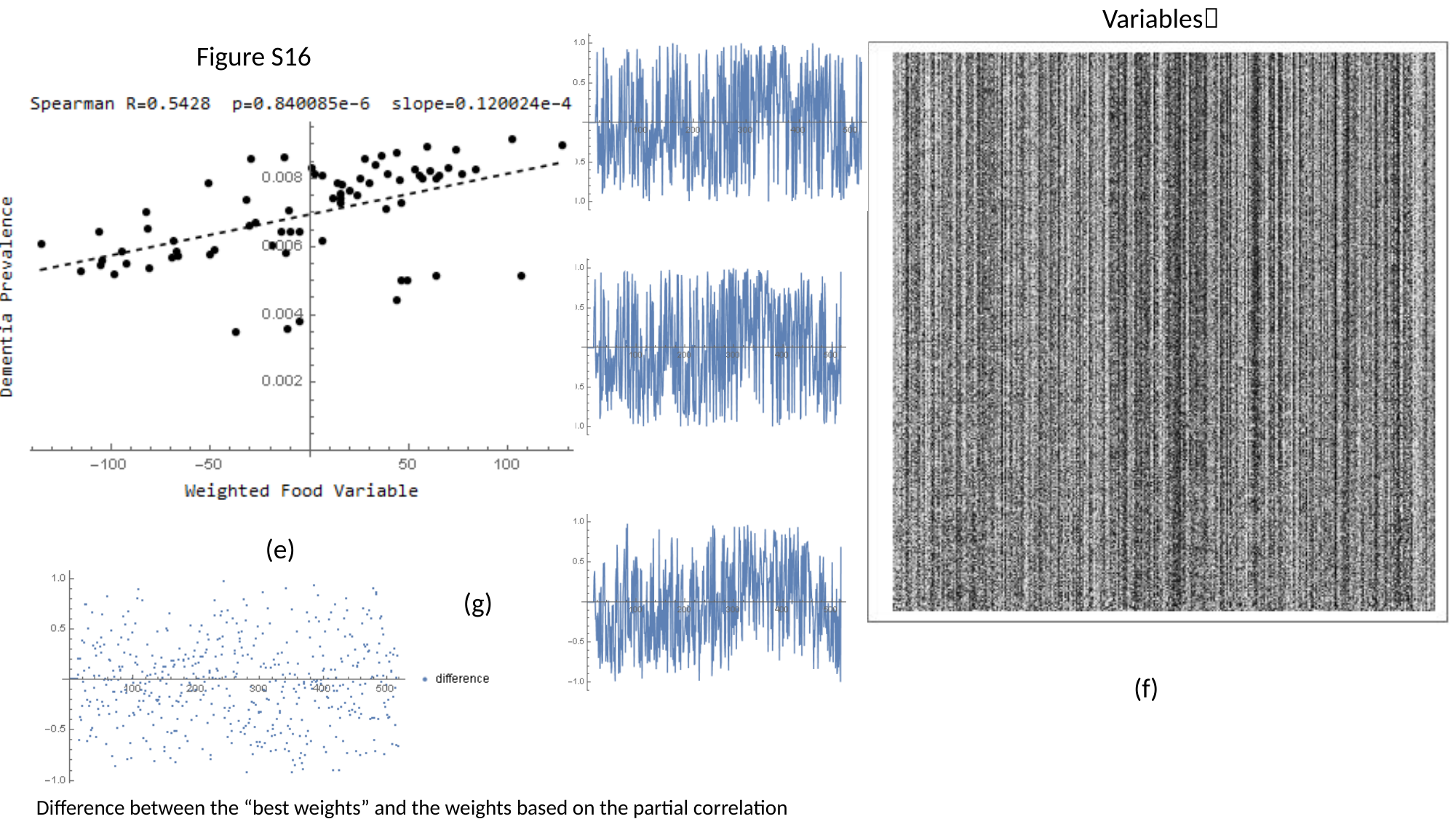

Variables
Figure S16
(e)
(g)
(f)
Difference between the “best weights” and the weights based on the partial correlation

## Slide 20
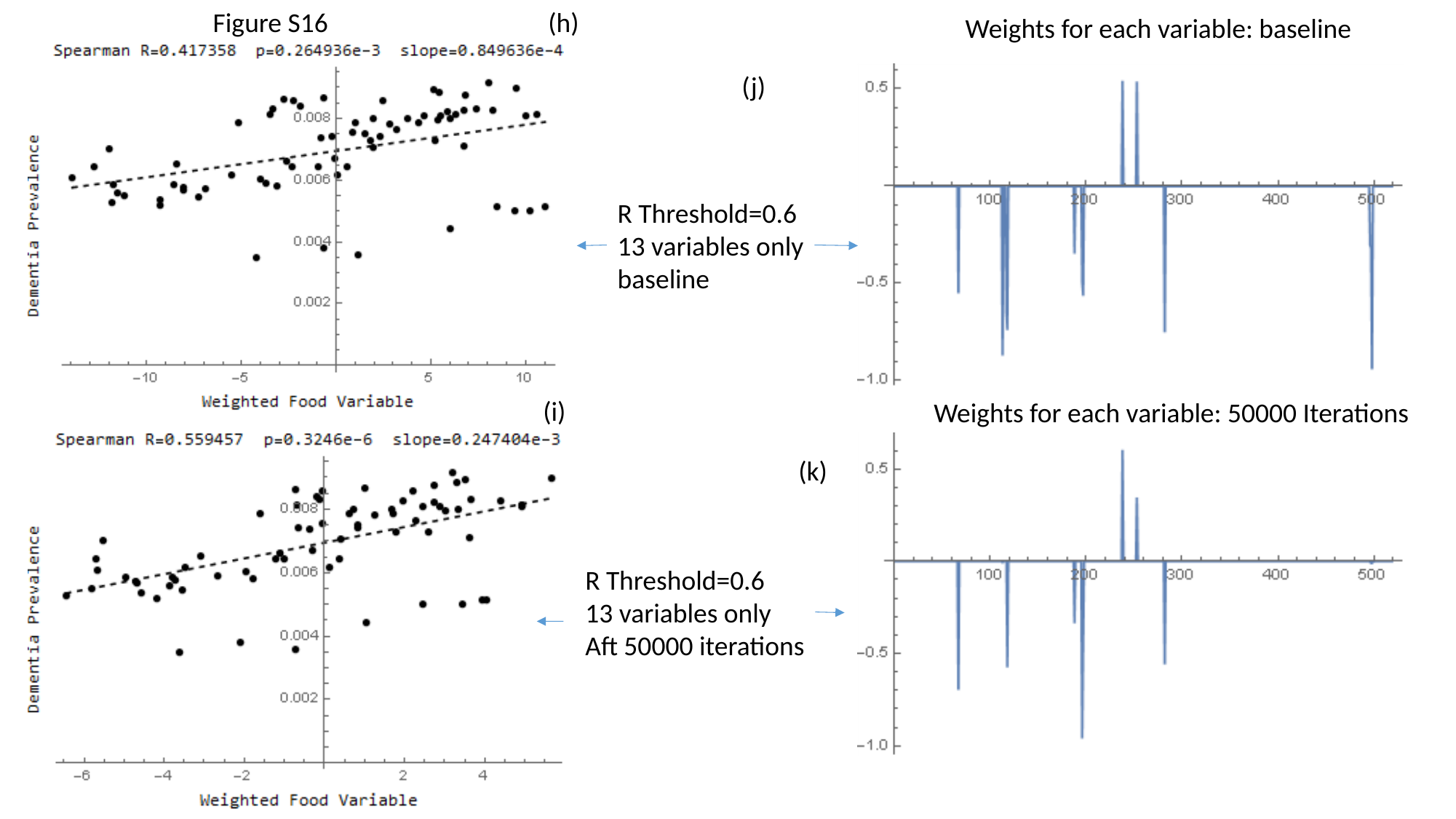

Figure S16
(h)
Weights for each variable: baseline
(j)
R Threshold=0.6
13 variables only
baseline
(i)
Weights for each variable: 50000 Iterations
(k)
R Threshold=0.6
13 variables only
Aft 50000 iterations

## Slide 21
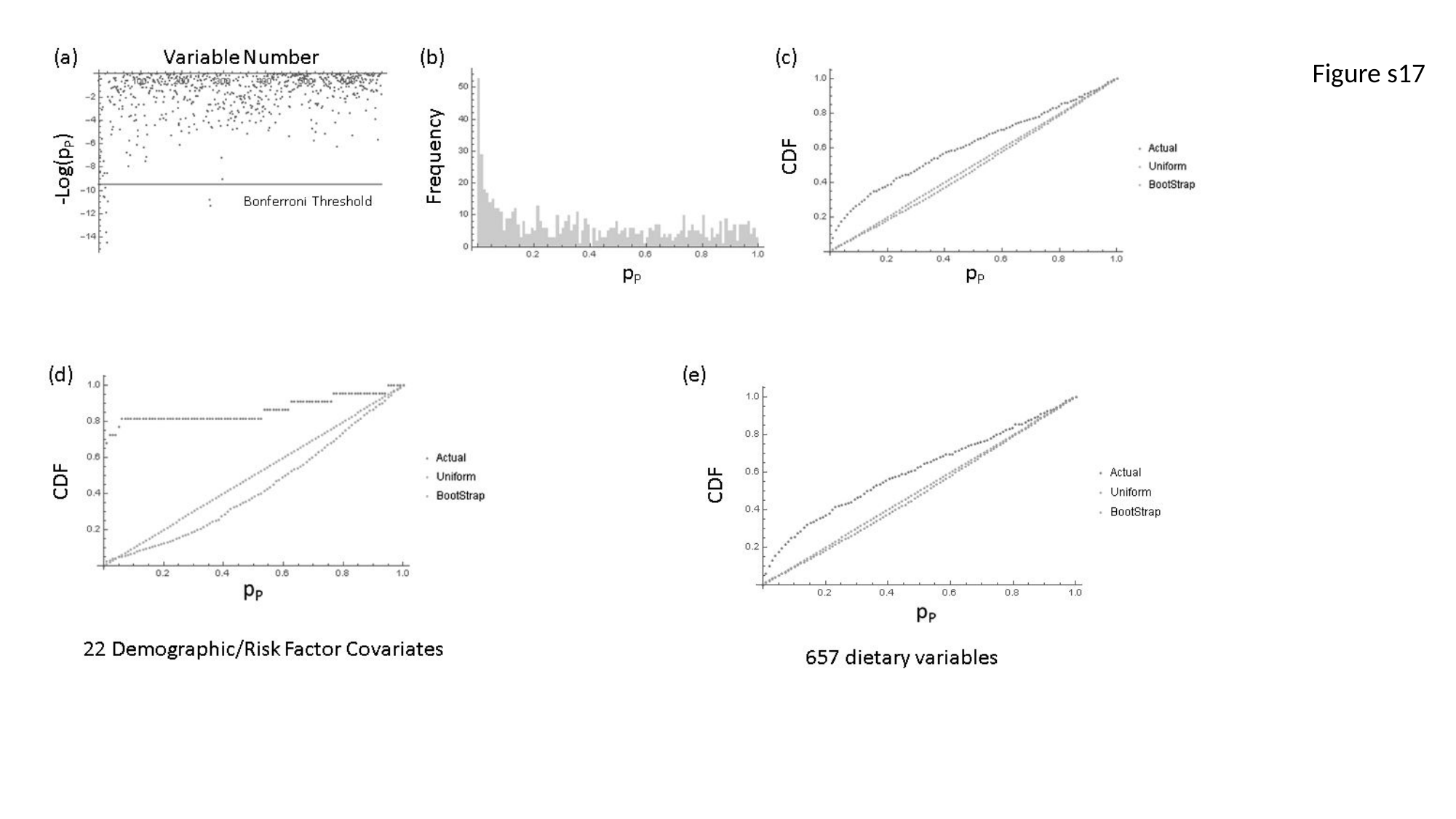

Figure s17

## Slide 22
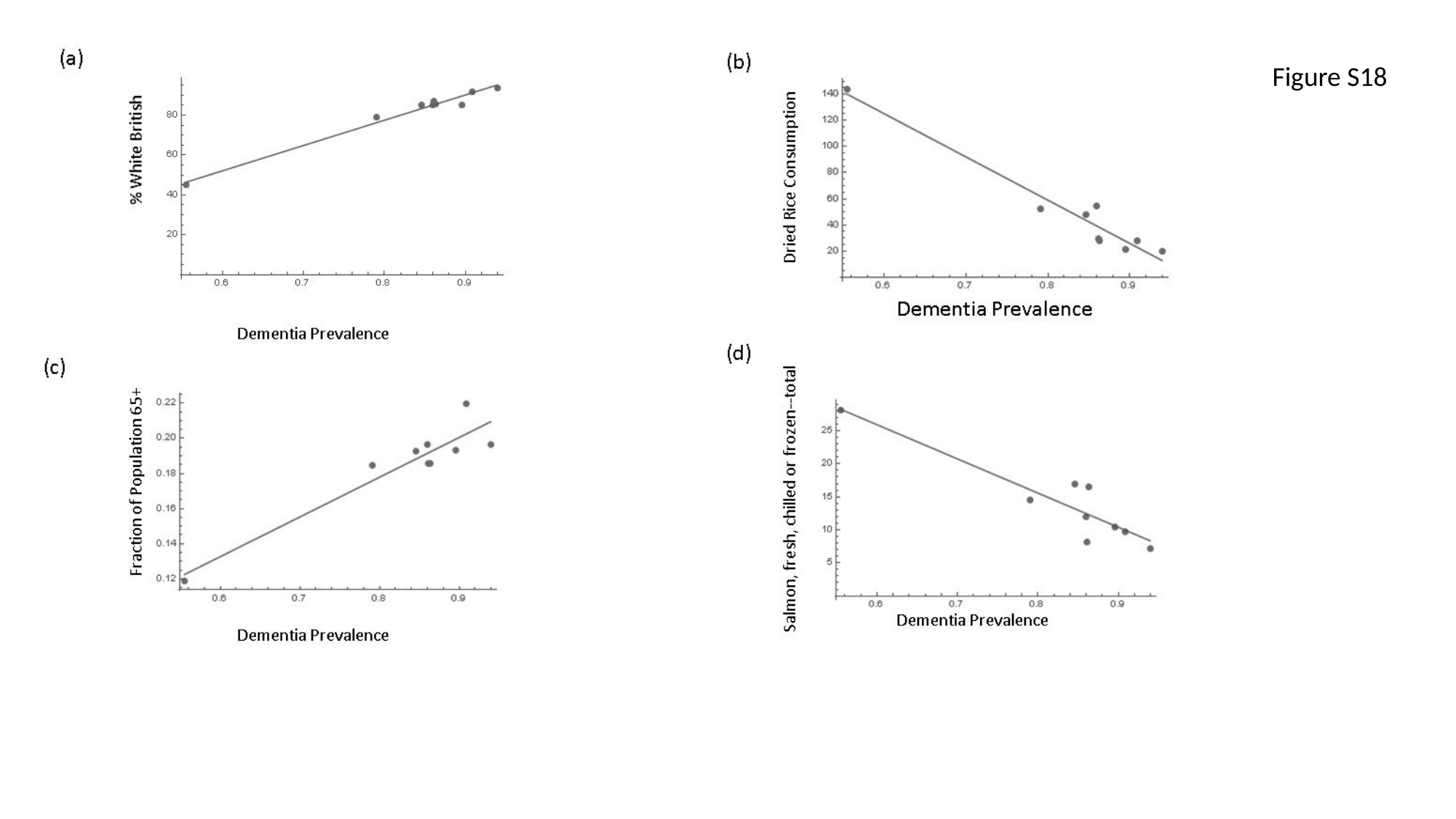

Figure S18

## Slide 23
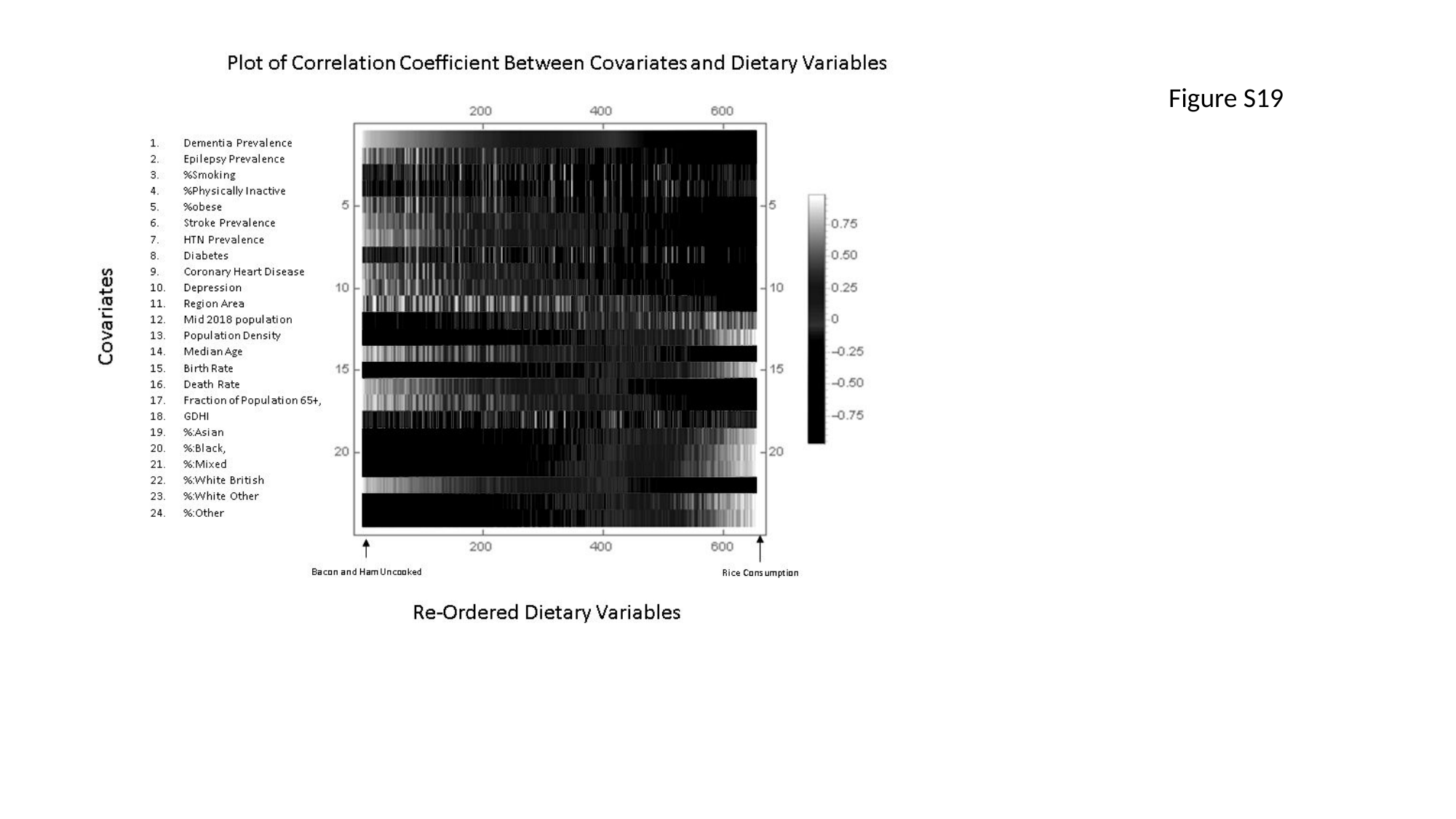

Figure S19

## Slide 24
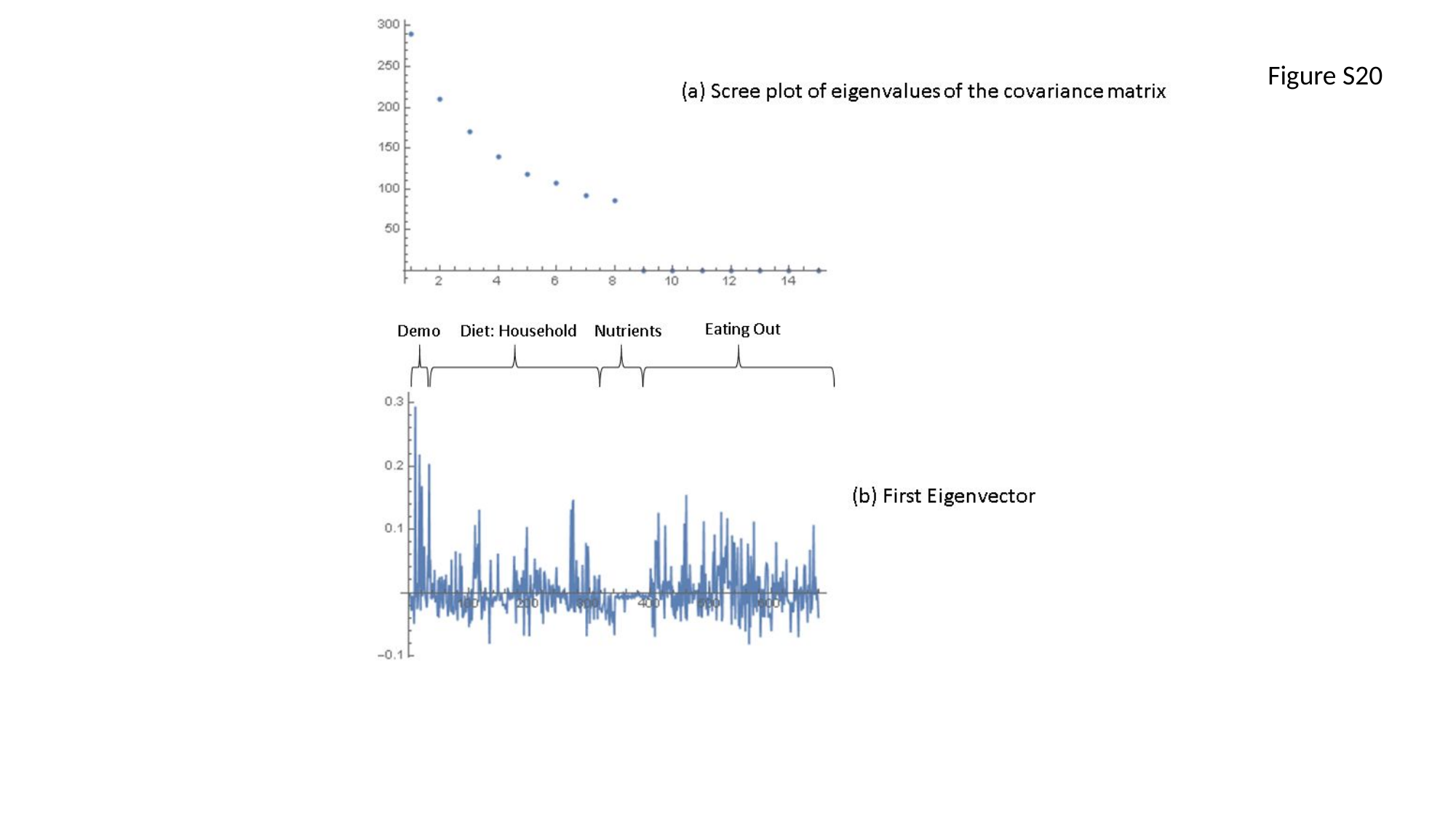

Figure S20

## Slide 25
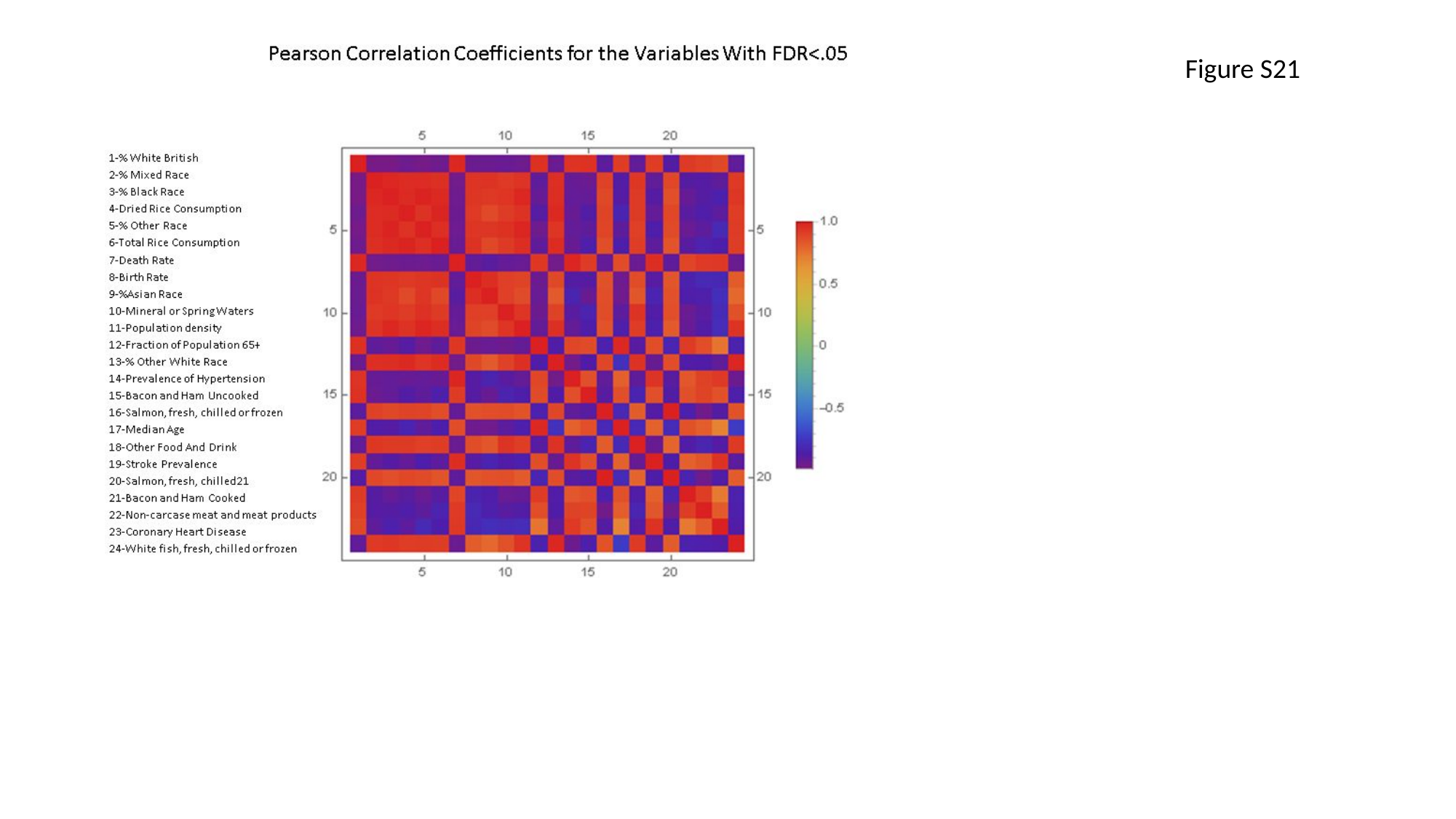

Figure S21

## Slide 26
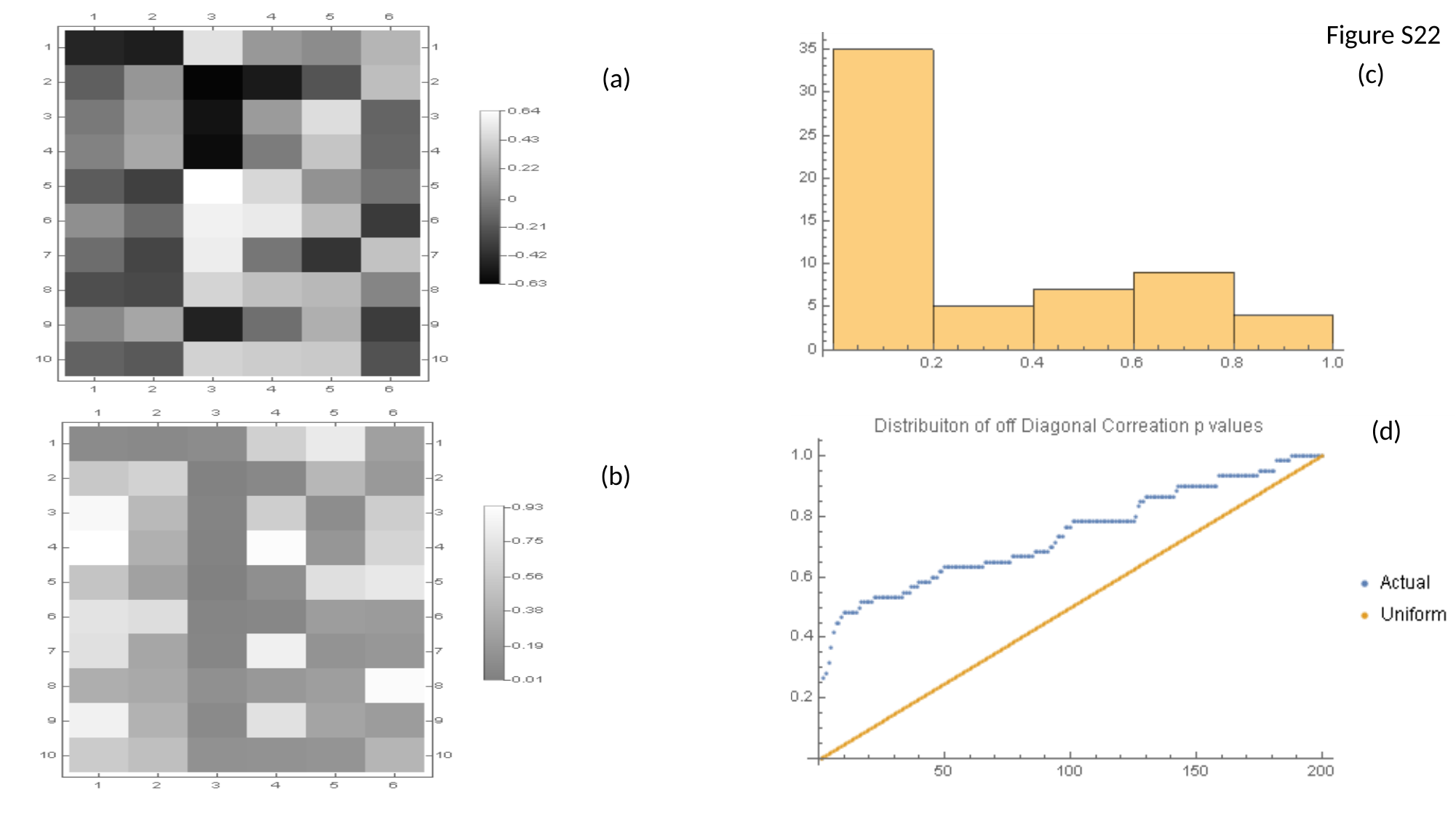

Figure S22
(c)
(a)
(d)
(b)

## Slide 27
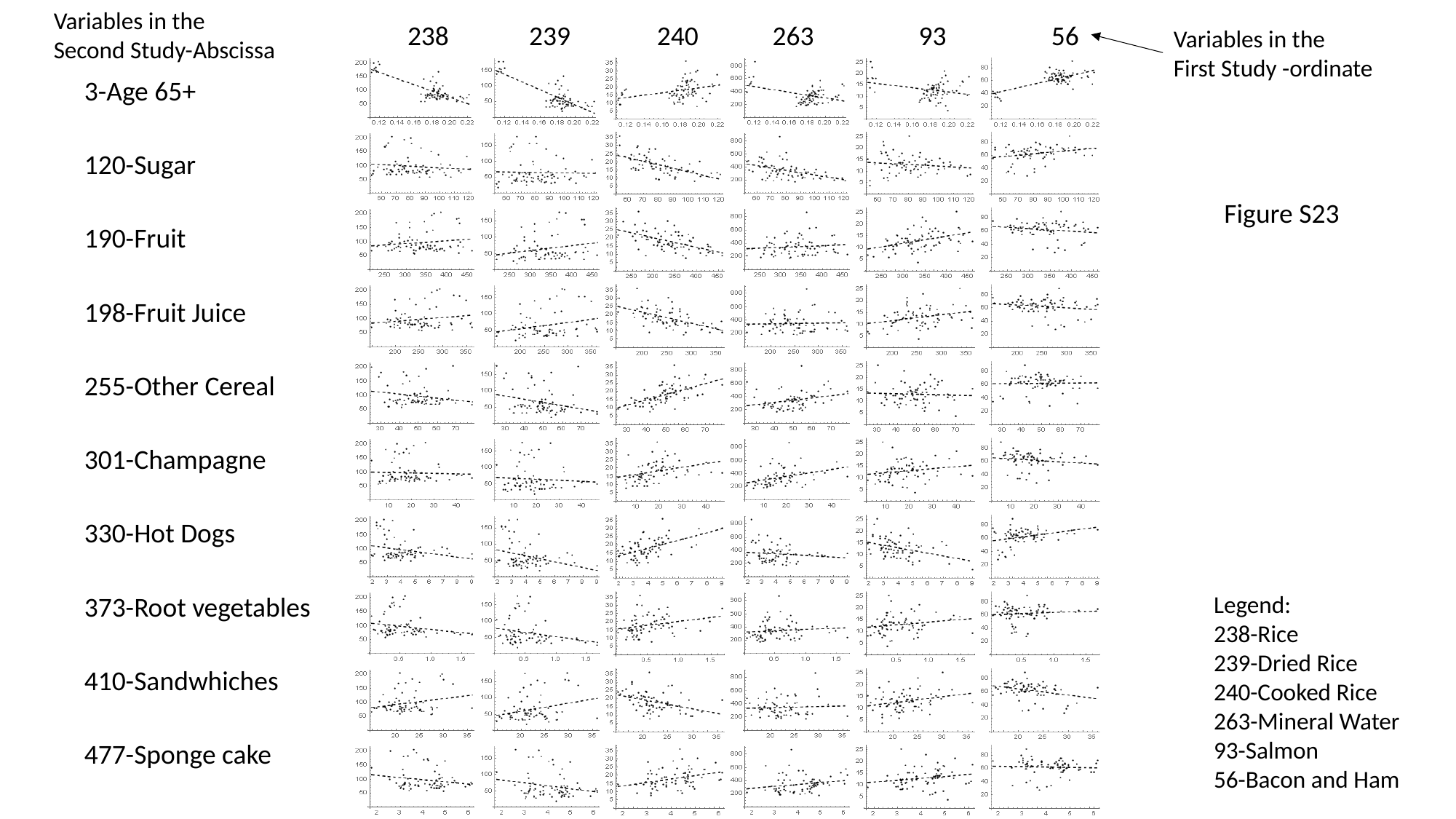

Variables in the
Second Study-Abscissa
238 239 240 263 93 56
Variables in the
First Study -ordinate
3-Age 65+
120-Sugar
190-Fruit
198-Fruit Juice
255-Other Cereal
301-Champagne
330-Hot Dogs
373-Root vegetables
410-Sandwhiches
477-Sponge cake
Figure S23
Legend:
238-Rice
239-Dried Rice
240-Cooked Rice
263-Mineral Water
93-Salmon
56-Bacon and Ham

## Slide 28
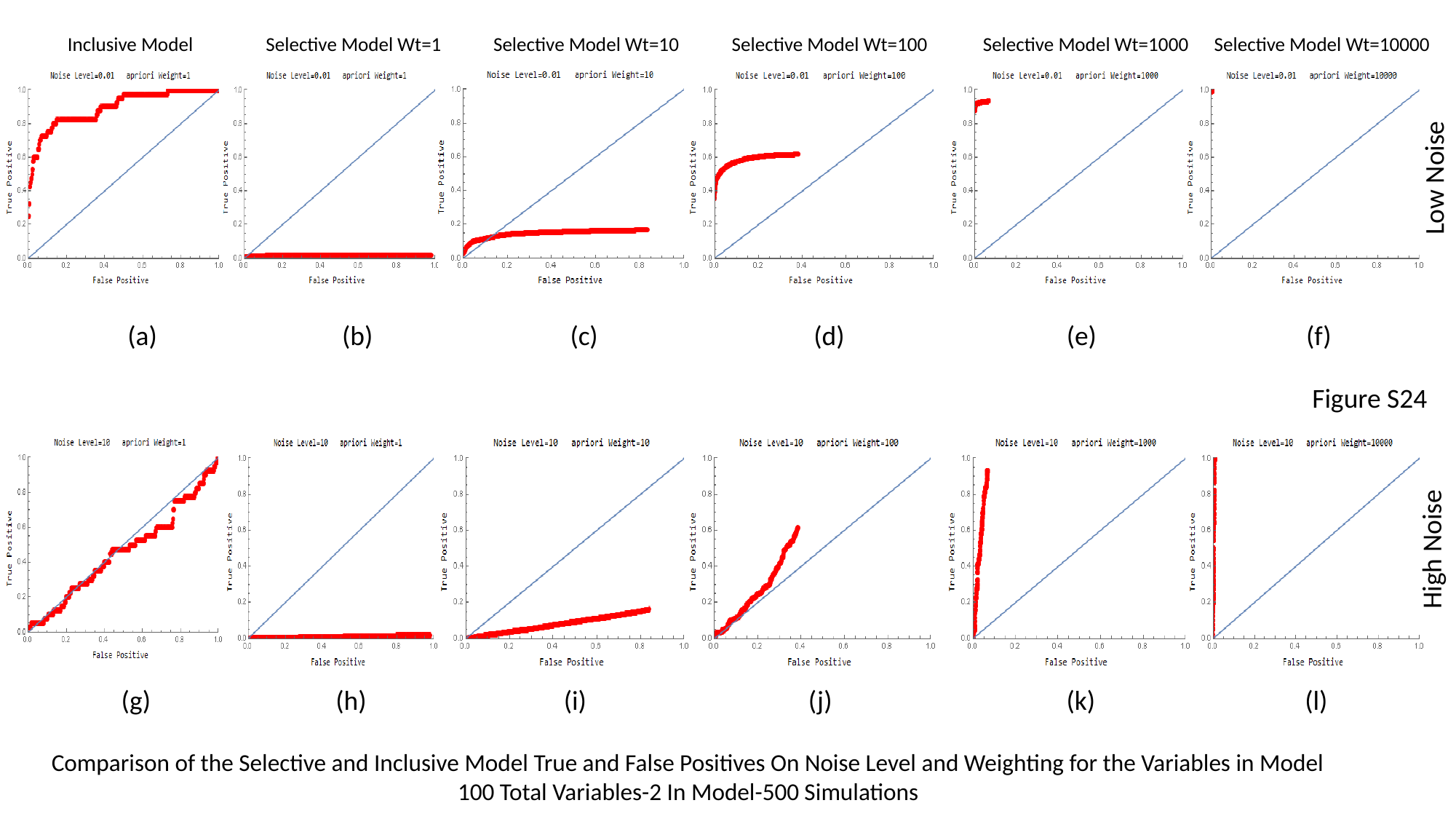

Selective Model Wt=1000
Inclusive Model
Selective Model Wt=1
Selective Model Wt=10
Selective Model Wt=100
Selective Model Wt=10000
Low Noise
(a) (b) (c) (d) (e) (f)
Figure S24
High Noise
(g) (h) (i) (j) (k) (l)
Comparison of the Selective and Inclusive Model True and False Positives On Noise Level and Weighting for the Variables in Model
100 Total Variables-2 In Model-500 Simulations

## Slide 29
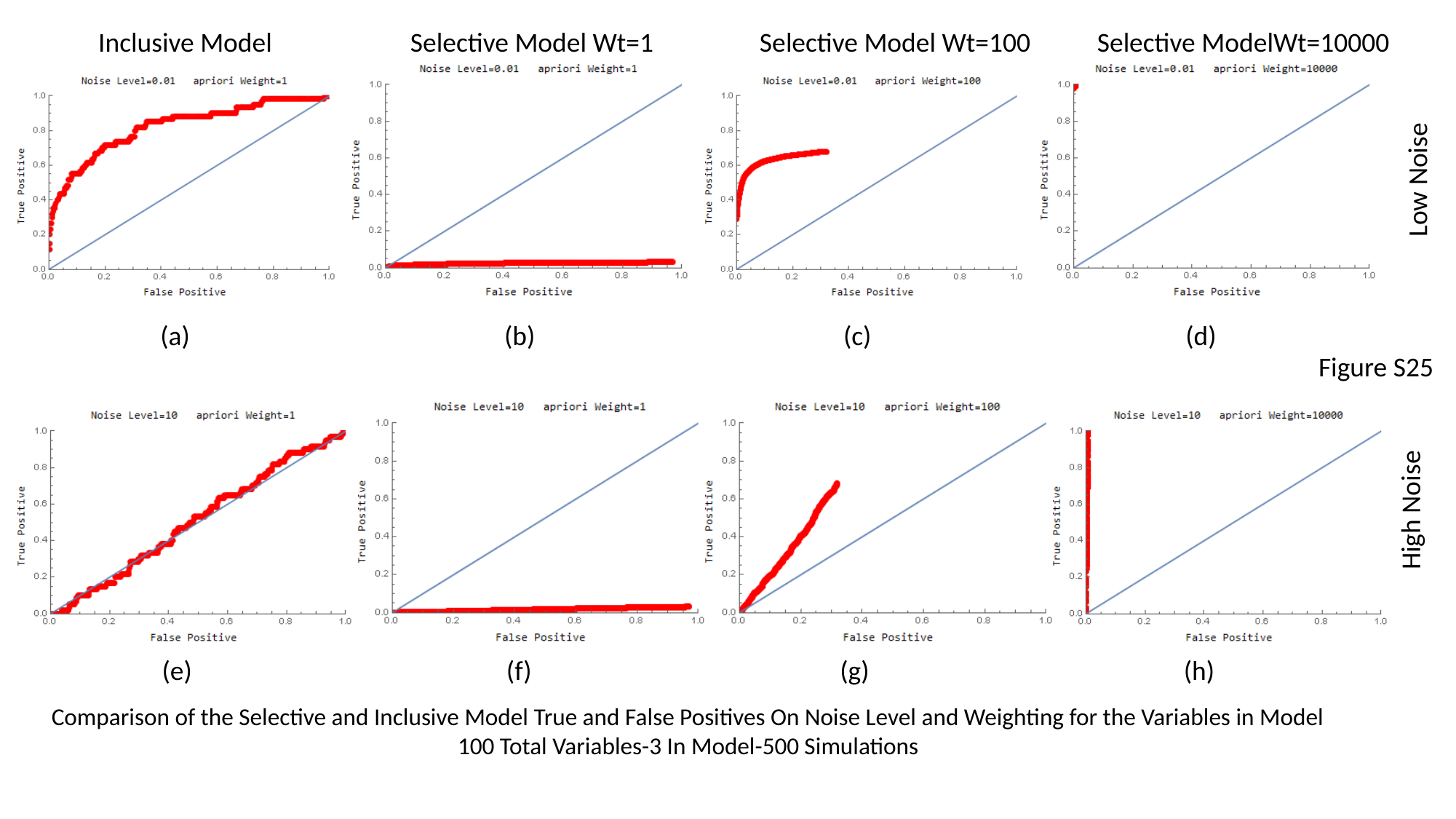

Inclusive Model
Selective Model Wt=1
Selective Model Wt=100
Selective ModelWt=10000
Low Noise
(a) (b) (c) (d)
Figure S25
High Noise
(e) (f) (g) (h)
Comparison of the Selective and Inclusive Model True and False Positives On Noise Level and Weighting for the Variables in Model
100 Total Variables-3 In Model-500 Simulations

## Slide 30
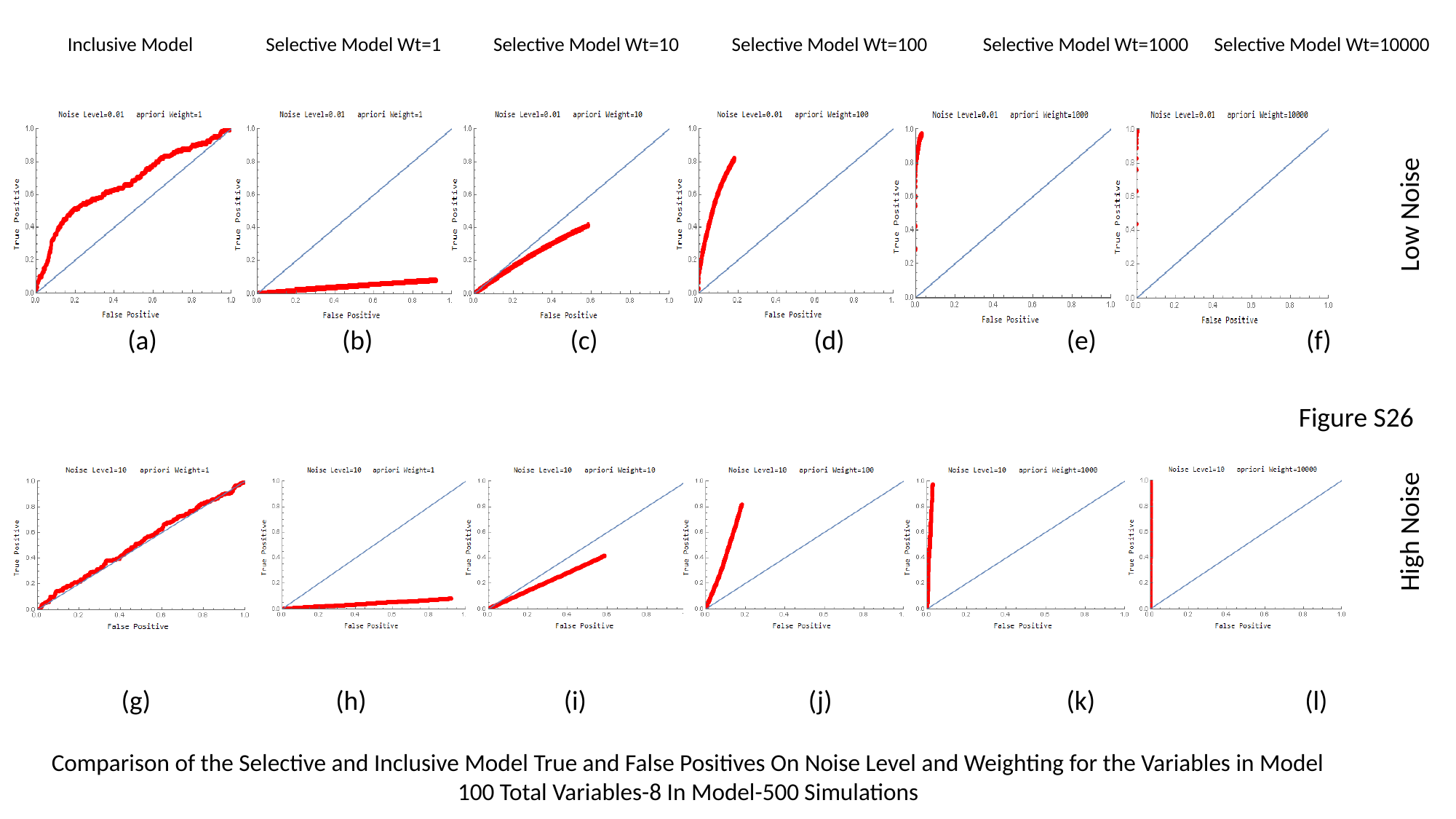

Selective Model Wt=1000
Inclusive Model
Selective Model Wt=1
Selective Model Wt=10
Selective Model Wt=100
Selective Model Wt=10000
Low Noise
(a) (b) (c) (d) (e) (f)
Figure S26
High Noise
(g) (h) (i) (j) (k) (l)
Comparison of the Selective and Inclusive Model True and False Positives On Noise Level and Weighting for the Variables in Model
100 Total Variables-8 In Model-500 Simulations

## Slide 31
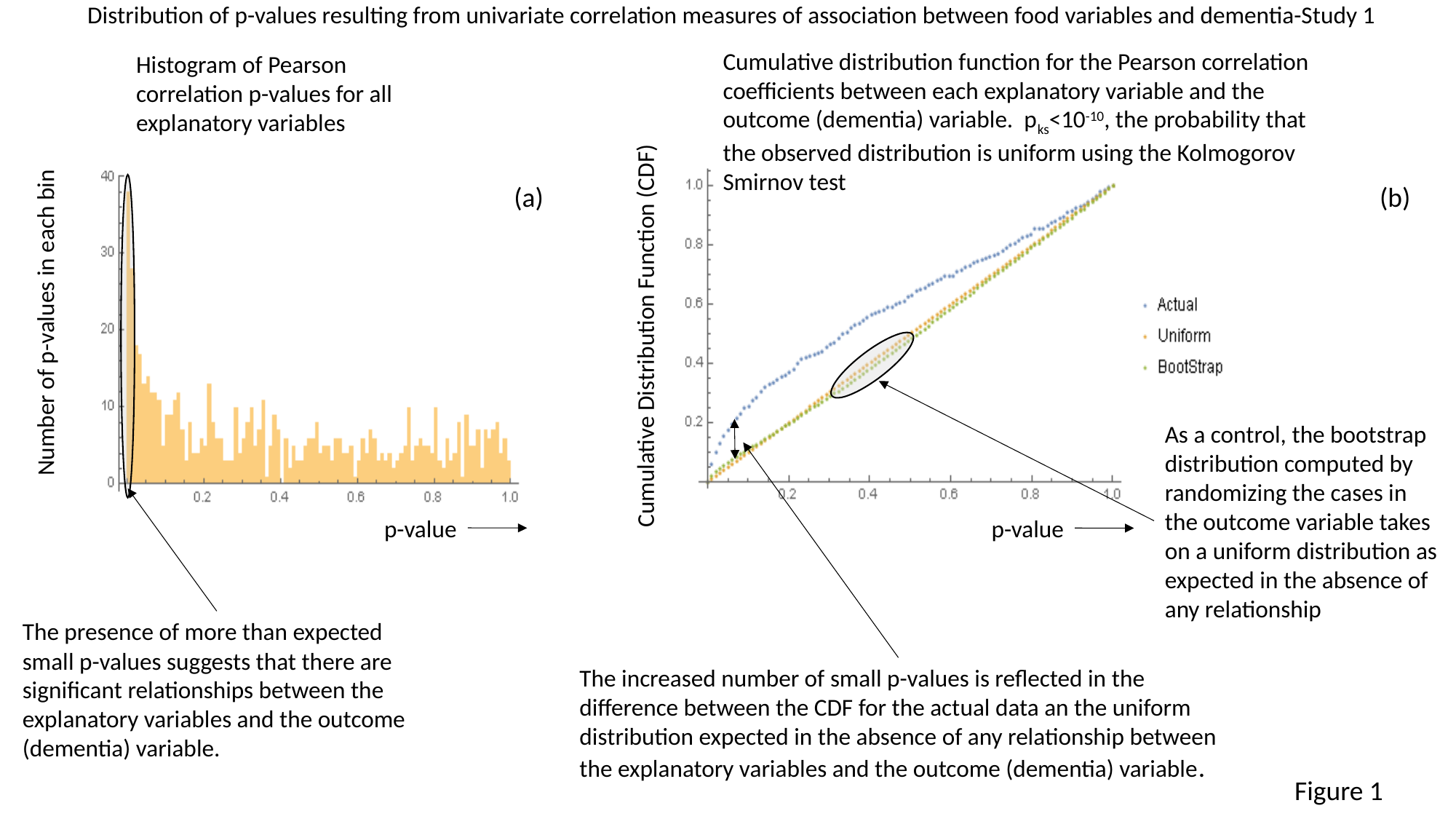

Distribution of p-values resulting from univariate correlation measures of association between food variables and dementia-Study 1
Cumulative distribution function for the Pearson correlation coefficients between each explanatory variable and the outcome (dementia) variable. pks<10-10, the probability that the observed distribution is uniform using the Kolmogorov Smirnov test
Histogram of Pearson
correlation p-values for all
explanatory variables
(b)
(a)
Number of p-values in each bin
Cumulative Distribution Function (CDF)
As a control, the bootstrap
distribution computed by
randomizing the cases in
the outcome variable takes
on a uniform distribution as
expected in the absence of
any relationship
p-value
p-value
The presence of more than expected
small p-values suggests that there are
significant relationships between the
explanatory variables and the outcome
(dementia) variable.
The increased number of small p-values is reflected in the
difference between the CDF for the actual data an the uniform
distribution expected in the absence of any relationship between
the explanatory variables and the outcome (dementia) variable.
Figure 1

## Slide 32
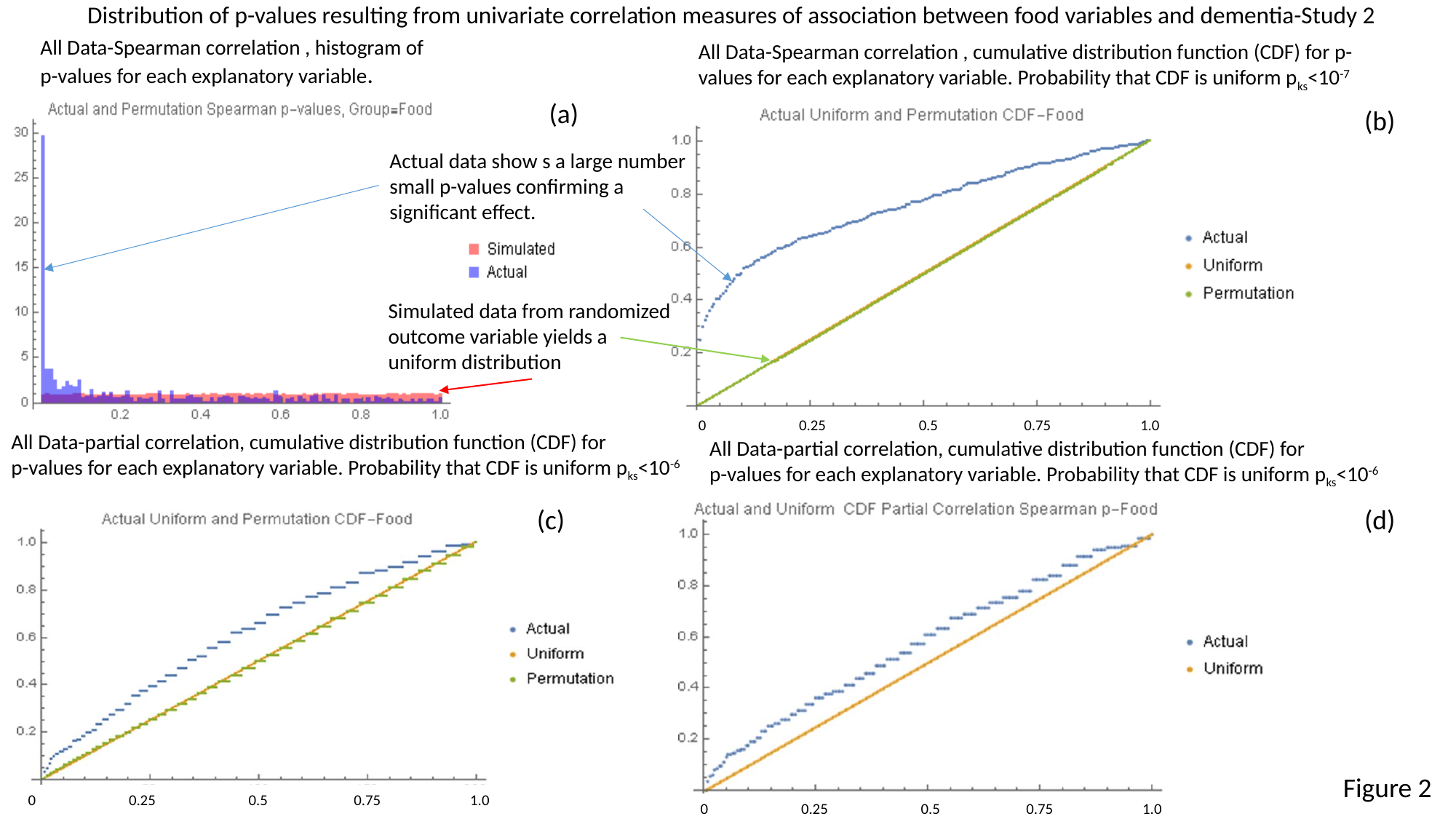

Distribution of p-values resulting from univariate correlation measures of association between food variables and dementia-Study 2
All Data-Spearman correlation , histogram of p-values for each explanatory variable.
All Data-Spearman correlation , cumulative distribution function (CDF) for p-values for each explanatory variable. Probability that CDF is uniform pks<10-7
(a)
(b)
Actual data show s a large number
small p-values confirming a
significant effect.
Simulated data from randomized
outcome variable yields a
uniform distribution
0 0.25 0.5 0.75 1.0
All Data-partial correlation, cumulative distribution function (CDF) for
p-values for each explanatory variable. Probability that CDF is uniform pks<10-6
All Data-partial correlation, cumulative distribution function (CDF) for
p-values for each explanatory variable. Probability that CDF is uniform pks<10-6
(c)
(d)
Figure 2
0 0.25 0.5 0.75 1.0
0 0.25 0.5 0.75 1.0

## Slide 33
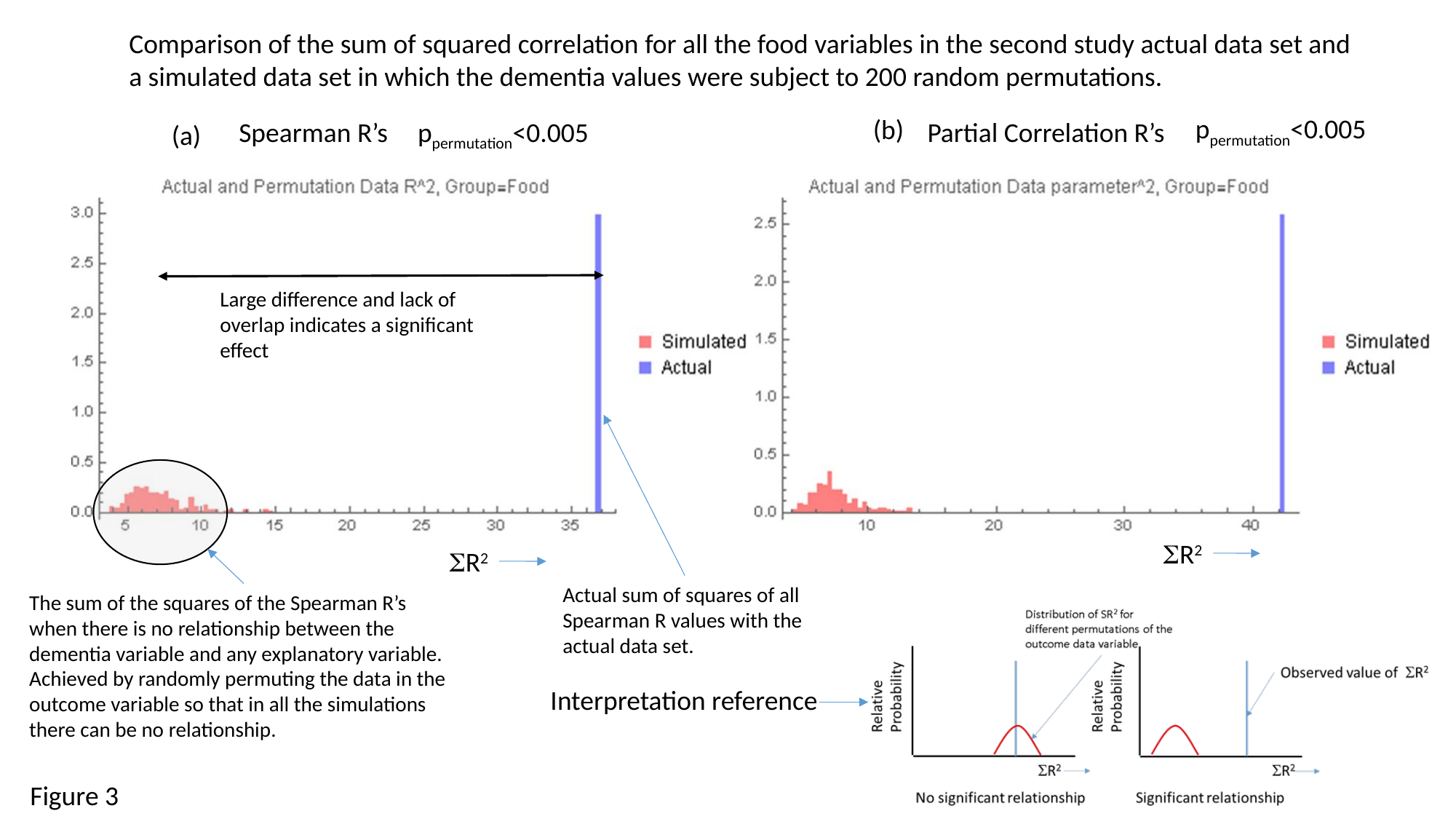

Comparison of the sum of squared correlation for all the food variables in the second study actual data set and
a simulated data set in which the dementia values were subject to 200 random permutations.
ppermutation<0.005
(b)
Spearman R’s
ppermutation<0.005
Partial Correlation R’s
(a)
Large difference and lack of
overlap indicates a significant
effect
SR2
SR2
Actual sum of squares of all
Spearman R values with the
actual data set.
The sum of the squares of the Spearman R’s when there is no relationship between the dementia variable and any explanatory variable.
Achieved by randomly permuting the data in the outcome variable so that in all the simulations there can be no relationship.
Interpretation reference
Figure 3

## Slide 34
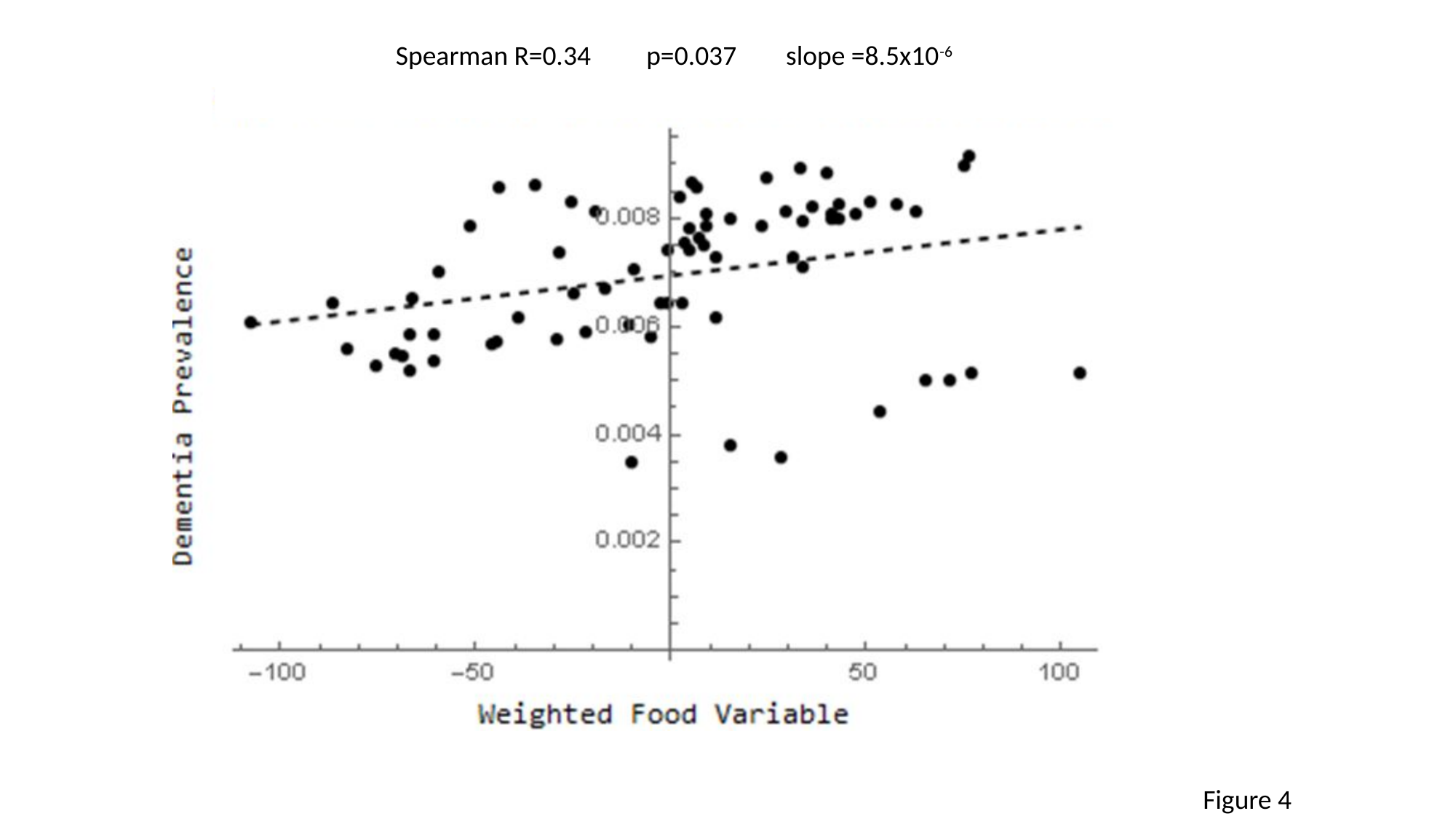

Spearman R=0.34 p=0.037 slope =8.5x10^-6^
Figure 4

## Slide 35
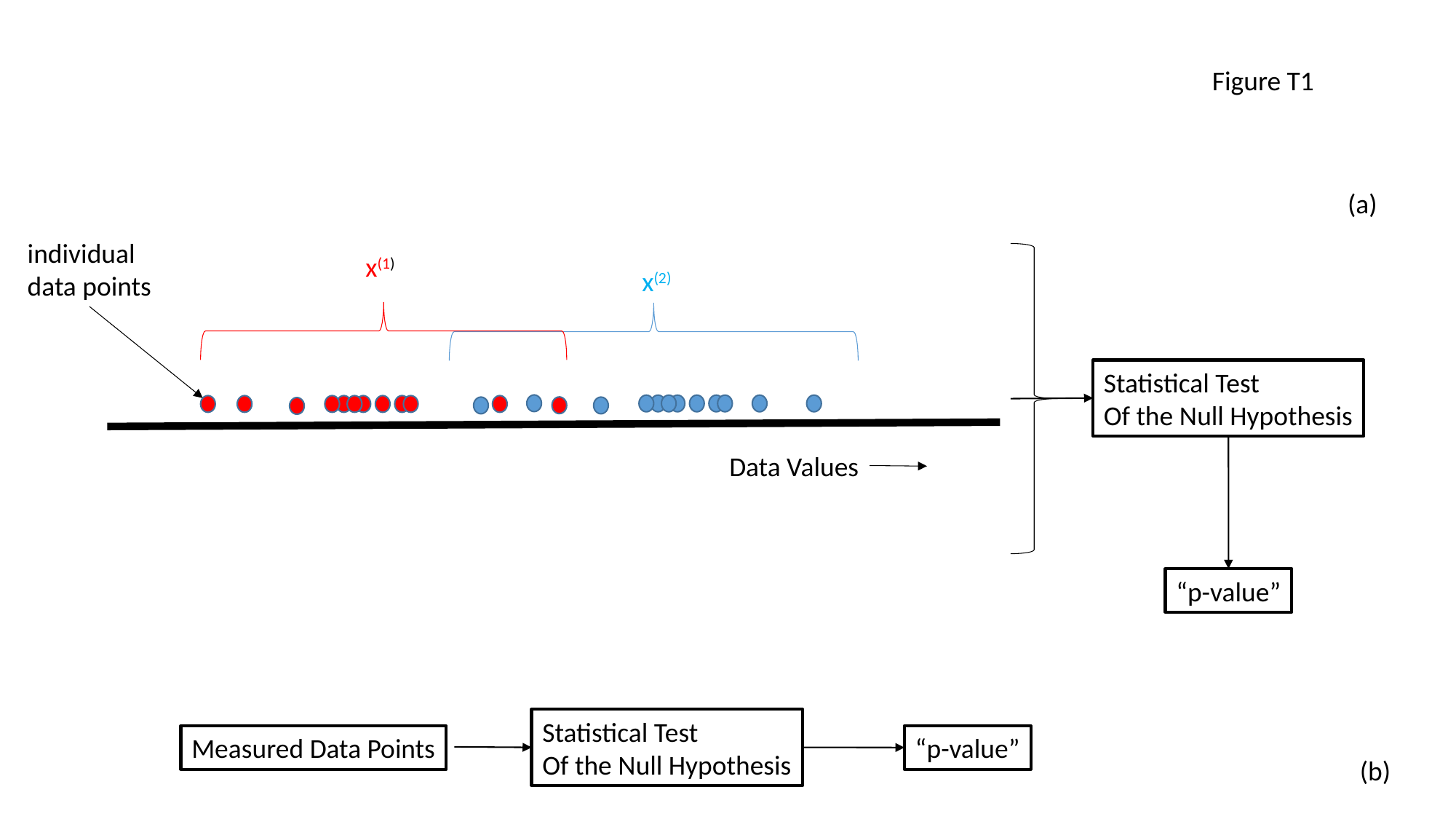

Figure T1
(a)
individual
data points
x(1)
x(2)
Statistical Test
Of the Null Hypothesis
Data Values
“p-value”
Statistical Test
Of the Null Hypothesis
Measured Data Points
“p-value”
(b)

## Slide 36
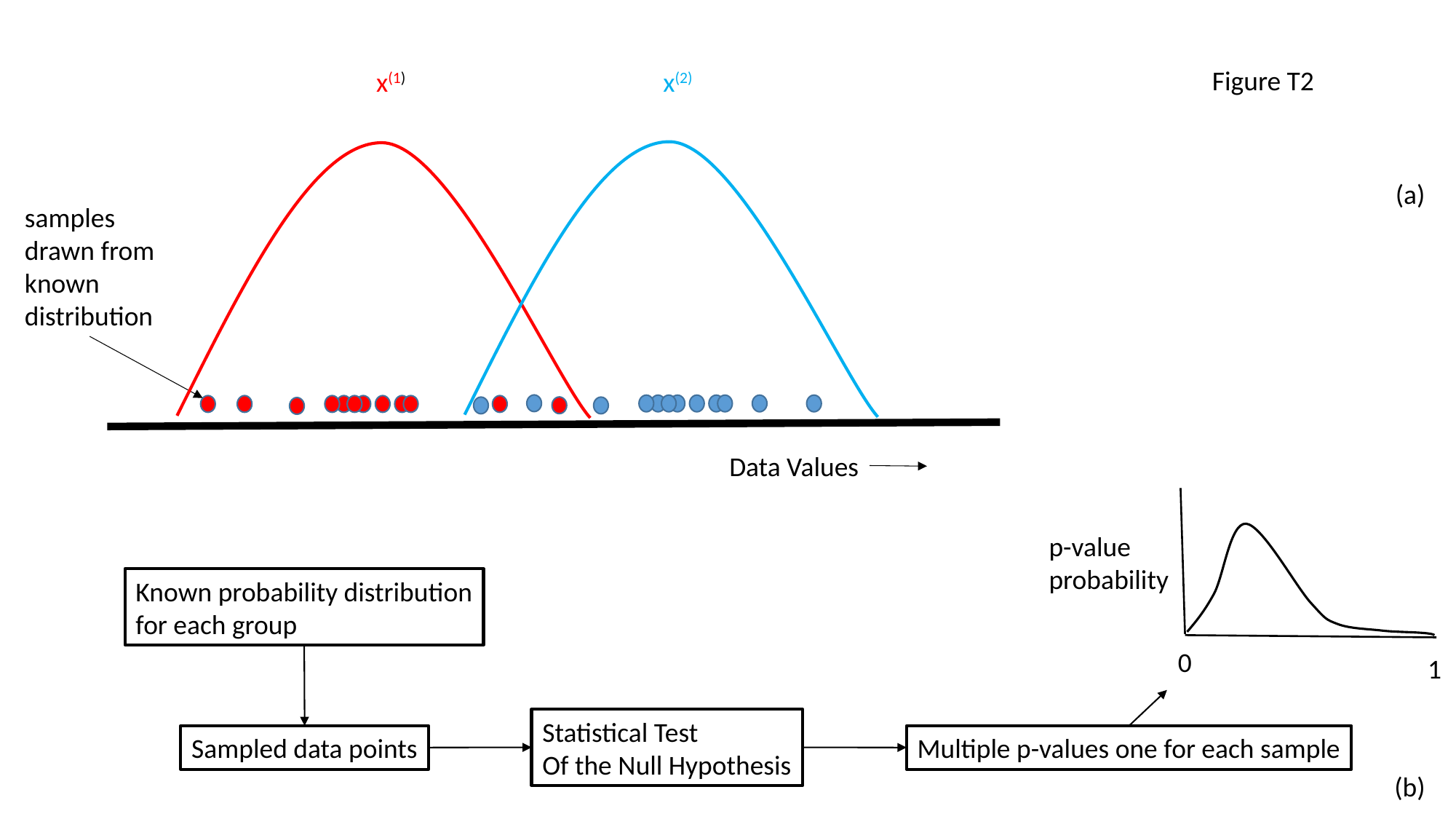

Figure T2
x(1)
x(2)
(a)
samples
drawn from
known
distribution
Data Values
p-value
probability
Known probability distribution
for each group
0
1
Statistical Test
Of the Null Hypothesis
Sampled data points
Multiple p-values one for each sample
(b)

## Slide 37
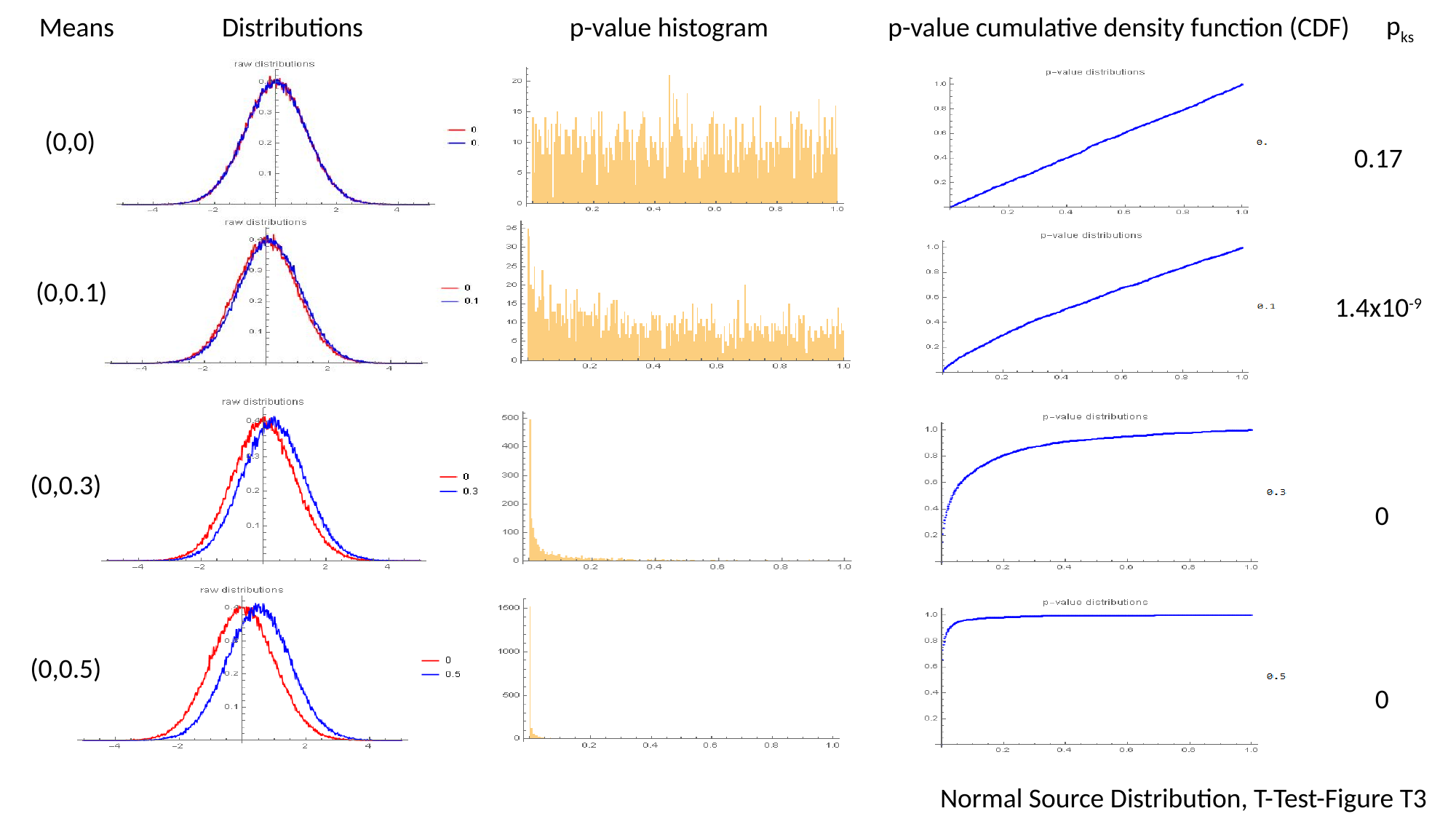

pks
Means
Distributions
p-value histogram
p-value cumulative density function (CDF)
(0,0)
0.17
(0,0.1)
1.4x10^-9^
(0,0.3)
0
(0,0.5)
0
Normal Source Distribution, T-Test-Figure T3

## Slide 38
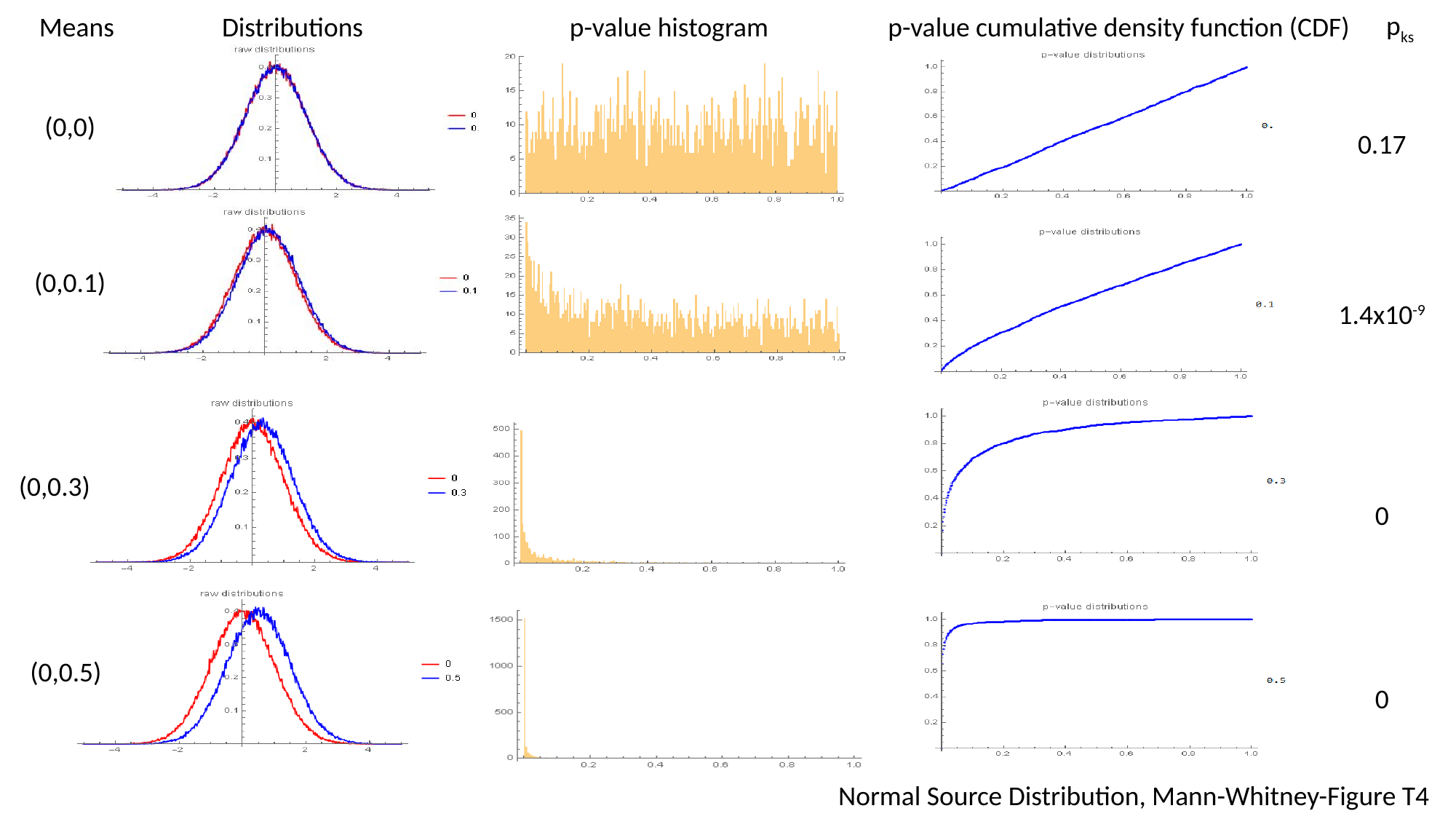

pks
Means
Distributions
p-value histogram
p-value cumulative density function (CDF)
(0,0)
0.17
(0,0.1)
1.4x10^-9^
(0,0.3)
0
(0,0.5)
0
Normal Source Distribution, Mann-Whitney-Figure T4

## Slide 39
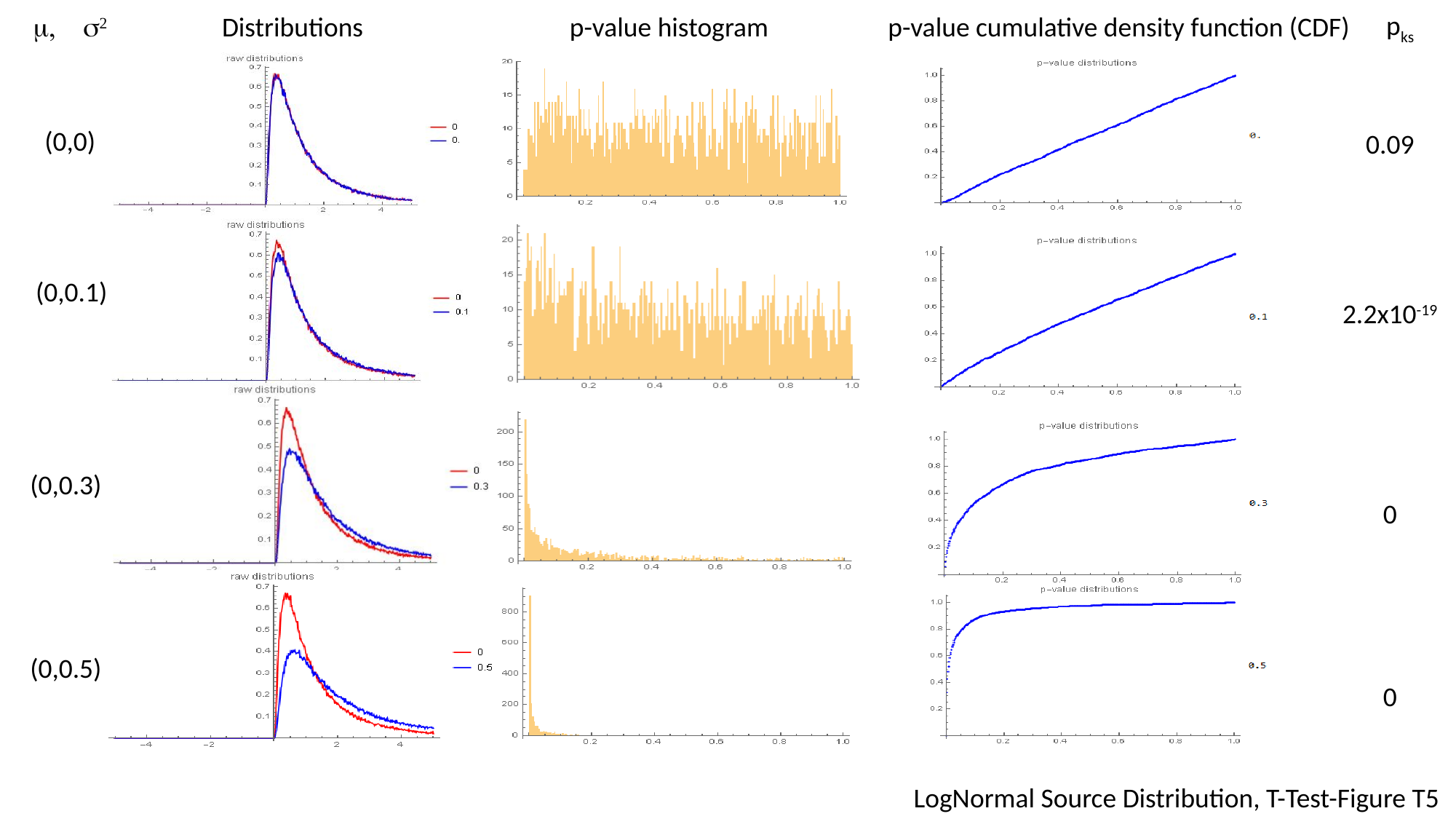

pks
m, s2
Distributions
p-value histogram
p-value cumulative density function (CDF)
(0,0)
0.09
(0,0.1)
2.2x10^-19^
(0,0.3)
0
(0,0.5)
0
LogNormal Source Distribution, T-Test-Figure T5

## Slide 40
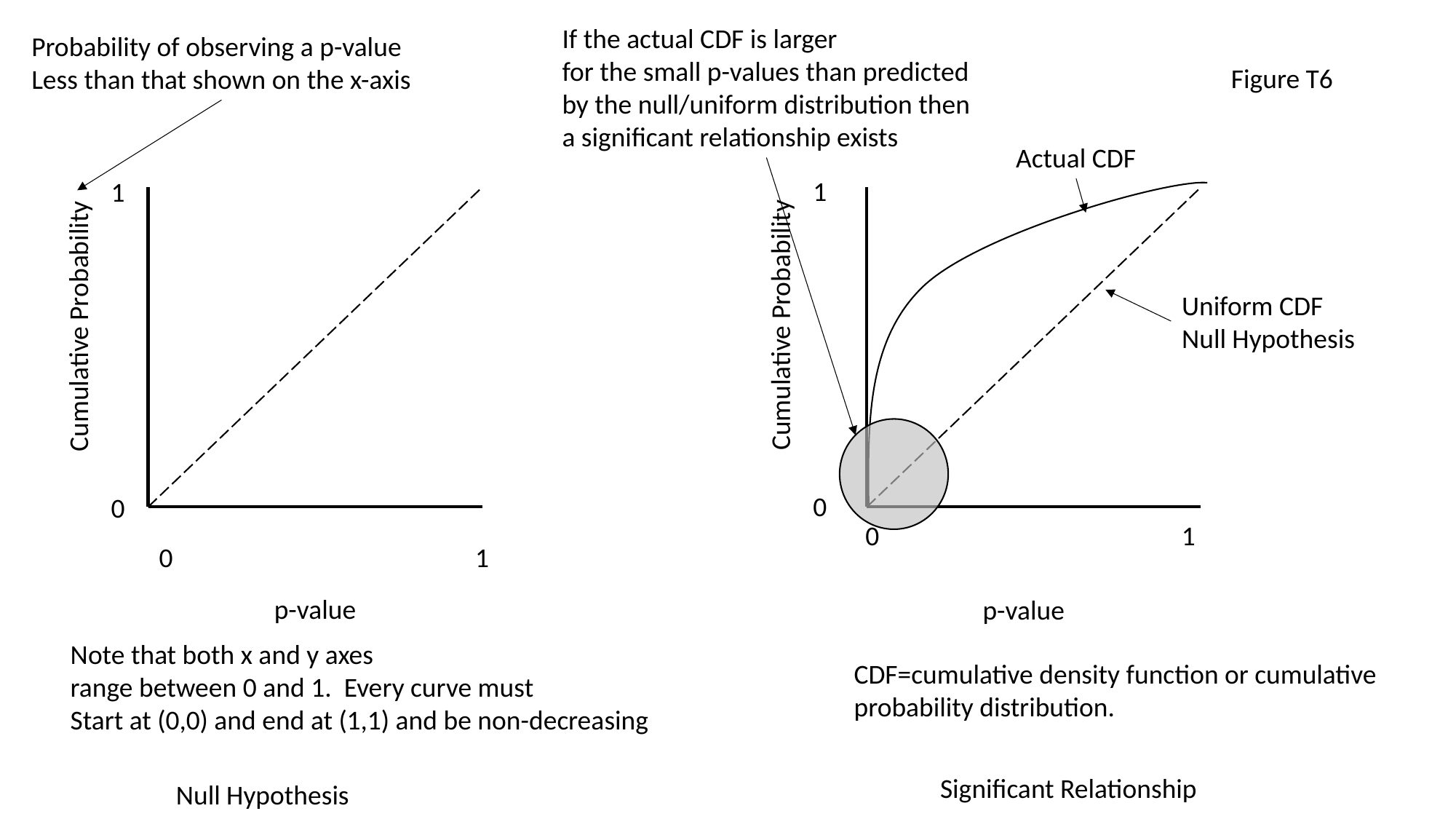

If the actual CDF is larger
for the small p-values than predicted
by the null/uniform distribution then
a significant relationship exists
Probability of observing a p-value
Less than that shown on the x-axis
Figure T6
Actual CDF
1
1
Uniform CDF
Null Hypothesis
Cumulative Probability
Cumulative Probability
0
0
0
1
0
1
p-value
p-value
Note that both x and y axes
range between 0 and 1. Every curve must
Start at (0,0) and end at (1,1) and be non-decreasing
CDF=cumulative density function or cumulative
probability distribution.
Significant Relationship
Null Hypothesis
